# Supplementary material for: Epidemiological characteristics and transmission dynamics of dengue fever in China
Source: Nat Commun. 2024 Sep 14;15:8060. doi: 10.1038/s41467-024-52460-w (PMC11401889; doi:10.1038/s41467-024-52460-w)
Supplement: Supplementary file 1 — Supplementary Information [file 41467_2024_52460_MOESM1_ESM.pdf]

Supplementary Information

# Epidemiological characteristics and transmission dynamics of dengue fever in China


**Authors**

Haobo Ni [1,†], Xiaoyan Cai [1,†], Jiarong Ren [2,†], Tingting Dai [1], Jiayi Zhou [1], Jiumin Lin [3], Li Wang [4], Lingxi Wang [1], Sen Pei [5], Yunchong Yao [1], Ting Xu [1], Lina Xiao [1], Qiyong Liu [2,6,7,*], Xiaobo Liu [2,6,7,*], Pi Guo [1,*]

[1] Department of Preventive Medicine, Shantou University Medical College, Shantou, China

[2] National Key Laboratory of Intelligent Tracking and Forecasting for Infectious Diseases, National Institute for Communicable Disease Control and Prevention, Chinese Center for Disease Control and Prevention, Beijing, China

[3] Department of Hepatology and Infectious Diseases, Second Affiliated Hospital of Shantou University Medical College, Shantou, China

[4] Department of Medical Statistics and Epidemiology, School of Public Health, Sun Yat-sen University, Guangzhou, China

[5] Department of Environmental Health Sciences, Mailman School of Public Health, Columbia University, New York, New York, United States of America

[6] Department of Vector Control, School of Public Health, Cheeloo College of Medicine, Shandong University, Jinan, China

[7] Xinjiang Key Laboratory of Vector-borne Infectious Diseases, Urumqi, Xinjiang, China

[†] These authors contributed equally to this work.

[*] **Corresponding author:**

Pi Guo

Department of Preventive Medicine, Shantou University Medical College, Shantou 515041, China

Email: pguo@stu.edu.cn

Xiaobo Liu

National Key Laboratory of Intelligent Tracking and Forecasting for Infectious Diseases, National Institute for Communicable Disease Control and Prevention, Chinese Center for Disease Control and Prevention, Beijing 102206, China; Department of Vector Control, School of Public Health, Cheeloo College of Medicine, Shandong University, Jinan 250012, China; Xinjiang Key Laboratory of Vector-borne Infectious Diseases, Urumqi, Xinjiang 830002, China

Email: liuxiaobo@icdc.cn

Qiyong Liu

National Key Laboratory of Intelligent Tracking and Forecasting for Infectious Diseases, National Institute for Communicable Disease Control and Prevention, Chinese Center for Disease Control and Prevention, Beijing 102206, China; Department of Vector Control, School of Public Health, Cheeloo College of Medicine, Shandong University, Jinan 250012, China; Xinjiang Key Laboratory of Vector-borne Infectious Diseases, Urumqi, Xinjiang 830002, China

Email: liuqiyong@icdc.cn

## Table of Contents

**TABLE OF CONTENTS** ........ **3**

**DENGUE FEVER DIAGNOSIS IN MAINLAND CHINA** ........ **6**

**ADDITIONAL INFORMATION REGARDING THE ESTIMATION OF SEASONAL PARAMETERS** . **11**

**DENGUE FEVER TRANSMISSION MODEL** ........ **12**

**MODEL CALIBRATION** ........ **16**

**URBAN AGGLOMERATIONS** ........ **20**

**SENSITIVITY ANALYSIS** ........ **21**

**SUPPLEMENTARY FIGURE 1. MAP OF THE 31 PROVINCIAL ADMINISTRATIONS IN MAINLAND CHINA INCLUDED IN THIS ANALYSIS (CHINA HAS A TOTAL OF 34 PROVINCIAL ADMINISTRATION).** ........ **22**

**SUPPLEMENTARY FIGURE 2. SPATIO-TEMPORAL DISTRIBUTION FOR LOCAL CASES OF DENGUE FEVER IN MAINLAND CHINA FROM 2013 TO 2020.** ........ **23**

**SUPPLEMENTARY FIGURE 3. SPATIO-TEMPORAL DISTRIBUTION FOR IMPORTED CASES OF DENGUE FEVER IN MAINLAND CHINA FROM 2013 TO 2020.** ........ **24**

**SUPPLEMENTARY FIGURE 4. THE SOURCE-SINK RELATIONSHIP OF DENGUE FEVER IN MAINLAND CHINA FROM 2013 TO 2020.** ........ **25**

**SUPPLEMENTARY FIGURE 5. THE SOURCE-SINK RELATIONSHIP OF DENGUE FEVER IN PROVINCE WITH LOW IMPORTED CASES (LESS THAN 1%).** ........ **26**

**SUPPLEMENTARY FIGURE 6. NIGHTINGALE ROSE CHART FOR AGE DISTRIBUTION OF LOCAL DENGUE CASES IN PROVINCES OF MAINLAND CHINA FROM 2013 TO 2020.** ........ **27**

**SUPPLEMENTARY FIGURE 7. ONSET-TO-DIAGNOSIS DISTRIBUTIONS OF DENGUE FEVER CASES WITH DIFFERENT ONSET TIME AND DIFFERENT AGES.** ........ **28**

**SUPPLEMENTARY FIGURE 8. THE GEOGRAPHICAL LOCATION OF METEOROLOGICAL MONITORING STATIONS (DURING 2013-2020) AND MOSQUITO VECTOR DATA SURVEY SITES (DURING 2013-2020) IN CHINA.** ........ **29**

**SUPPLEMENTARY FIGURE 9. DISTRIBUTION OF MOSQUITO VECTORS IN EACH PROVINCIAL ADMINISTRATION.** ........ **30**

**SUPPLEMENTARY FIGURE 10. THE AVERAGE ANNUAL AMBIENT TEMPERATURE (℃) IN MAINLAND CHINA FROM 2013 TO 2019.** ........ **31**

**SUPPLEMENTARY FIGURE 11. THE FIT OF PROVINCE-SPECIFIC SEASONAL MODELS FOR LOCAL DENGUE CASE IN BEIJING, TIANJIN, HEBEI, SHANXI, INNER MONGOLIA, LIAONING, JILIN, HEILONGJIANG AND SHANGHAI.** ........ **32**

**SUPPLEMENTARY FIGURE 12. THE FIT OF PROVINCE-SPECIFIC SEASONAL MODELS FOR LOCAL DENGUE CASE IN JIANGSU, ZHEJIANG, ANHUI, FUJIAN, JIANGXI, SHANDONG, HENAN, HUBEI AND HUNAN.** ........ **33**

**SUPPLEMENTARY FIGURE 13. THE FIT OF PROVINCE-SPECIFIC SEASONAL MODELS FOR LOCAL DENGUE CASE IN GUANGDONG, GUANGXI, HAINAN, CHONGQING, SICHUAN,**

GUIZHOU, YUNNAN, TIBET AND SHAANXI. 34
SUPPLEMENTARY FIGURE 14. THE FIT OF PROVINCE-SPECIFIC SEASONAL MODELS FOR LOCAL DENGUE CASE IN GANSU, QINGHAI, NINGXIA AND XINJIANG. 35
SUPPLEMENTARY FIGURE 15. THE FIT OF PROVINCE-SPECIFIC MODELS FOR IMPORTED DENGUE CASE IN BEIJING, TIANJIN, HEBEI, SHANXI, INNER MONGOLIA, LIAONING, JILIN, HEILONGJIANG AND SHANGHAI. 36
SUPPLEMENTARY FIGURE 16. THE FIT OF PROVINCE-SPECIFIC SEASONAL MODELS FOR IMPORTED DENGUE CASE IN JIANGSU, ZHEJIANG, ANHUI, FUJIAN, JIANGXI, SHANDONG, HENAN, HUBEI AND HUNAN. 37
SUPPLEMENTARY FIGURE 17. THE FIT OF PROVINCE-SPECIFIC SEASONAL MODELS FOR IMPORTED DENGUE CASE IN GUANGDONG, GUANGXI, HAINAN, CHONGQING, SICHUAN, GUIZHOU, YUNNAN, TIBET AND SHAANXI. 38
SUPPLEMENTARY FIGURE 18. THE FIT OF PROVINCE-SPECIFIC SEASONAL MODELS FOR IMPORTED DENGUE CASE IN GANSU, QINGHAI, NINGXIA AND XINJIANG. 39
SUPPLEMENTARY FIGURE 19. LATITUDINAL GRADIENTS IN SEASONAL EPIDEMIC OF IMPORTED DENGUE CASES IN THE 31 PROVINCIAL ADMINISTRATIONS IN MAINLAND CHINA. 40
SUPPLEMENTARY FIGURE 20. THE FLOWCHART OF MODEL CONSTRUCTION 41
SUPPLEMENTARY FIGURE 21. THE VARIATIONS OF TRANSMISSION RATE IN 337 CITIES IN MAINLAND CHINA. 42
SUPPLEMENTARY FIGURE 22. RELATIONSHIP BETWEEN COMMUTING INTENSITY AND ESTIMATED INFECTIONS DUE TO POPULATION MOVEMENTS AMONG CITIES IN CHINA, 2019. 43
SUPPLEMENTARY FIGURE 23. PRIOR RESULTS IN FOUR URBAN AGGLOMERATIONS AND IN NATIONAL SCALE. 44
SUPPLEMENTARY FIGURE 24. PARAMETER INFERENCE FOR SIMULATED OUTBREAKS. 45
SUPPLEMENTARY FIGURE 25. SENSITIVITY ANALYSES ON INFERENCE RESULTS. 46
SUPPLEMENTARY FIGURE 26. SENSITIVITY ANALYSES ON INFERENCE RESULTS. 47
SUPPLEMENTARY FIGURE 27. SENSITIVITY ANALYSES ON INFERENCE RESULTS. 48
SUPPLEMENTARY FIGURE 28. SENSITIVITY ANALYSES ON INFERENCE RESULTS. 49
SUPPLEMENTARY FIGURE 29. SENSITIVITY ANALYSES ON INFERENCE RESULTS. 50
SUPPLEMENTARY FIGURE 30. FITTING AND INFERENCE RESULTS OF DENGUE FEVER INFECTION IN CHINA FROM 2013 TO 2018. 51
TABLES 52
SUPPLEMENTARY TABLE 1. BACKGROUND CHARACTERISTICS OF 31 PROVINCIAL ADMINISTRATIONS IN MAINLAND CHINA. 52
SUPPLEMENTARY TABLE 2. THE MAIN METHOD RELEVANT FINDINGS OF THE STUDIES ABOUT DENGUE FEVER IN CHINA. 54
SUPPLEMENTARY TABLE 3. THE INFORMATION OF 337 CITIES IN CHINA DURING 2013-2020.

**......................................................................................................................................................... 59**

**SUPPLEMENTARY TABLE 4. TRANSMISSION MODEL AND PARAMETERS ESTIMATION RESULTS. ........................................................................................................ 72**

**REFERENCE: ............................................................................................................ 107**

## Dengue fever diagnosis in mainland China

In mainland China, the National Health Commission is responsible for establishing and regularly updating diagnostic criteria for various infectious diseases. During the study period (2013-2020), dengue fever was diagnosed according to the diagnostic criteria of dengue fever issued on February 28, 2008 and the updated dengue fever diagnostic criteria issued on March 6, 2018. The diagnostic criteria published in 2008 (criteria number: WS 216-2008) are as follows[1].

## Dengue fever disease diagnostic criteria prior to 2018

### 1 Diagnostic basis

### 1.1 Epidemiology history

1.1.1 The individuals have been to the dengue fever epidemic area within 14 days before the onset of the disease.

1.1.2 There was (were) case(s) of dengue fever reported within one month around living place or workplace (within a circle with radius of 100 metres).

### 1.2 Clinical manifestation

1.2.1 Acute onset with symptoms such as fever (24-36 hours up to 39℃to 40℃, with a few cases having bimodal fever), headache, orbital pain, muscle pain, bone and joint pain, and obvious fatigue. Some cases accompanied by facial, neck, chest flushing, conjunctival congestion, etc.

1.2.2 Rashes: It appears as diverse rashes (measles-like rashes, scarlatina-like rash, needle-tip-like hemorrhagic rash) or manifestation of “skin island”, which occur on days from five to seven of the disease. The rash is typically distributed on the trunk, then gradually extending to the neck and limbs, in some patients, extending to the face. The rash is usually accompanied by pruritus without desquamation, lasting for 3 to 5 days.

1.2.3 Bleeding tendency (tourniquet test positive) is mainly manifested 5 to 8 days before the course of the disease, which includes ecchymosis, purpura and bleeding at the injection site, gingival bleeding, gastrointestinal bleeding, hemoptysis, hematuria, vaginal bleeding, nasal

bleeding and other mucosal bleeding, and so on.

1.2.4 Severe cases may have massive gastrointestinal bleeding, thoracic and abdominal bleeding, or intracranial hemorrhage.

1.2.5 Liver enlargement, hydrothorax, and ascites.

1.2.6 Shock symptoms such as moist and cold skin, irritability, hypotension, pulse pressure less than 20 mmHg (2.7 kPa), undetectable blood pressure, or decreased urine volume.

**1.3 Laboratory examination**

1.3.1 Leukopenia.

1.3.2 Thrombocytopenia ($<100\times10^9$/L)

1.3.3 Blood concentration, for example, after volume expansion treatment, hematocrit increased by more than 20 % compared with the normal level, or the hematocrit decreased by more than 20 % compared with the baseline level; hypoalbuminemia, etc.

1.3.4 The titer of specific dengue IgG or IgM antibody in recovery phase is over four times or more higher than that in acute phase.

1.3.5 Dengue virus is isolated from serum, cerebrospinal fluid, blood cells or tissues of patients in acute phase.

1.3.6 The titer of serum specific IgG antibody in recovery phase is four times or more higher than that in acute phase.

1.3.7 Dengue virus gene sequence is detected by RT-PCR or real-time fluorescence quantitative PCR.

**2 Diagnosis principle**

The comprehensive diagnosis was made according to the epidemiological evidence, clinical manifestations and laboratory examination results of the patients.

**3 Diagnosis**

**3.1 Probable cases**

Consistent with the following one can be diagnosed as probable cases:

3.1.1 Conform to 1.1.1 and 1.2.1.

3.1.2 Conform to 1.2.1, 1.3.1 and 1.3.2.

**3.2 Clinically diagnosed cases**

3.2.1 Dengue fever

Consistent with the following one can be diagnosed as dengue fever:

3.2.1.1 Conform to 1.1.2,1.3.1 and 1.3.2 in suspected cases.

3.2.1.2 Conform to 3.1.2 and 1.3.4.

3.2.2 Dengue haemorrhagic fever

Conform to 3.2.1, 1.3.2, 1.3.3, and one of 1.2.3, 1.2.4, and 1.2.5.

3.2.3 Dengue shock syndrome

Conform to 3.2.2 and 1.2.6.

**3.3 Laboratory-confirmed cases**

Conform to 3.2, and conform to 1.3.5, 1.3.6 or 1.3.7.

Following the new classification of dengue cases proposed by the World Health Organization[2], the National Health Commission updated the diagnostic criteria for dengue fever in mainland China in 2018 based on the latest evidences in the epidemiology, diagnosis, treatment and laboratory testing of dengue fever. The updated standard (criteria number: WS 216-2018) are as follows[3].

**Dengue fever disease diagnostic criteria after 2018**

**1 Diagnostic basis**

**1.1 Epidemiology history**

The individuals had been to an epidemic area of dengue fever within 14 days before the onset of the disease, or there was (were) case(s) of dengue fever within 1 month around their place

of residence or workplace.

**1.2 Clinical manifestation**

1.2.1 Acute onset, sudden high fever, obvious fatigue, anorexia, nausea, etc., often accompanied by severe headache, orbital pain, systemic muscle pain, bone and joint pain and other symptoms, may be accompanied by facial, neck, chest flushing, conjunctival congestion and so on.

1.2.2 Rash: Congestive rash or punctate haemorrhagic rash appeared on the facial limbs from the $3^{rd}$ day to the $6^{th}$ day of the course of the disease. Typical rashes are needle-like bleeding points and “skin island"-like manifestations seen in the limbs. The rash is distributed in the trunk or head and face of the limbs, mostly itching, not desquamation. It lasts 3 to 5 days.

1.2.3 Bleeding tendency: Some patients may have different degrees of bleeding, such as subcutaneous bleeding, ecchymosis at the injection site, gingival bleeding, and nose.

1.2.4 Severe bleeding: subcutaneous hematoma, gross hematuria, gastrointestinal, thoracic and abdominal, vaginal, intracranial and other parts of the bleeding.

1.2.5 Severe organ injury: acute myocarditis, acute respiratory distress syndrome, acute liver injury, acute renal insufficiency, central nervous system.

1.2.6 Shock: tachycardia, cold extremities, prolonged capillary filling time > 3s, weak or undetectable pulse, decreased pulse pressure or undetectable blood pressure.

**1.3 Laboratory examination**

1.3.1 Leukopenia and/or thrombocytopenia.

1.3.2 Dengue virus IgM antibody was positive.

1.3.3 Dengue virus NS1 antigen test was positive within 5 days of onset.

1.3.4 The titer of serum specific IgG antibody in the convalescent stage of dengue virus was 4 times or more higher than that in the acute phase.

1.3.5 Dengue virus was isolated from blood, cerebrospinal fluid or tissues of patients in acute phase.

1.3.6 Dengue virus nucleic acid was detected by RT-PCR or real-time fluorescence

quantitative RT-PCR.

## 2 Diagnosis principle

The comprehensive judgment was made according to the epidemiological evidence, clinical manifestations and laboratory examination results of the patients.

## 3 Diagnosis

3.1 Probable cases

Consistent with the following one can be diagnosed as probable cases:

a) Conform to 1.1 and 1.2.1.

b) Conform to 1.2.1 and 1.3.1.

3.2 Clinically diagnosed cases

Consistent with the following one can be diagnosed as clinically diagnosed cases:

a) Conform to 3.1a) and 1.3.1, and conform to 1.2.2 or 1.2.3.

b) Conform to 3.1, and conform to 1.3.2 or 1.3.3.

3.3 Laboratory-confirmed cases

Conform to 3.1 or 3.2, and conform to 1.3.4, 1.3.5 or 1.3.6.

**Additional information regarding the estimation of seasonal parameters**

To describe the seasonal pattern in the weekly time series of dengue infections and estimate its peak timing and amplitude of the annual and semi-annual periodicities in each provincial administration, we used a generalized linear model (GLM) with a Gaussian distribution[4–6]:

$$Y_i(t) = \beta_0 + \beta_1 \sin(2\pi t/53) + \beta_2 \cos(2\pi t/53) + \beta_3 \sin(4\pi t/53) + \beta_4 \cos(4\pi t/53) + e(t)$$

where $Y_i(t)$ are the weekly standardized counts of dengue cases isolates in provincial administration $i$, where standardization is obtained by dividing weekly values by the annual number of dengue cases, $t$ is a running index for week, and $\beta_0$, $\beta_1$, $\beta_2$, $\beta_3$ and $\beta_4$ are the intercept term and the seasonal pattern term to be estimated, respectively.

Specifically, equations for the seasonal parameters of interest are as follows:

The phase angle is estimated as $\hat{\psi} = -\arctan(\beta_1/\beta_2)$

The average annual peak timing is estimated as $Peak = 53 * (1 - \psi/\pi)/2$

The amplitude of the annual periodicity is estimated as $Ampann = \sqrt{\beta_1^2 + \beta_2^2}$ ,

The amplitude of the semi-annual periodicity is estimated as $Ampsemiann = \sqrt{\beta_3^2 + \beta_4^2}$

To compare the difference in dengue fever outbreak levels among provincial administration, we divided $Ampann$ and $Ampsemiann$ by the mean of dengue time series to compare the relative amplitude for annual and semi-annual periodicity. To evaluate the relative difference of annual and semi-annual dengue fever periodicity by province, we calculated the ratio among the amplitude for the semi-annual periodicity and the sum of the amplitudes for annual and semi-annual periodicity. The ratio approaching the number of 1 indicates the predominant of semi-annual periodicity, whereas the ratio approaching the number of 0 indicates the predominant of annual periodicity.

## Dengue fever transmission model

In this study, we referred to previous studies to extend the above isolated model to a metapopulation model to simulate the transmission of dengue infections in mainland China at city level[7,8]. In this extended model, we investigated two types of population movements, which are regular daily commuting and diffusive random movements. We included 337 cities in the model, comprising 4 municipalities, and 333 prefecture-level administrative regions (293 prefecture-level cities, 7 prefectures, 30 autonomous prefectures and 3 leagues). We divided the regular daily commuting into two parts: 1) during the daytime, commuters go to the working region and mix with the population in working region; 2) at the nighttime, commuters return from the working region and mix with the population in their hometown. In addition to regular commuting, a fraction of the population travels for purposes other than work. These regular commuting and random movement only occur between human populations, while mosquitoes generally don't move long distances. Therefore, this study assumed that the mosquitoes are fixed in a single city from birth to death. We also distinguished between reported infected individuals and unreported infected individuals, and defined different modes of transmission for them. We modelled the daytime and nighttime periods respectively, and use a random sampling of Poisson distribution to generate the dynamics of each compartment. The metapopulation model can be expressed as follows:

### 1 Daytime transmission:

### 1.1 Compartment of human transfer process:

$$S_{ij}^{H}(t+dt_1)=S_{ij}^{H}(t)-Pois\left(\frac{\lambda_i(t)\tau_i(t)\beta_i^{H}(t)S_{ij}^{H}(t)I_i^{M}(t)}{N_i^{Hd}(t)}dt_1\right)$$

$$+Pois\left(\theta dt_1\frac{N_{ij}^{H}-I_{ij}^{Hr}(t)}{N_i^{Hd}(t)}\sum_{k\neq i}\frac{\overline{N}_{ik}^{H}\sum_l S_{kl}^{H}(t)}{N_k^{Hd}(t)-\sum_l I_{lk}^{Hr}(t)}\right)$$

$$-Pois\left(\theta dt_1\frac{S_{ij}^{H}(t)}{N_i^{Hd}(t)-\sum_l I_{li}^{Hr}(t)}\sum_{k\neq i}\overline{N}_{ki}^{H}\right)$$

$$I_{ij}^{Hr}(t+dt_1)=I_{ij}^{Hr}(t)+Pois\left(\lambda_i(t)\alpha\frac{\tau_i(t)\beta_i^{H}(t)S_{ij}^{H}(t)I_i^{M}(t)}{N_i^{Hd}(t)}dt_1\right)-Pois\left(\frac{I_{ij}^{Hr}(t)}{D}dt_1\right)$$

$$I_{ij}^{Hu}(t+dt_1)=I_{ij}^{Hu}(t)+Pois\left(\lambda_i(t)(1-\alpha)\frac{\tau_i(t)\beta_i^{H}(t)S_{ij}^{H}(t)I_i^{M}(t)}{N_i^{Hd}(t)}dt_1\right)-Pois\left(\frac{I_{ij}^{Hu}(t)}{D}dt_1\right)$$

$$+Pois\left(\theta dt_1\frac{N_{ij}^{H}-I_{ij}^{Hr}(t)}{N_i^{Hd}(t)}\sum_{k\neq i}\frac{\overline{N}_{ik}^{H}\sum_l I_{kl}^{Hu}(t)}{N_k^{Hd}(t)-\sum_l I_{lk}^{Hr}(t)}\right)$$

$$-Pois\left(\theta dt_1\frac{I_{ij}^{Hu}(t)}{N_i^{Hd}(t)-\sum_l I_{li}^{Hr}(t)}\sum_{k\neq i}\overline{N}_{ki}^{H}\right)$$

**1.2 Compartment of mosquito transfer process:**

$$S_i^{M}(t+dt_1)=S_i^{M}(t)-Pois\left(\frac{\lambda_i(t)\tau_i(t)\beta_i^{M}(t)S_i^{M}(t)\sum_k\left(I_{ki}^{Hr}(t)+I_{ik}^{Hu}(t)\right)}{N_i^{Hd}(t)}dt_1\right)$$

$$+Pois\left(\mu_i^{Mb}(t)S_i^{M}(t)dt_1\right)+Pois\left(\mu_i^{Mb}(t)(1-U)I_i^{M}(t)dt_1\right)$$

$$-Pois\left(\mu^{Md}S_i^{M}(t)dt_1\right)$$

$$I_i^{M}(t+dt_1)=I_i^{M}(t)+Pois\left(\frac{\lambda_i(t)\tau_i(t)\beta_i^{M}(t)S_i^{M}(t)\sum_k\left(I_{ki}^{Hr}(t)+I_{ik}^{Hu}(t)\right)}{N_i^{Hd}(t)}dt_1\right)$$

$$+Pois\left(\mu_i^{Mb}(t)UI_i^{M}(t)dt_1\right)-Pois\left(\mu^{Md}I_i^{M}(t)dt_1\right)$$

$$N_i^{Hd}(t)=N_{ii}^{H}+\sum_{k\neq i}I_{ki}^{Hr}(t)+\sum_{k\neq i}\left(N_{ik}^{H}-I_{ik}^{Hr}(t)\right)$$

**2 Nighttime transmission:**

## 2.1 Compartment of human transfer process:

$$S_{ij}^{H}(t+1)=S_{ij}^{H}(t+dt_1)-Pois\left(\frac{\lambda_j(t)\tau_j(t)\beta_j^{H}(t)S_{ij}^{H}(t+dt_1)I_j^{M}(t+dt_1)}{N_j^{Hn}}dt_2\right)$$

$$+Pois\left(\theta dt_2\frac{N_{ij}^{H}}{N_j^{Hn}}\sum_{k\neq j}\frac{\overline{N}_{jk}^{H}\sum_{l}S_{lk}^{H}(t+dt_1)}{N_k^{Hn}-\sum_{l}I_{lk}^{Hr}(t+dt_1)}\right)$$

$$-Pois\left(\theta dt_2\frac{S_{ij}^{H}(t+dt_1)}{N_j^{Hn}-\sum_{k}I_{kj}^{Hr}(t+dt_1)}\sum_{k\neq i}\overline{N}_{kj}^{H}\right)$$

$$I_{ij}^{Hr}(t+1)=I_{ij}^{Hr}(t+dt_1)+Pois\left(\lambda_i(t)\alpha\frac{\tau_j(t)\beta_j^{H}(t)S_{ij}^{H}(t+dt_1)I_j^{M}(t+dt_1)}{N_j^{Hn}}dt2\right)$$

$$-Pois\left(\frac{I_{ij}^{Hr}(t+dt_1)}{D}dt_2\right)$$

$$I_{ij}^{Hu}(t+1)=I_{ij}^{Hu}(t+dt_1)+Pois\left(\lambda_i(t)(1-\alpha)\frac{\tau_j(t)\beta_j^{H}(t)S_{ij}^{H}(t+dt_1)I_j^{M}(t+dt_1)}{N_j^{Hn}}dt_2\right)$$

$$-Pois\left(\frac{I_{ij}^{Hu}(t+dt_1)}{D}dt_2\right)$$

$$+Pois\left(\theta dt_2\frac{N_{ij}^{H}}{N_j^{Hn}}\sum_{k\neq i}\frac{\overline{N}_{jk}^{H}\sum_{l}I_{lk}^{Hu}(t+dt_1)}{N_k^{Hn}-\sum_{l}I_{lk}^{Hr}(t+dt_1)}\right)$$

$$-Pois\left(\theta dt_2\frac{I_{ij}^{Hu}(t+dt_1)}{N_j^{Hn}-\sum_{k}I_{kj}^{Hr}(t+dt_1)}\sum_{k\neq i}\overline{N}_{kj}^{H}\right)$$

## 2.2 Compartment mosquito transfer process:

$$S_i^{M}(t+1)=S_i^{M}(t+dt_1)-Pois\left(\frac{\lambda_i(t)\tau_i(t)\beta_i^{M}(t)S_i^{M}(t+dt_1)\sum_{k}\left(I_{ki}^{Hr}(t+dt_1)+I_{ki}^{Hu}(t+dt_1)\right)}{N_i^{Hn}}dt_2\right)$$

$$+Pois\left(\mu_i^{Mb}(t)S_i^{M}(t+dt_1)dt_2\right)+Pois\left(\mu_i^{Mb}(1-U)I_i^{M}(t+dt_1)dt_2\right)$$

$$-Pois\left(\mu^{Md}S_i^{M}(t+dt_1)dt_2\right)$$

$$I_i^{M}(t+1)=I_i^{M}(t+dt_1)+Pois\left(\frac{\lambda_i(t)\tau_i(t)\beta_i^{M}(t)S_i^{M}(t+dt_1)\sum_{k}\left(I_{ki}^{Hr}(t+dt_1)+I_{ik}^{Hu}(t+dt_1)\right)}{N_i^{Hn}}dt_2\right)$$

$$+Pois\left(\mu_i^{Mb}(t)UI_i^{M}(t+dt_1)dt_2\right)-Pois\left(\mu^{Md}I_i^{M}(t+dt_1)dt_2\right)$$

$$N_i^{Hn}=\sum_{k}N_{ki}^{H}$$

Here, $S_{ij}^{H}$, $I_{ij}^{Hr}$, $I_{ij}^{Hu}$, and $N_{ij}^{H}$ are the susceptible, reported infected, unreported infected and total individuals in the commuting from city $j$ to city $i$ $(i \leftarrow j)$; $S_i^M$, $I_i^M$ are the susceptible and infected mosquitoes in city $i$; $Pois(\bullet)$ is a Poisson distribution with mean value $(\bullet)$; $\lambda_i(t)$ is the adjusted transmission rate between humans and mosquitoes in city $i$ on day $t$; $\tau_i(t)$ is the biting rate in city $i$ on day $t$; $\beta_i^M(t)$ is the probability for transmission of the infection from human to mosquito in city $i$ on day $t$; $\beta_i^H(t)$ is the probability for transmission of the infection from mosquito to human in city $i$ on day $t$; $\alpha$ is the fraction of reported infections; $D$ is the average duration of contagiousness for human; $\theta$ is a diffusive random mobility index, representing the relative rate of random mobility compared with regular commuting; $\mu_i^{Mb}(t)$ is the birth rate of mosquitoes in city $i$ on day $t$; $\mu^{Md}$ is the mortality rate of mosquitoes; $U$ is the vertical dissemination rate of infected mosquitoes and is constant over an outbreak; $N_i^{Hd}$, $N_i^{Hn}$ are the daytime and nighttime populations of city $i$; $\overline{N}_{ij}^{H} = (N_{ij}^{H} + N_{ji}^{H})/2$ is the average number of commuters between cities $i$ and $j$; $dt_1$, $dt_2$ are the transmission time during daytime and nighttime ($dt_1 = 1/3$ day and $dt_2 = 2/3$ day); We assume that if infected individuals are reported, these individuals will not continue to participate in population movements until these individual are cured. To explore the temporal and spatial changes for contact rate and reporting rate of dengue fever, we adopted the adjusted transmission rate $\lambda_i(t)$ to change in different cities and at different times.

**Model calibration**

We calibrated the transmission model based on the daily report of dengue fever cases, available at China CDC, which runs from the 140th day to the 364th day per year. To reduce the number of unknown parameters in this high-dimensional model, we determine the following parameters. Based on previous studies[9], we use a linear function to represent the relationship between ambient temperature $T$ and biting rate $\tau(t)$:

$$\tau(t) = 0.5(0.0043T(t) + 0.0943) \qquad 21℃ \leqslant T(t) \leqslant 32℃$$

Where $T(t)$ is the average ambient temperature (degree centigrade) on day $t$, and $\tau(t)$ is the biting rate.

In addition, the probability for transmission of the infection from human to mosquito $\beta_M$ is related to temperature $T$[10]:

$$\beta_M(t) = \begin{cases} -0.9037 + 0.0729T(t), & 12.4℃ \leqslant T(t) \leqslant 26.1℃ \\ 1, & 26.1℃ < T(t) < 32.5℃ \end{cases}$$

Similarly, the probability for transmission of the infection from mosquito to human $\beta_H$ is expressed as follow[10]:

$$\beta_H(t) = 0.001044T(t)(T(t) - 12.286)\sqrt{32.461 - T(t)}, \ 12.286℃ \leqslant T(t) \leqslant 32.461℃$$

We improved on Chen's study[11] to estimate the time-varying birth rate of the mosquito population $\mu_i^{Mb}(t)$ in each location from the measured mosquito larvae data BI. However, the BI is not measured in each location. First, we calculate the provincial average of the monthly BI data collected by each location. Then, the provincial BI is assigned to each

location in the province. The BI has little difference between years, and has a similar peak time and periodicity in each location. Therefore, we reduce the noise by smoothing the data with a sine function, and fit the monthly BI data to each day:

$$f(t) = \alpha \sin(2\pi t/365) + \beta \cos(2\pi t/365) + \max(BI)/2$$

Note that the intercept term of the fitting curve was set to half of the maximum measured value to avoid negative BI. The BI in Beijing is not investigated. Hence, we adopted the average data of neighboring provincial administration, which are Tianjin and Hebei. Similarly, we adopted the average BI data of neighboring provincial administration for Guizhou, which included Sichuan, Yunnan, Guangxi, Hunan, and Chongqing. For other provincial administration where BI isn't sampled (Gansu, Inner Mongolia, Ningxia, Qinghai, Xinjiang, Tibet, Heilongjiang, Jilin), we assumed that the number of mosquito vectors in these provinces remain constant.

Assuming that the number of mosquito populations remains unchanged after a one-year cycle, the standardized natural growth proportion sequence ($BI^a$) can be obtained by BI/BI*, where BI* is the inflection point of BI in which the first derivative is the maximum. The average oviposition period of mosquitoes was 16 days[11], therefore the time-varying natural birth rate of the mosquito population can be calculated by $\sqrt[16]{BI^a} - 1$. Finally, the birth rate of mosquitoes $\mu_i^{Mb}(t)$ can be calculated as the natural birth rate ($\sqrt[16]{BI^a} - 1$) plus the mortality rate of mosquitoes $\mu^{Md}$.

Based on our previous studies, we identified the following disease-related parameters ($D$, $\alpha$), mosquito-related parameters ($U$, $\mu^{Md}$) and mobility index ($\theta$). Specifically, these parameters are drawn from the posterior distribution using Latin hypercube sampling: $D \in [5, 7]$, $\alpha \in [0.25, 0.35]$, $U \in [0.25, 0.25]$, $\mu^{Md} \in [1/15, 1/15]$, $\theta \in [0.25, 0.35]$. Using similar posterior distributions for these five parameters, as well as similar overall results (see,

SI Part 3 Section 5, Sensitivity Analysis).

To initialize the transmission model, we seed reported infected individuals, unreported infected individuals and infected mosquitoes in locations with at least five confirmed cases. They represent imported cases, infections caused by unreported cases, and hatching of virus-carrying mosquitoes, respectively. According to the transmission pattern of dengue fever, we randomly draw reported infected individuals $I^{Hr}$, unreported infected individuals $I^{Hu}$ and infected mosquitoes $I^{M}$ from uniform distributions $[0,R]$, $[0,R]$ and $[0,12R]$ in seven days before the date $T_0$. The date $T_0$ is the day that the number of reported cases first exceeded 5; $R$ is the total number of reported cases between day $T_0$ and day $T_0+4$. Such setting satisfies the inconsistency of the time and scale of dengue fever occurring in different locations.

We set the initial susceptible population $S_{ij}^{H}(0)=N_{ij}^{H}-I_{ij}^{Hr}(0)-I_{ij}^{Hu}(0)$. Previous studies assumed that the number of mosquito populations is equal to the number of human populations $S_{i}^{M}(0)=S_{ii}^{H}(0)$ in location $i$. In our study, we added a weight parameter to this setting: $S_{i}^{M}(0)=\psi*S_{ii}^{H}(0)$, where $\psi$ denotes the average mosquito density in April and May (model runs from the 140$^{th}$ days per year) in the province where location $i$ belongs. Yunnan Province is a high mosquito density province, but the mosquito density data showed an unreasonable low value (recorded minimum mosquito density). Therefore, to reduce error, we adjusted the data of mosquito density in Yunnan Province by taking the average mosquito density in the provinces with approximate latitude and climate (Guangdong and Guangxi).

To get the burden and characteristics data of dengue fever in each city, we first estimate the adjusted transmission rate $\lambda_i(t)$ between humans and mosquitoes in city $i$ on day $t$. The

range of adjusted transmission rate $\lambda_i(t)$ is $[0.3, 0.5]$. The range of baseline adjusted transmission rate at the 140$^{th}$ day $\lambda_i(0)$ is sampled from range $[0.3, 0.38]$ using Latin hypercube sampling.

A data assimilation method, the ensemble adjusted Kalman filter (EAKF)[12], is used to estimate the parameters of the model. The EAKF is suitable for high-dimensional ensemble transmission models and has been successfully applied to infer the epidemiological parameters of infectious diseases[7,8,11,13,14]. The EAKF takes all parameters and variables as a state space of the system. Moreover, the EAKF assumes that both the prior and posterior are Gaussian distributions, and adjusts the prior distribution to the posterior distribution using the Bayesian rule: $posterior \propto prior \times likelihood$.

For the observed variables (i.e., daily incidence), the ensemble members were updated deterministically, and retain the higher moments of the prior distribution in the posterior distribution. Unobserved variables and parameters were updated according to their covariation with observed variables and can be calculated indirectly from the set. EAKF also adjusts the unobserved state variables and parameters based on their covariation with the observed state variables. Further details on the EAKF scheme can be found in the study of Anderson[12].

The EAKF algorithm uses the posterior estimate from the previous time step as the prior for current time step, and updates the model parameters in turn at each time step. Therefore, the same parameter is autocorrelation at continuous time points, which limits the volatility of parameter estimation. The sensitivity analysis showed that the inference results are robust to the selection of the initial prior distribution (see, SI Part 3 Section 5, Sensitivity analysis).

To further test the identifiability of the parameters, we applied a transmission model to

generate simulated dengue outbreak data for all cities. In this transmission model, we fix the adjusted transmission rate $\lambda_i(t) = 0.4$. The simulated cases were generated from the 140th day to the 364th day, consistent with the main analysis.

We adopted the model-generated outbreaks to infer and compare the estimated parameters and “real” parameters for the five major regions of dengue outbreak. When inferring the transmission rate parameters, we set three different initial adjusted transmission rate parameters: 1) The initial adjusted transmission rate $\lambda_i(0) \in [0.3, 0.49]$ includes “real” parameters; 2) The initial adjusted transmission rate $\lambda_i(0) \in [0.3, 0.39]$ is less than “real” parameters; 3) The initial adjusted transmission rate $\lambda_i(0) \in [0.41, 0.5]$ is more than “real” parameters. Even if the initial parameter setting is not included in the “real” parameters, the inference system can generally capture the time variation of the parameters in the five major cities (Figure S22). This synthetic test showed that the model inference system can reasonably recover the key time-varying parameters in major cities.

**Urban agglomerations**

In this study, we revealed the dengue transmission dynamics of four major urban agglomerations, which are Yunnan Guangxi border area, Pearl River Delta, Fujian Zhejiang coast area, and Chongqing. The transmission characteristics of dengue fever in these urban agglomerations were aggregated from city-level estimates. In this study, the cities included in the urban agglomerations analyzed are as follows:

1) Yunnan Guangxi border area: Nujiang Lisu Autonomous Prefecture YN, Baoshan city YN, Dehong Dai and Jingpo Autonomous Prefecture YN, Lincang city YN, Pu'er city YN, Xishuangbanna Dai Autonomous Prefecture YN, Honghe Hani and Yi Autonomous Prefecture YN, Wenshan Zhuang and Miao Autonomous Prefecture YN, Baise city GX, Chongzuo city GX, Fangchenggang city GX.

2) Pearl River Delta: Guangzhou city GD, Foshan city GD, Zhaoqing city GD, Shenzhen city

GD, Dongguan city GD, Huizhou city GD, Zhuhai city GD, Zhongshan city GD, Jiangmen city GD.

3) Fujian Zhejiang coast area: Zhangzhou city FJ, Xiamen city FJ, Quanzhou City FJ, Putian city FJ, Fuzhou city FJ, Ningde city FJ, Wenzhou city ZJ, Taizhou city ZJ, Ningbo city ZJ, Zhoushan city ZJ.

4) Chongqing: Chongqing city CQ.

**Sensitivity analysis**

To test the robustness of the parameter estimation results, a number of sensitivity analyses were carried out. In each sensitivity analysis, we only change one parameter:

1) Sensitivity to fixed parameters. First, a longer infection period is considered $D \in [7, 9]$ (Figure S23). Secondly, the reporting rate is increased $\alpha \in [0.35, 0.45]$ (Figure S24) and decreased $\alpha \in [0.15, 0.25]$ (Figure S25) in the model respectively. Finally, the different random movement of population between cities is considered (Figure S26).

2) Sensitivity to initial parameters. We reduced the initial adjusted transmission rate parameter range $\lambda_i(0) \in [0.3, 0.34]$ to see whether a smaller parameter range can correctly fit the infectious disease outbreak curve (Figure S27).

**Supplementary Figure 1. Map of the 31 provincial administrations in mainland China included in this analysis (China has a total of 34 provincial administration).** The different colours represent the different climatic or geographic regions. (a) Climate regions in Mainland China. Blue: mid-temperate region; green: warm-temperate region; black: cold-temperate region; yellow: sub-tropic region; red: tropic region. (b) Included geographic regions in Mainland China. Northeast: Liaoning, Jilin, Heilongjiang; North: Beijing, Tianjin, Hebei, Shanxi, Inner Mongolia; Northwest: Shaanxi, Gansu, Qinghai, Ningxia, Xinjiang; East: Shanghai, Jiangsu, Zhejiang, Anhui, Fujian, Jiangxi, Shandong; Central: Henan, Hubei, Hunan; Southwest: Chongqing, Sichuan, Guizhou, Yunnan, Tibet; South: Guangdong, Guangxi, Hainan.

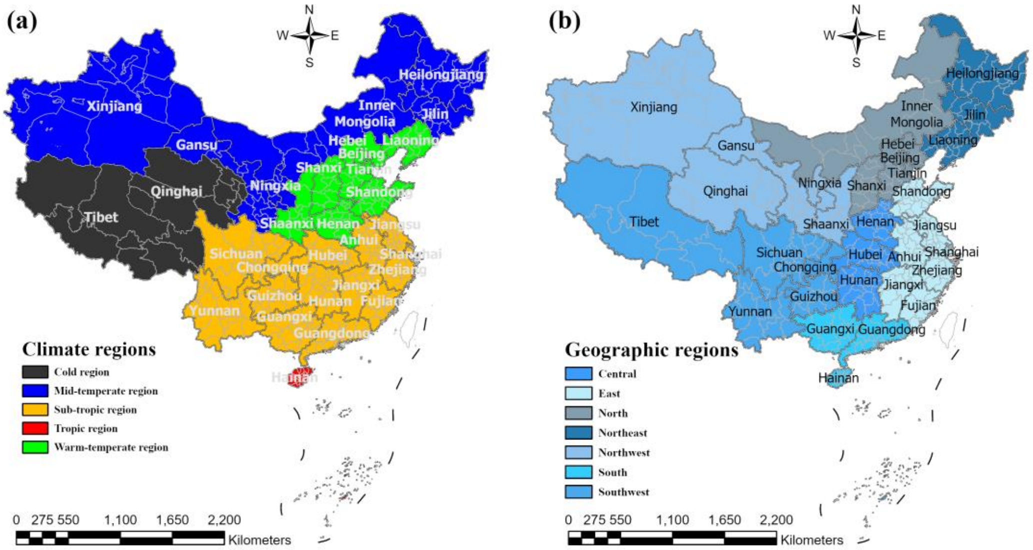

**Supplementary Figure 2. Spatio-temporal distribution for local cases of dengue fever in mainland China from 2013 to 2020.** The colours from yellow to red indicates the number of cases from less to more, and the number of cases in specific places is shown in the legend. Gray represents that there were no local cases of dengue fever reported in the region during the year.

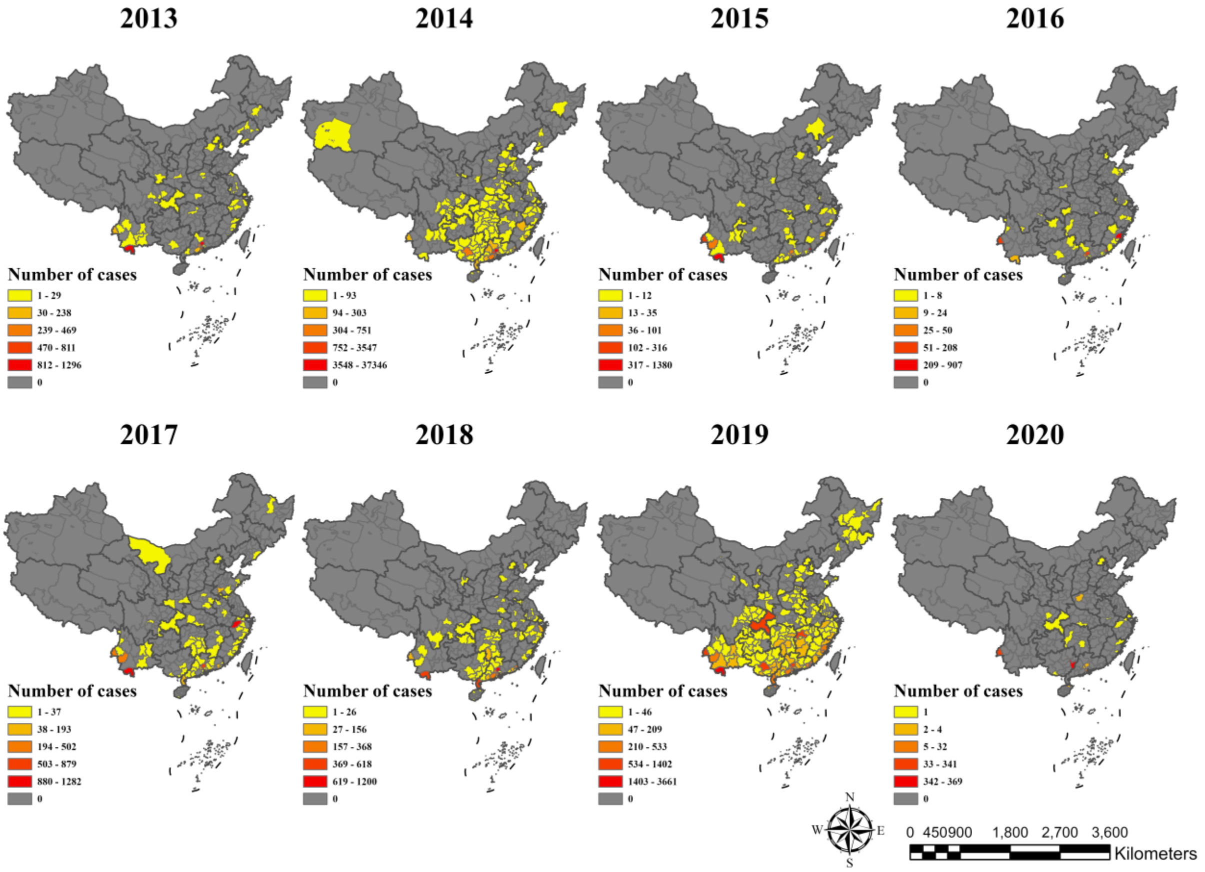

**Supplementary Figure 3. Spatio-temporal distribution for imported cases of dengue fever in mainland China from 2013 to 2020.** The colour from yellow to red indicates the number of cases from less to more, and the number of cases in specific places is shown in the legend. Gray represents that there were no imported cases of dengue fever reported in the region during the year.

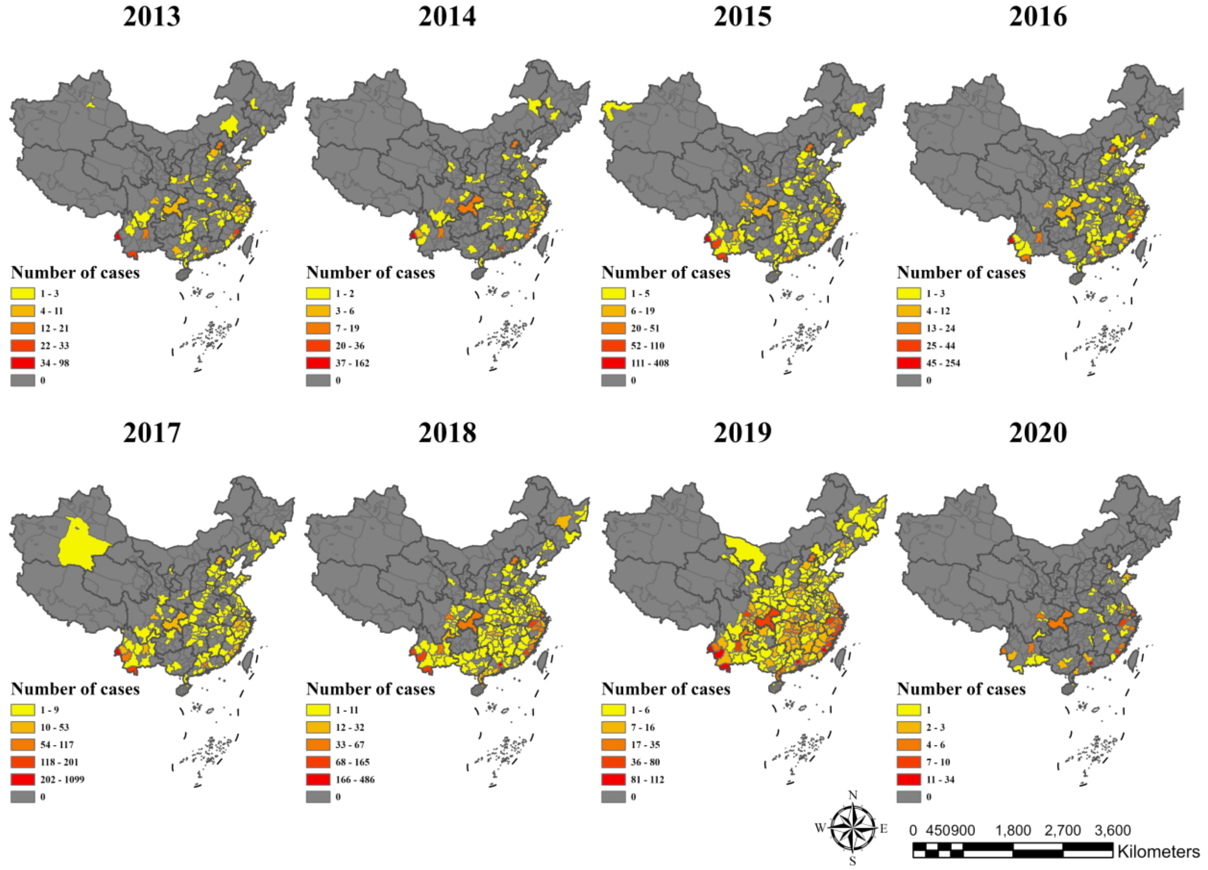

**Supplementary Figure 4. The source-sink relationship of dengue fever in mainland China from 2013 to 2020.** The arrow direction represents the source-sink relationship direction. Among the source countries, “Others” represents the source countries with less than 1% imported cases, and some unspecified source countries. “Low-imported” represents the provinces with low imported cases (less than 1%).

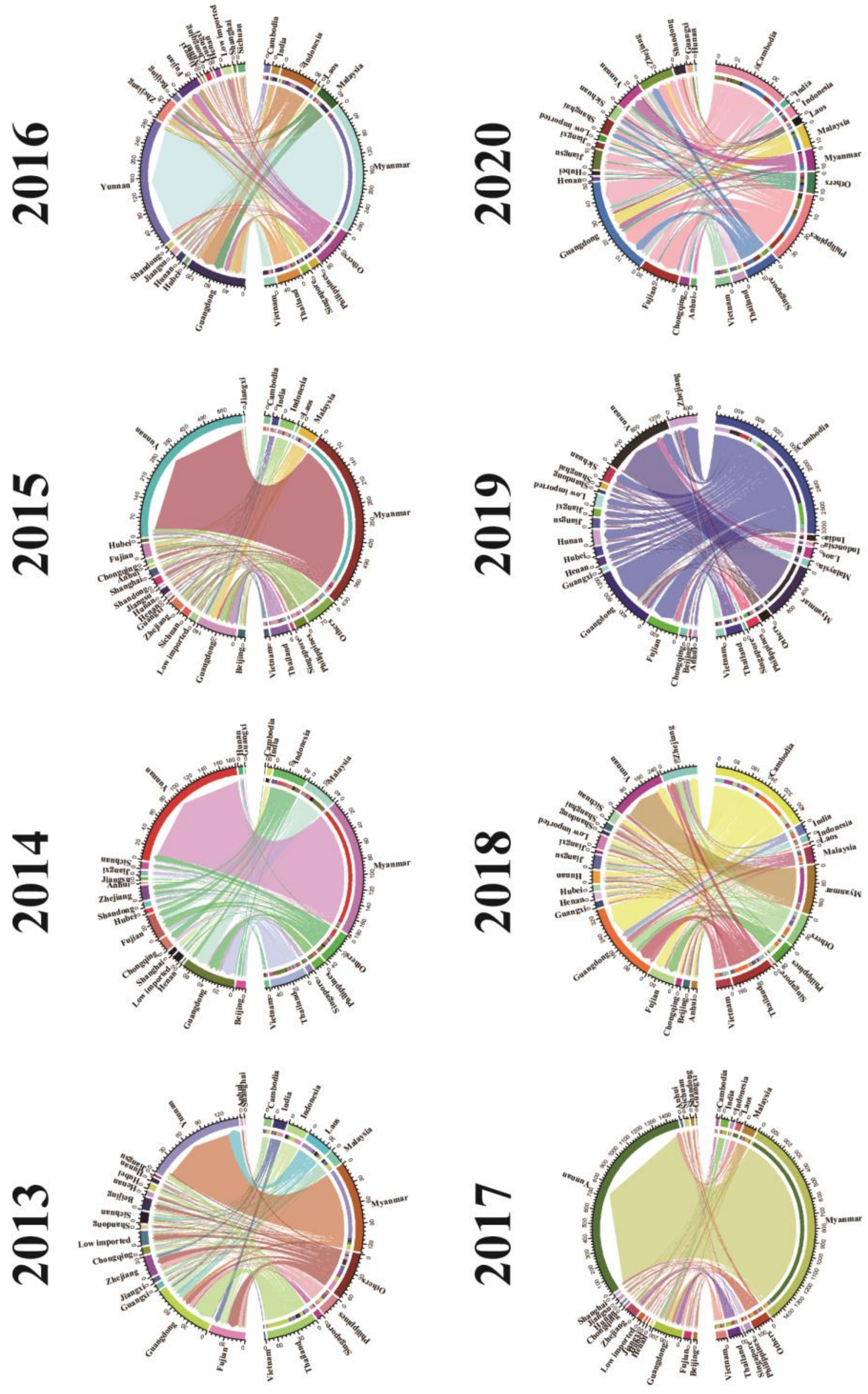

**Supplementary Figure 5. The source-sink relationship of dengue fever in province with low imported cases (less than 1%).** The arrow direction represents the source-sink relationship direction. Among the source countries, “Others” represents the source countries with less than 1% imported cases, and some unspecified source countries.

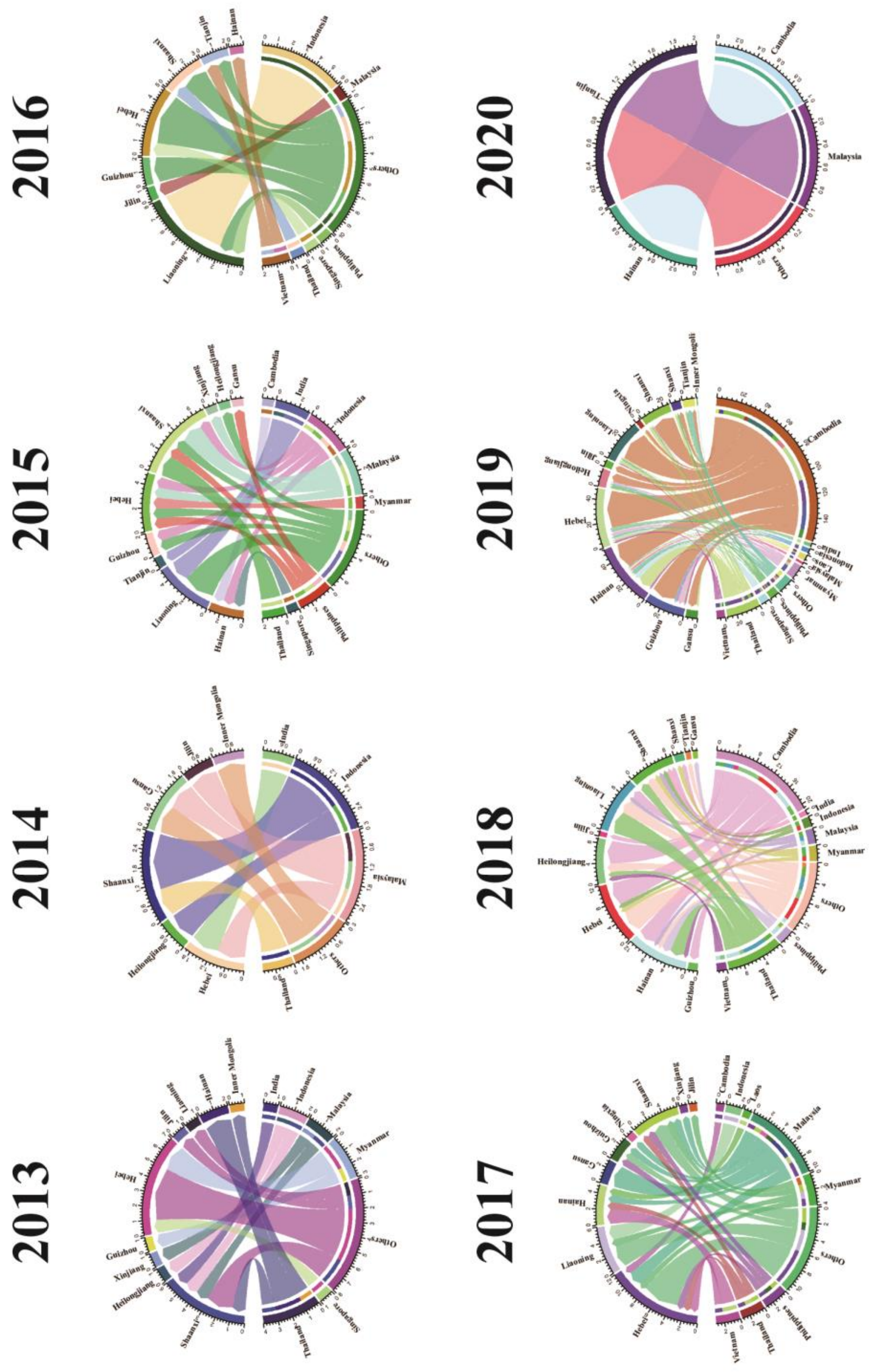

**Supplementary Figure 6. Nightingale rose chart for age distribution of local dengue cases in provinces of mainland China from 2013 to 2020.** Each "petal" represents different provinces, and the proportion of each age group in the province is distinguished by different colours.

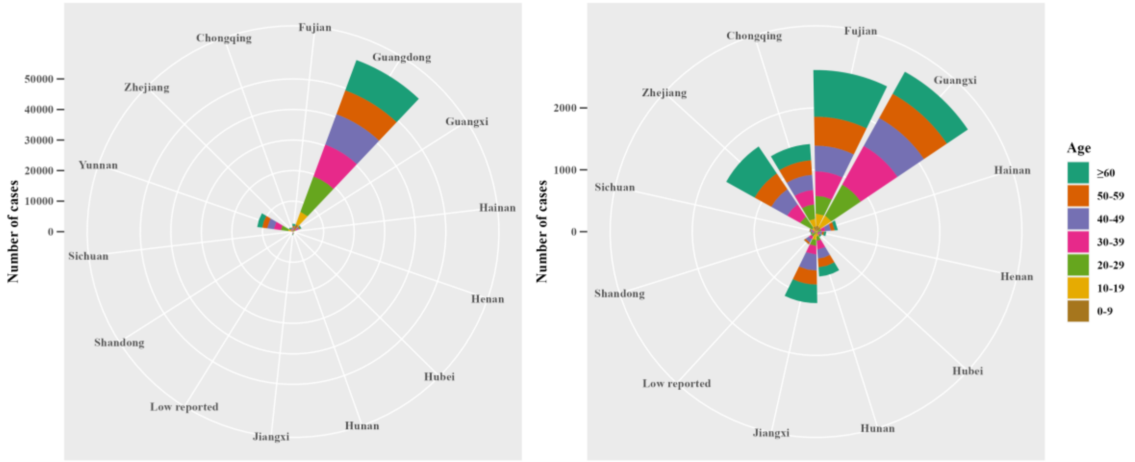

**Supplementary Figure 7. Onset-to-diagnosis distributions of dengue fever cases with different onset time and different ages.**

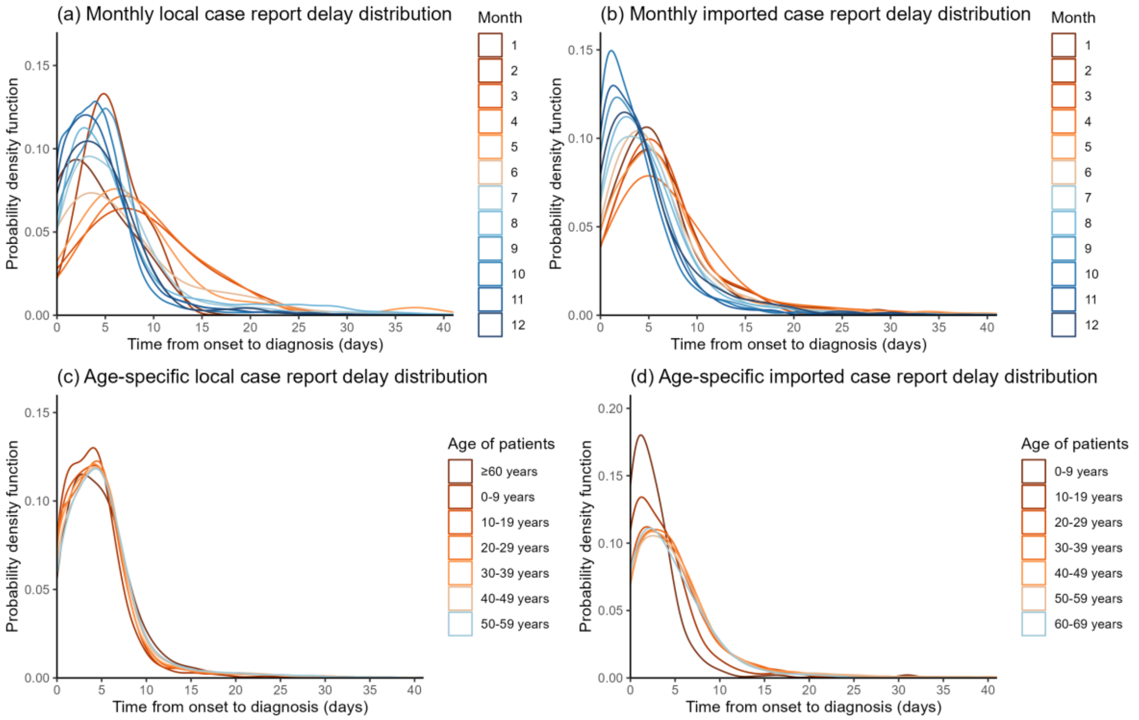

**Supplementary Figure 8. The geographical location of meteorological monitoring stations (during 2013-2020) and mosquito vector data survey sites (during 2013-2020) in China.**

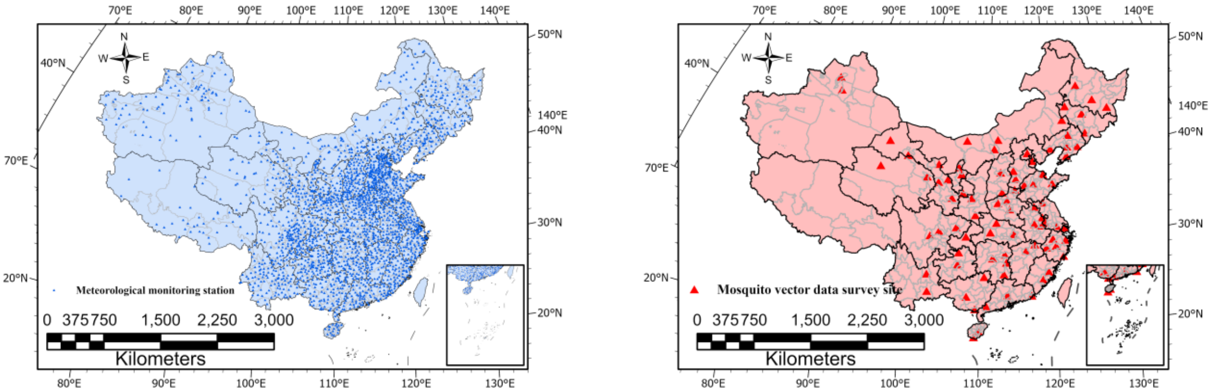


Notes: a: meteorological monitoring stations (N=2441);

b: mosquito vector data survey sites (N=89).

**Supplementary Figure 9. Distribution of mosquito vectors in each provincial administration.** The number of Aedes aegypti and Aedes albopictus is the average number of mosquitoes captured per light trap in the outbreak season (July, August, September) in the sample survey area of each provincial administration.

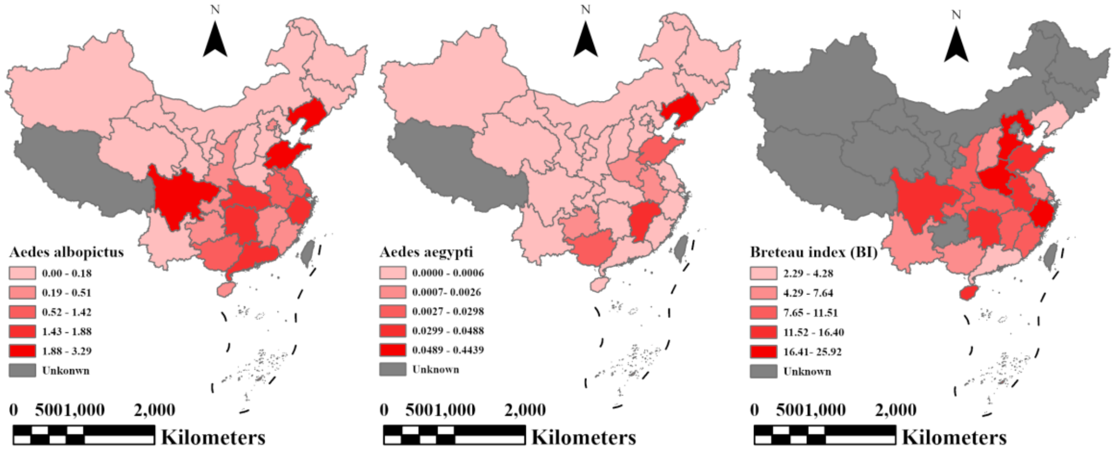

**Supplementary Figure 10. The average annual ambient temperature (°C) in mainland China from 2013 to 2019.**

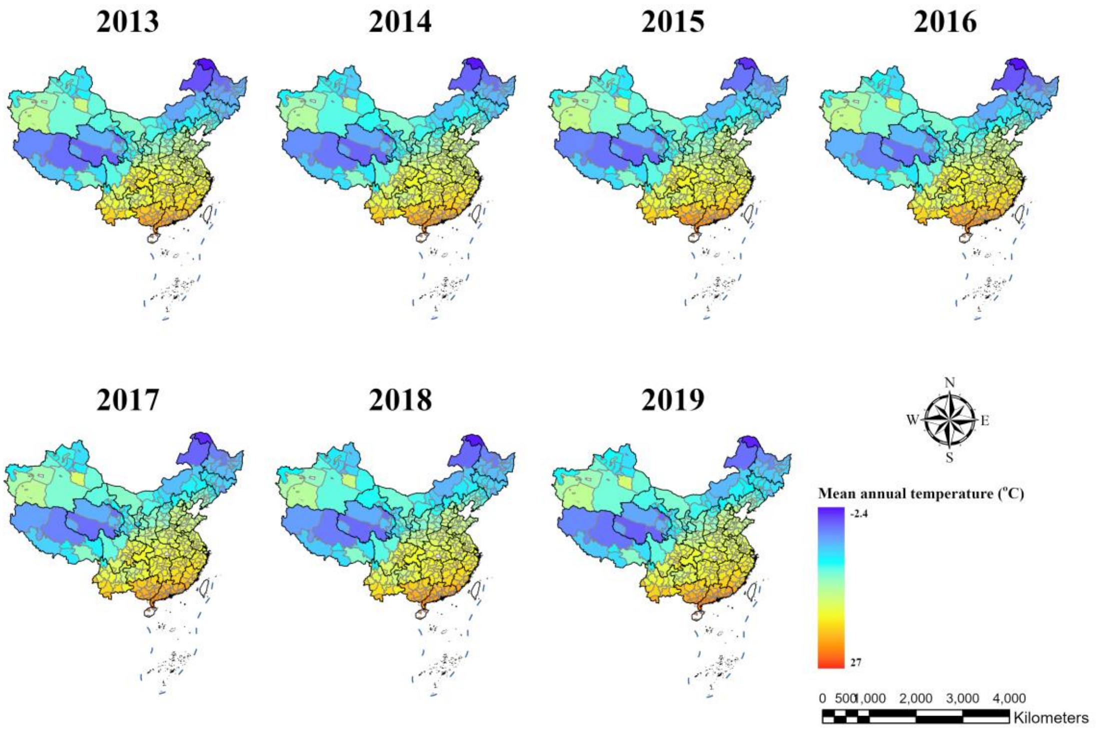

**Supplementary Figure 11. The fit of province-specific seasonal models for local dengue case in Beijing, Tianjin, Hebei, Shanxi, Inner Mongolia, Liaoning, Jilin, Heilongjiang and Shanghai.** Black curve represents observed weekly cases standardized by the annual cases; red curve represents the fitted seasonal model based on linear regression with harmonic terms for annual and semi-annual periodicities; amp1 is the annual amplitude and amp2 is the semiannual amplitude.

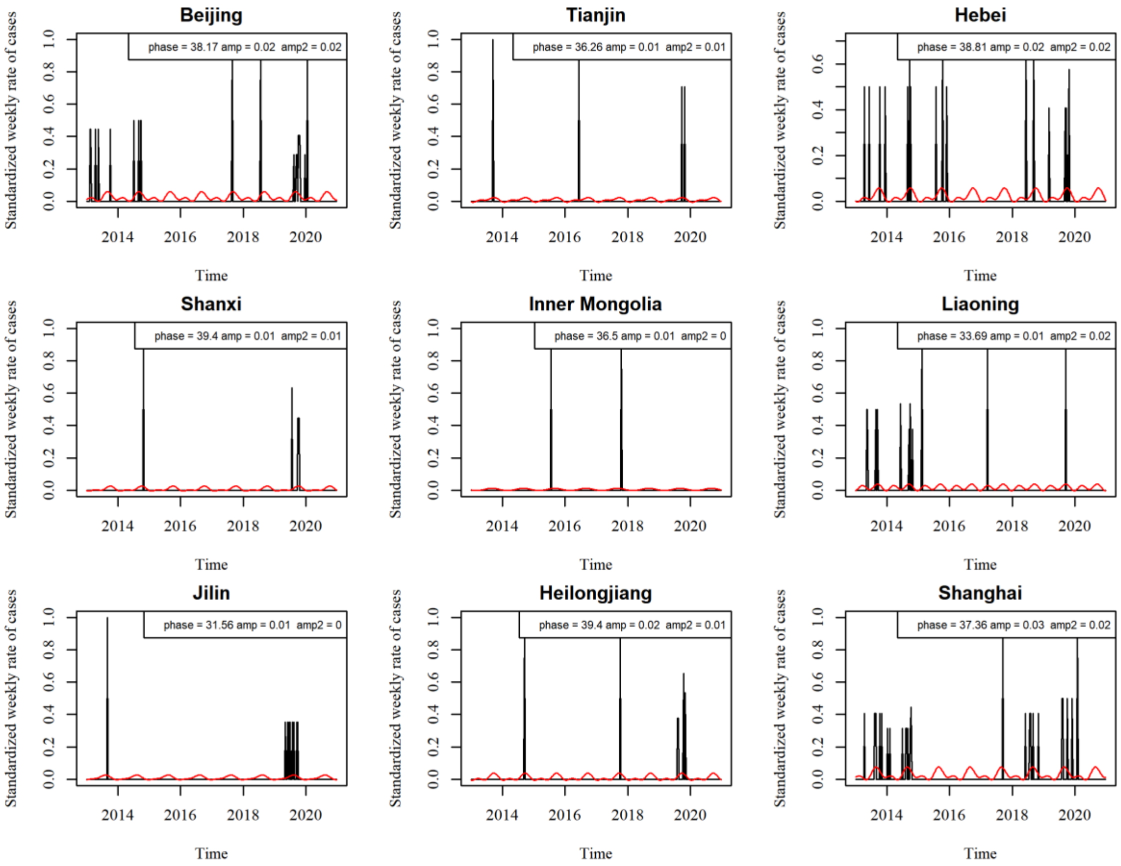

**Supplementary Figure 12. The fit of province-specific seasonal models for local dengue case in Jiangsu, Zhejiang, Anhui, Fujian, Jiangxi, Shandong, Henan, Hubei and Hunan.** Black curve represents observed weekly cases standardized by the annual cases; red curve represents the fitted seasonal model based on linear regression with harmonic terms for annual and semi-annual periodicities; amp1 is the annual amplitude and amp2 is the semiannual amplitude.

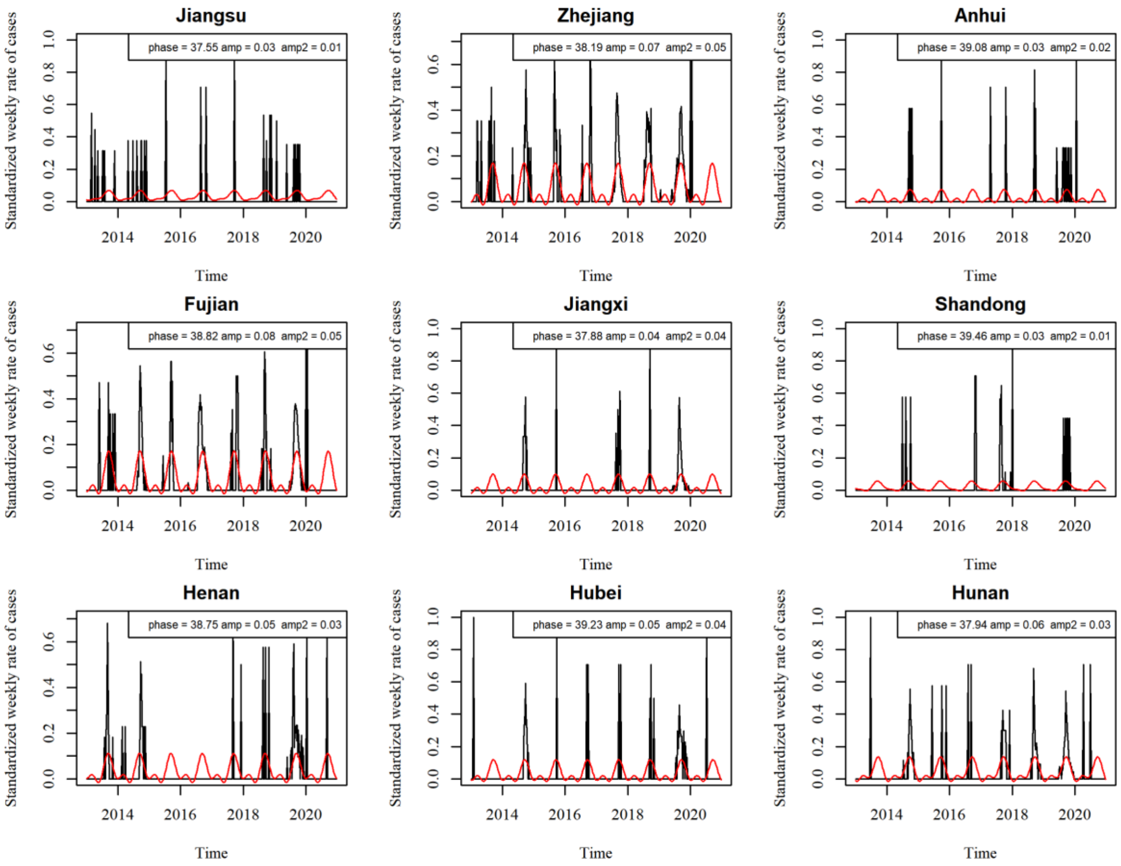

**Supplementary Figure 13. The fit of province-specific seasonal models for local dengue case in Guangdong, Guangxi, Hainan, Chongqing, Sichuan, Guizhou, Yunnan, Tibet and Shaanxi.** Black curve represents observed weekly cases standardized by the annual cases; red curve represents the fitted seasonal model based on linear regression with harmonic terms for annual and semi-annual periodicities; amp1 is the annual amplitude and amp2 is the semiannual amplitude.

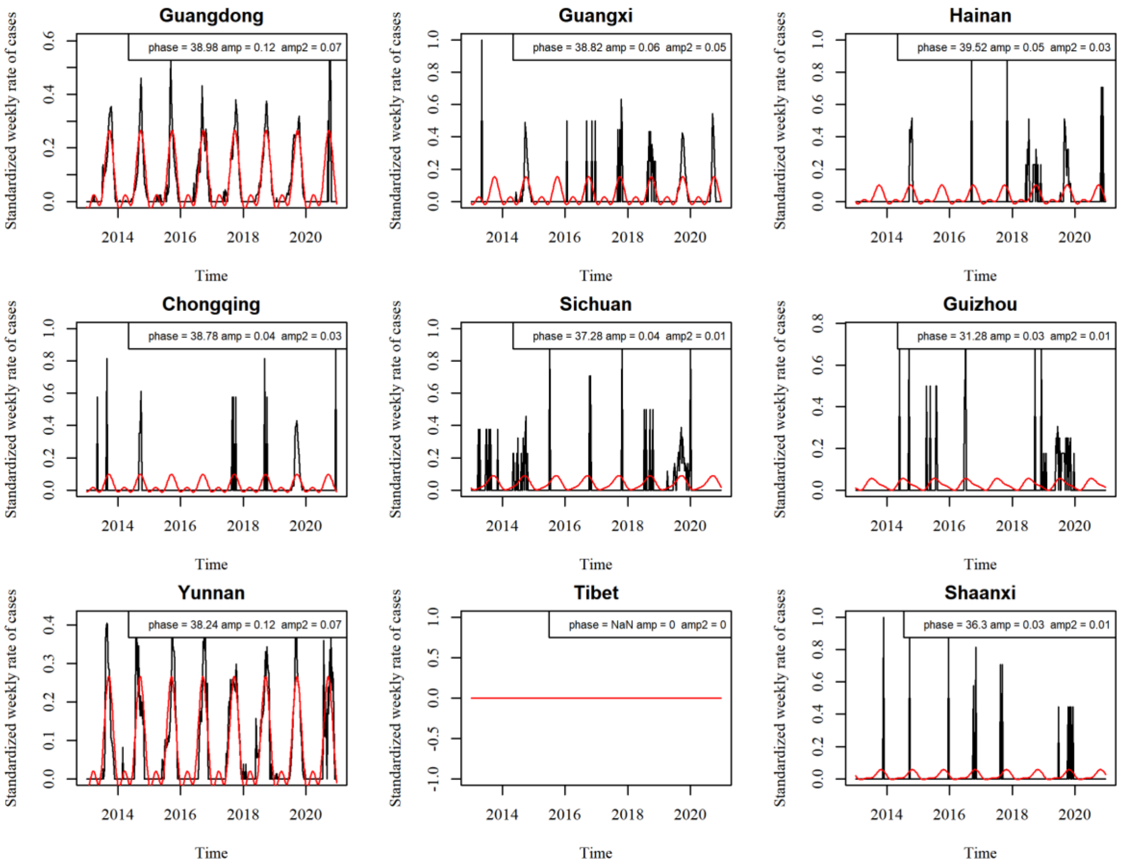

**Supplementary Figure 14. The fit of province-specific seasonal models for local dengue case in Gansu, Qinghai, Ningxia and Xinjiang.** Black curve represents observed weekly cases standardized by the annual cases; red curve represents the fitted seasonal model based on linear regression with harmonic terms for annual and semi-annual periodicities; amp1 is the annual amplitude and amp2 is the semiannual amplitude.

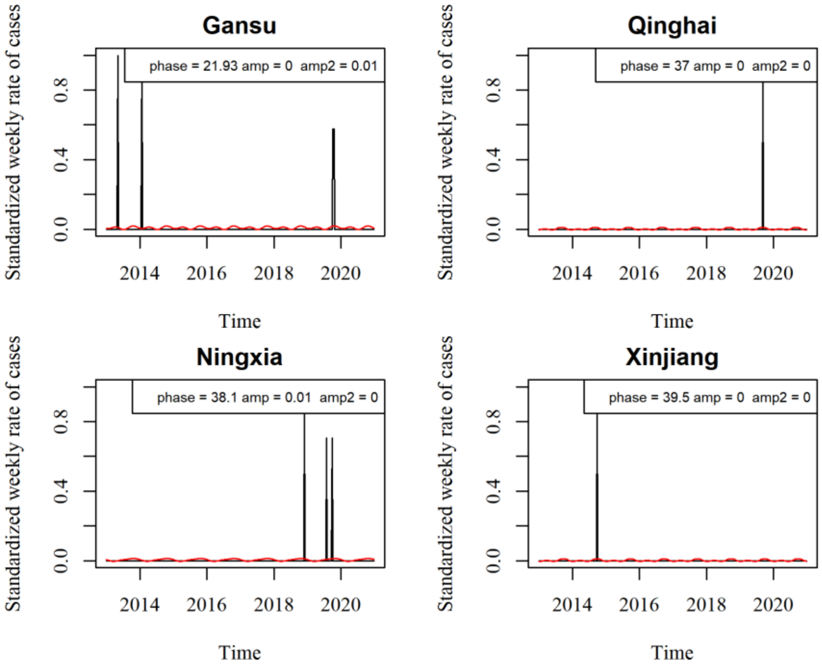

**Supplementary Figure 15. The fit of province-specific models for imported dengue case in Beijing, Tianjin, Hebei, Shanxi, Inner Mongolia, Liaoning, Jilin, Heilongjiang and Shanghai.** Black curve represents observed weekly cases standardized by the annual cases; red curve represents the fitted seasonal model based on linear regression with harmonic terms for annual and semi-annual periodicities; amp1 is the annual amplitude and amp2 is the semiannual amplitude.

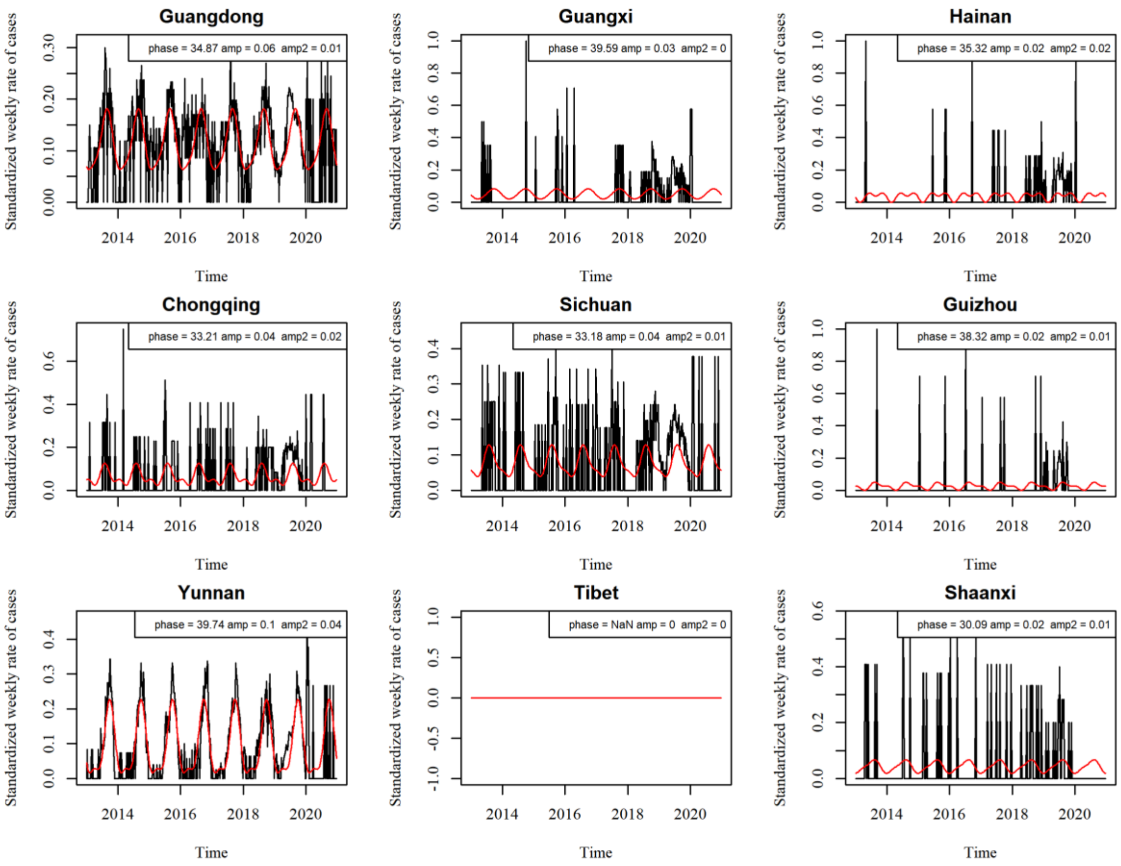

**Supplementary Figure 16. The fit of province-specific seasonal models for imported dengue case in Jiangsu, Zhejiang, Anhui, Fujian, Jiangxi, Shandong, Henan, Hubei and Hunan.** Black curve represents observed weekly cases standardized by the annual cases; red curve represents the fitted seasonal model based on linear regression with harmonic terms for annual and semi-annual periodicities; amp1 is the annual amplitude and amp2 is the semiannual amplitude.

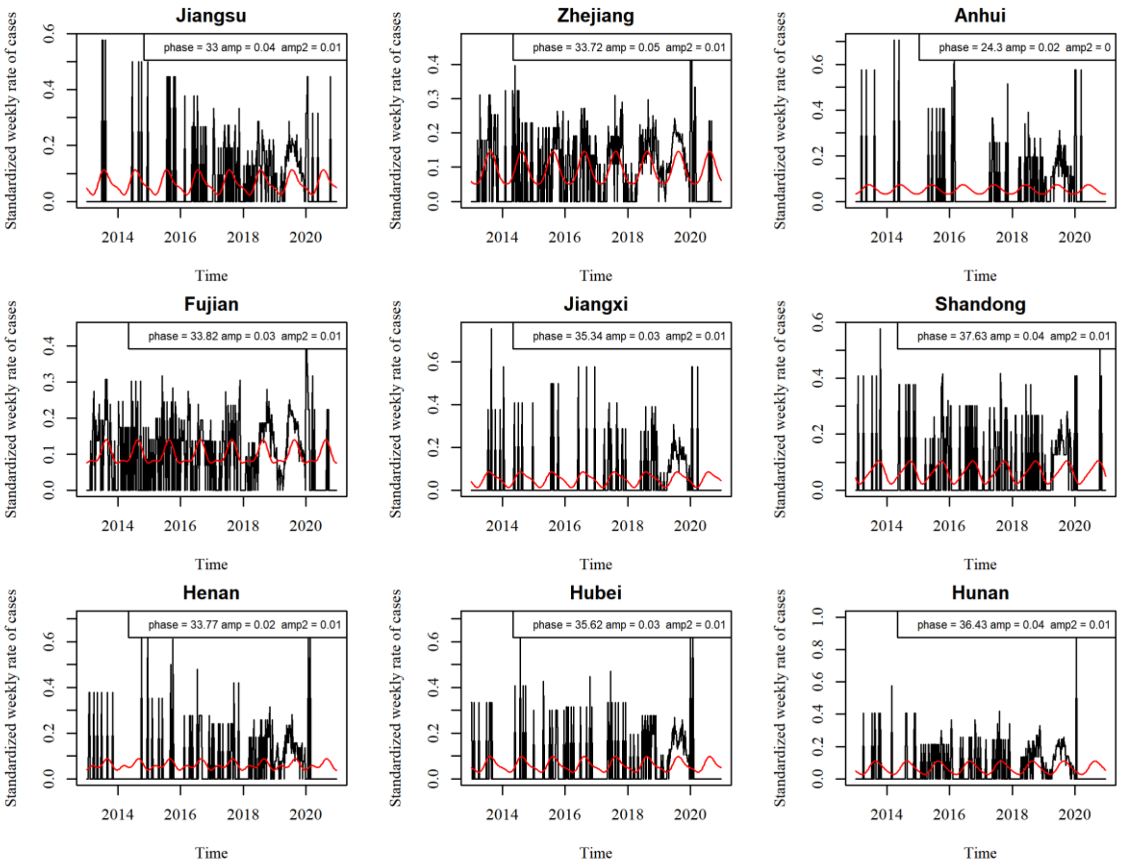

**Supplementary Figure 17. The fit of province-specific seasonal models for imported dengue case in Guangdong, Guangxi, Hainan, Chongqing, Sichuan, Guizhou, Yunnan, Tibet and Shaanxi.** Black curve represents observed weekly cases standardized by the annual cases; red curve represents the fitted seasonal model based on linear regression with harmonic terms for annual and semi-annual periodicities; amp1 is the annual amplitude and amp2 is the semiannual amplitude.

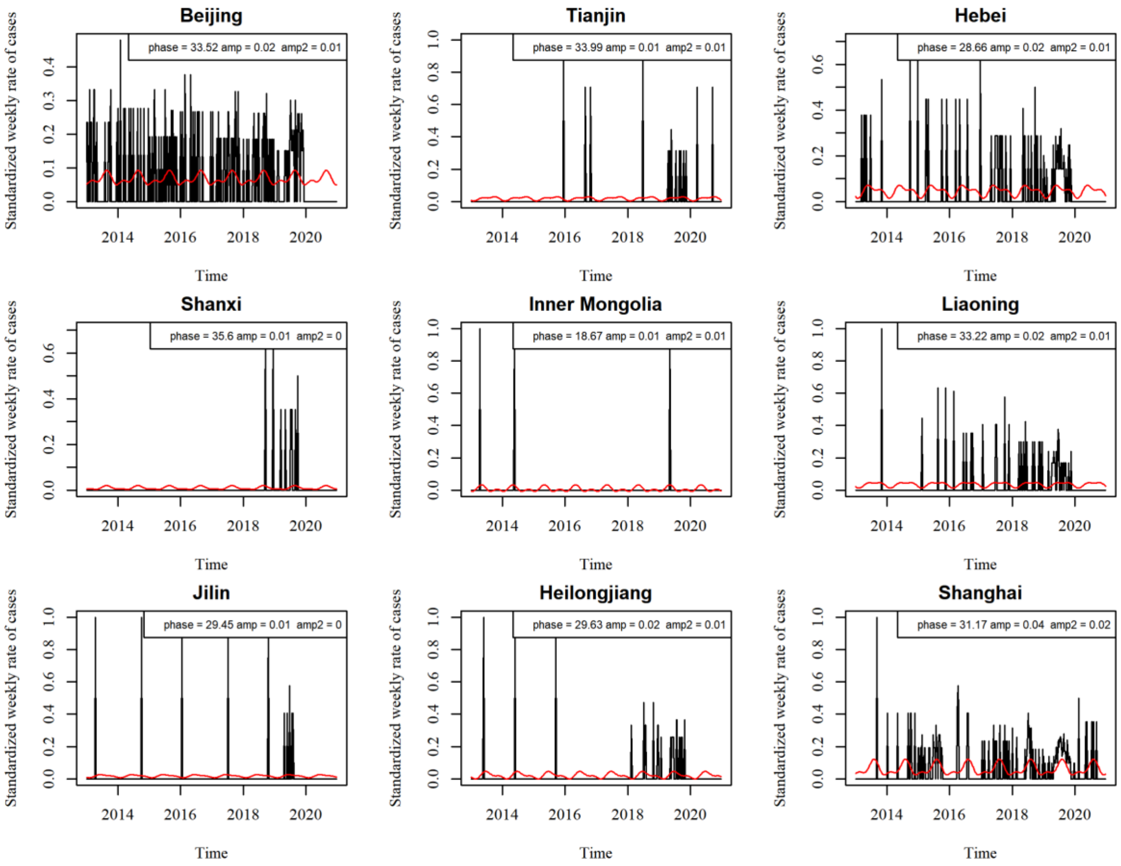

**Supplementary Figure 18. The fit of province-specific seasonal models for imported dengue case in Gansu, Qinghai, Ningxia and Xinjiang.** Black curve represents observed weekly cases standardized by the annual cases; red curve represents the fitted seasonal model based on linear regression with harmonic terms for annual and semi-annual periodicities; amp1 is the annual amplitude and amp2 is the semiannual amplitude.

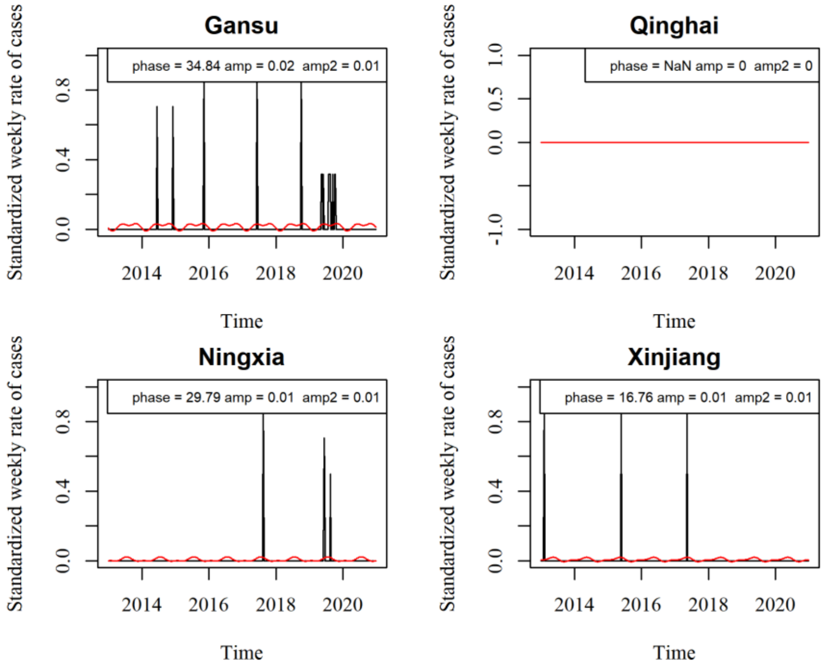

**Supplementary Figure 19. Latitudinal gradients in seasonal epidemic of imported dengue cases in the 31 provincial administrations in mainland China.** Plots represent estimates of seasonal parameters as a function of latitude for dengue fever. (a) Amplitude of annual periodicity. (b) Amplitude of semiannual periodicity. (c) Peak timing in weeks. The centre of the open circle represents point estimates from seasonal regression models. Symbol size is proportional to the province-specific number of local dengue cases , while colours represent different climatic zones (blue, mid-temperate; green, warm-temperate; black, cold-temperate; orange, subtropic; red, tropic). The black lines represent linear regression of province-specific seasonal parameters against latitude, with the *P*-value of regression model presented on the graphs.

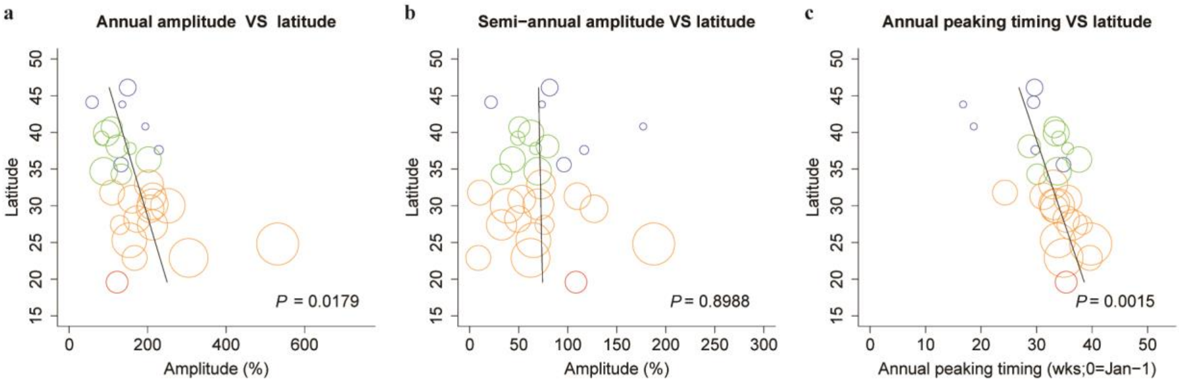

**Supplementary Figure 20. The flowchart of model construction**

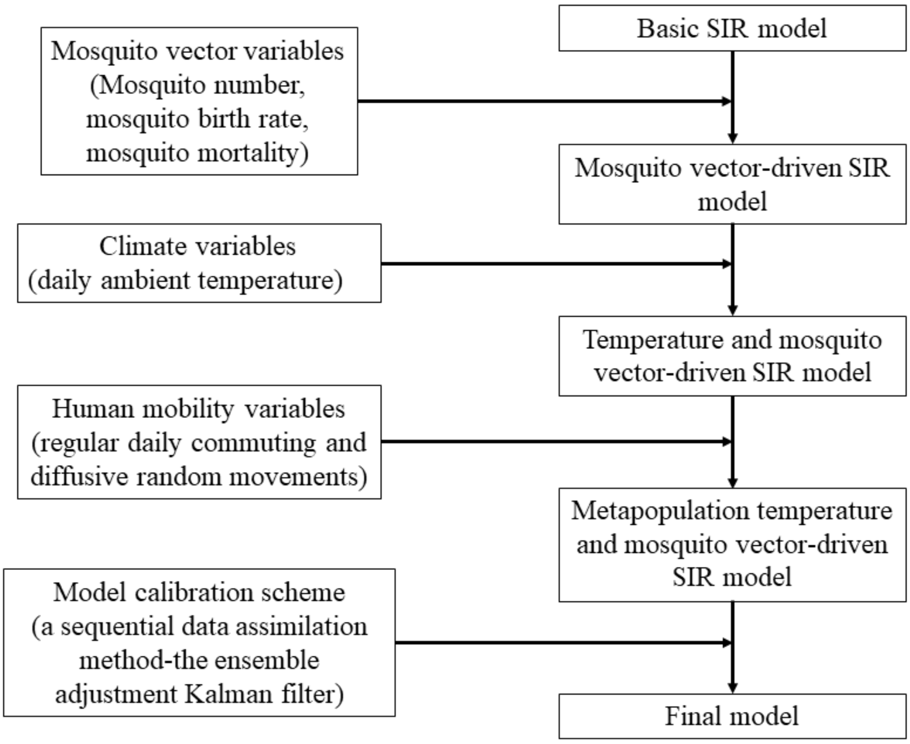

**Supplementary Figure 21. The variations of transmission rate in 337 cities in mainland China.** The results revealed the variations of transmission rate in 337 cities in mainland China at different time points. Different colours depths were adopted to represent the data of the transmission rate. The darker the blue colour, the higher the transmission rate in the location.

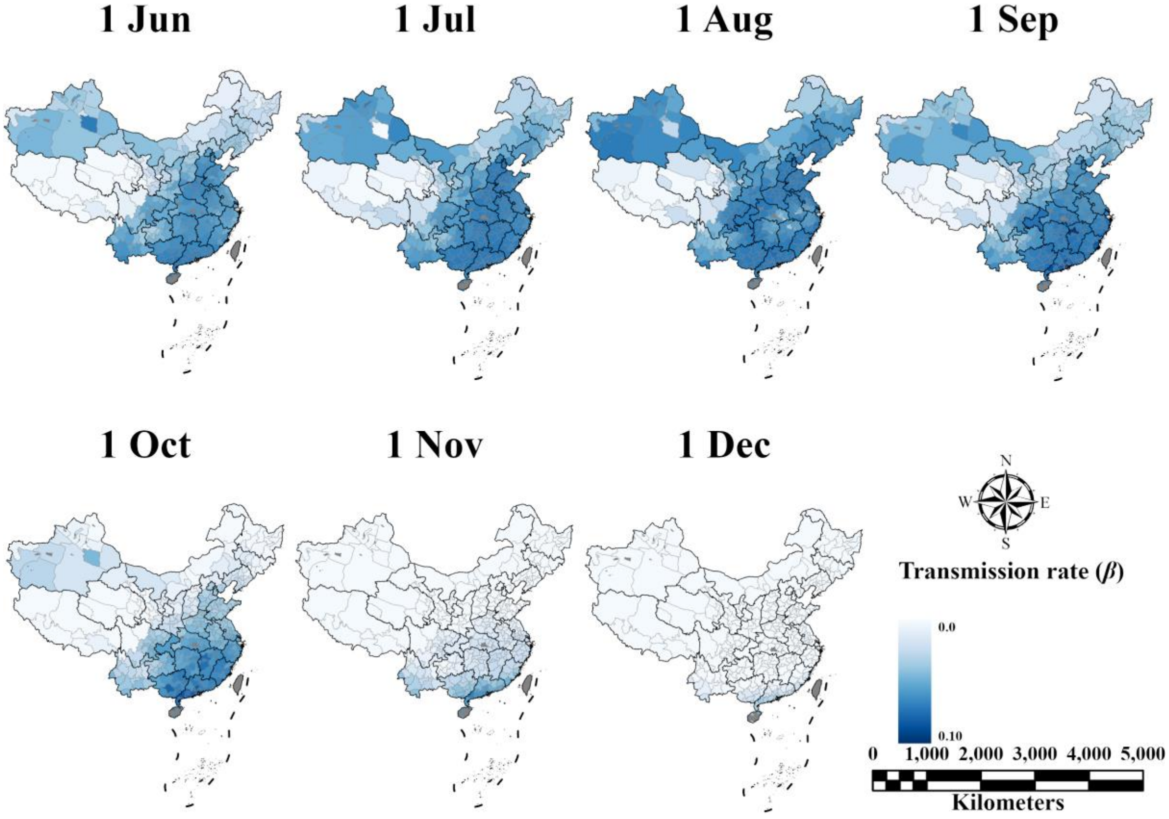

**Supplementary Figure 22. Relationship between commuting intensity and estimated infections due to population movements among cities in China, 2019.** The dots from yellow to red represent the number of infections due to population movements at different scales in each city. The thin to thick blue lines indicate low to high average inter-annual commuting intensity between the two cities.

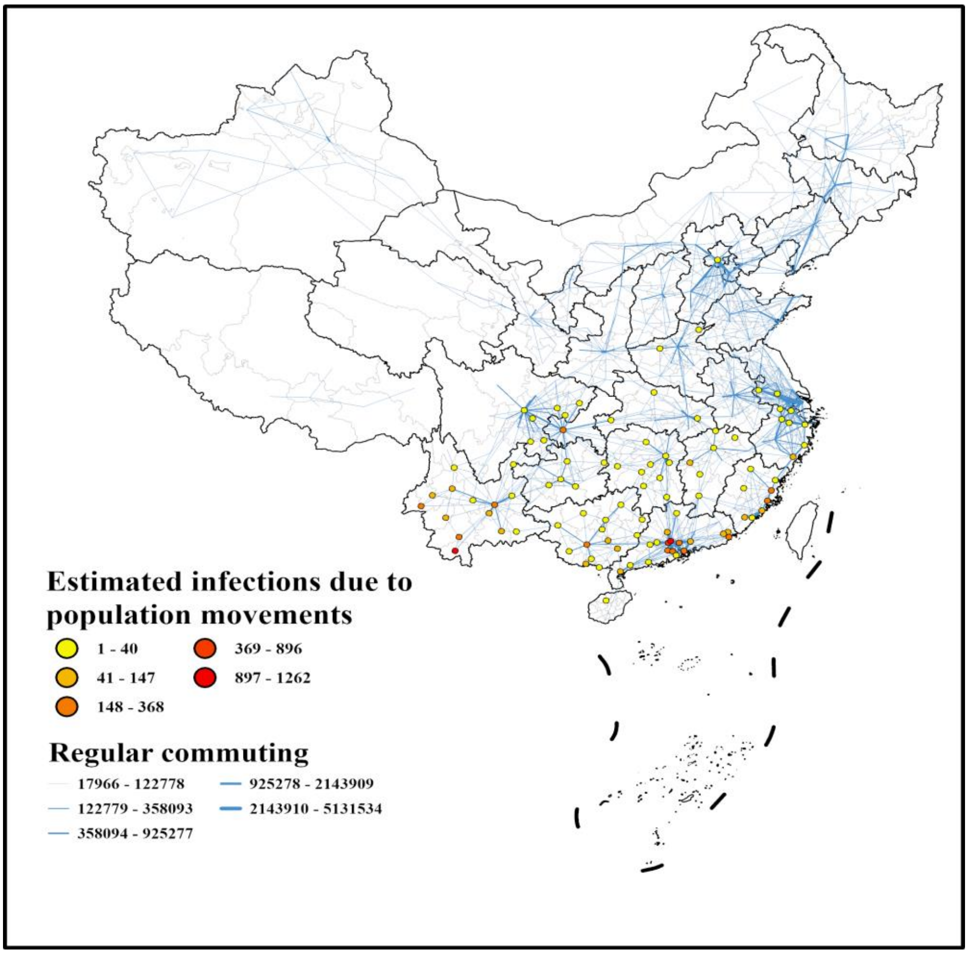

**Supplementary Figure 23. Prior results in four urban agglomerations and in national scale.** The result is the direct output (without data assimilation) of the transmission model in each time step. Distributions are obtained from $n = 100$ ensemble members. In the first row, the blue solid line represents the median, and the black dotted line represents 95% CIs. In the second row, the blue bars represent the medians and whiskers show 95% CIs.

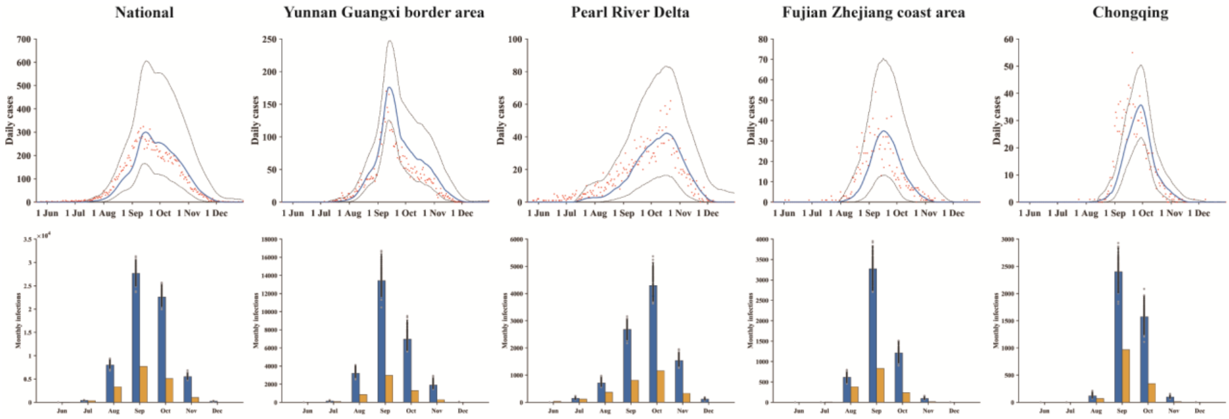

**Supplementary Figure 24. Parameter inference for simulated outbreaks.** The results show the fitting of the national case number using prescribed scenarios and the parameter estimation of the simulated outbreak in Xishuangbanna, Guangzhou, Fujian, Zhejiang and Chongqing. The first column of pictures shows that the initial parameter setting contains “real” parameters; the second column shows that the initial parameter setting is lower than the “real” parameter; the third column shows that the initial parameter setting is higher than the “real” parameter. The black solid line and dotted line represent the median estimate and 95% CIs respectively. Distributions are obtained from $n = 100$ ensemble members.

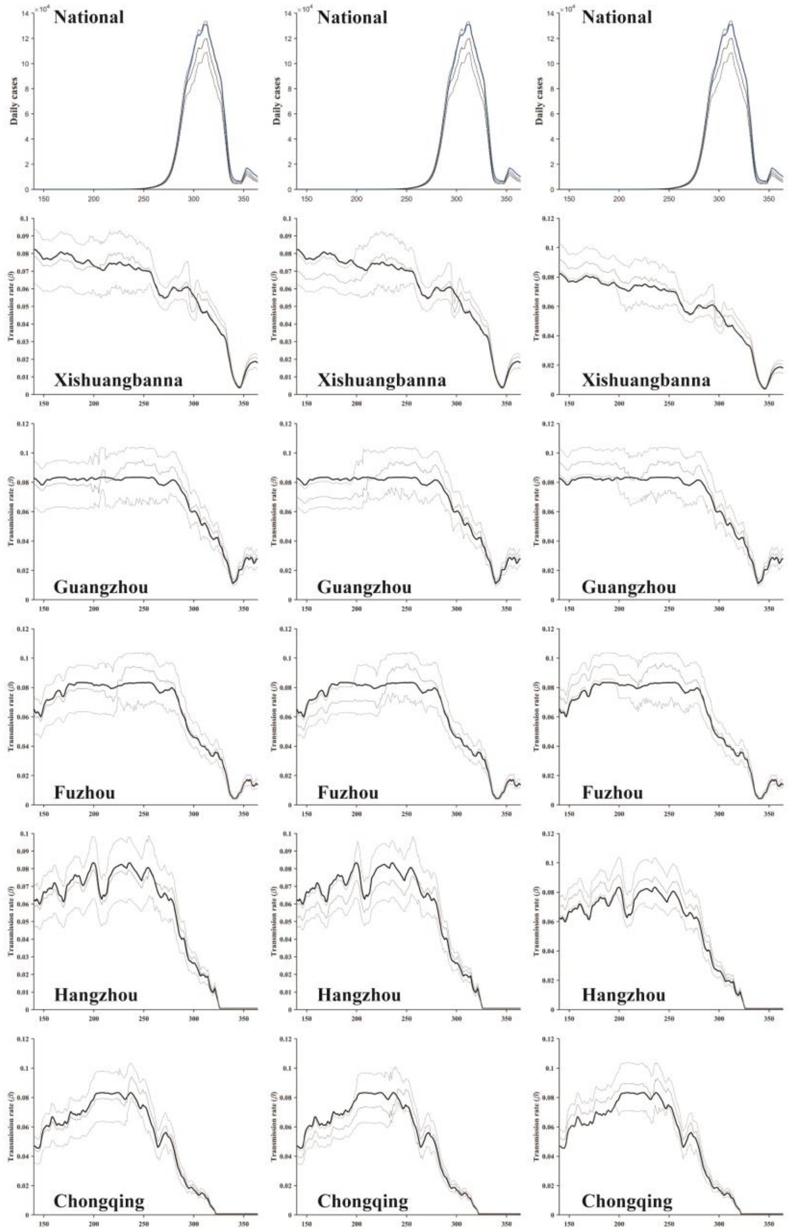

**Supplementary Figure 25. Sensitivity analyses on inference results.** The first line represents the transmission model inference results of the four urban agglomerations increasing infection time $D$. The inference results come from modified transmission model. Fitting the case data (the first line), estimated transmission rate (the second line), effective reproduction number (the third line) and force of infection (the fourth line) are given. Distributions are obtained from $n = 100$ ensemble members. In the first line, the blue solid line represents the median, and the dotted line shows 95% CIs. In the following lines, the centre and box boundaries represent the median, 25th and 75th percentiles, and the beard represents the minimum and maximum.

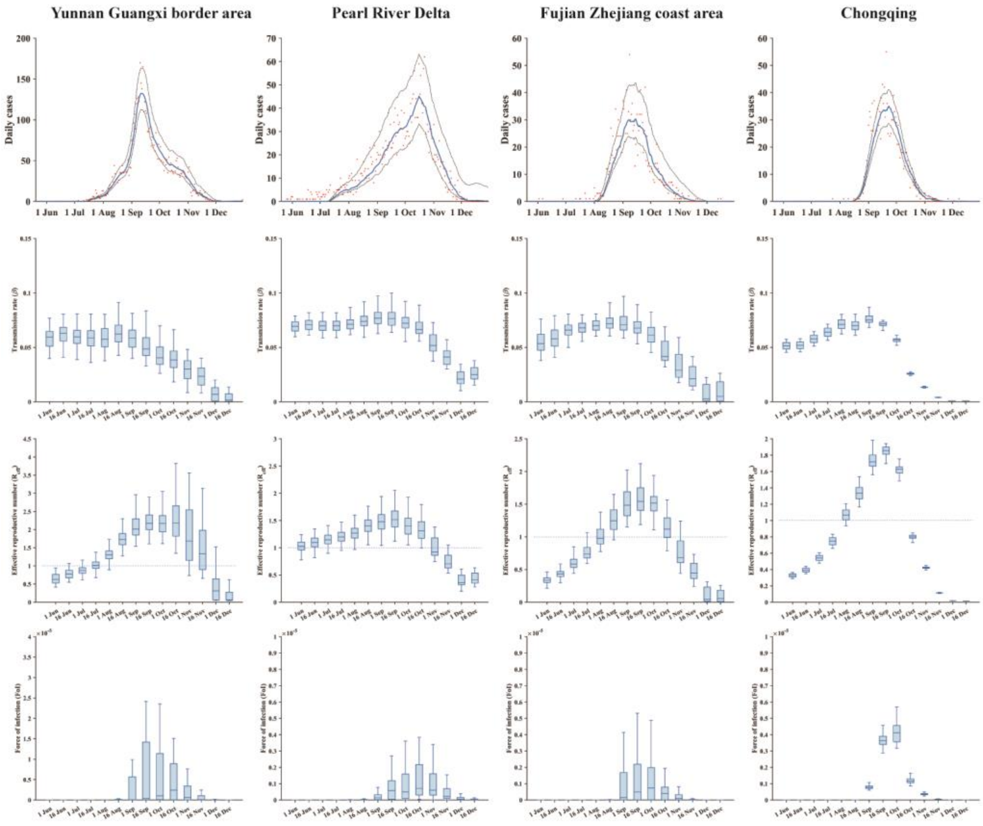

**Supplementary Figure 26. Sensitivity analyses on inference results.** The first line represents the transmission model inference results of the four urban agglomerations reducing the reporting rate $\alpha$. The inference results come from modified transmission model. Fitting the case data (the first line), estimated transmission rate (the second line), effective reproduction number (the third line) and force of infection (the fourth line) are given. Distributions are obtained from $n = 100$ ensemble members. In the first line, the blue solid line represents the median, and the dotted line shows 95% CIs. In the following lines, the centre and box boundaries represent the median, 25th and 75th percentiles, and the beard represents the minimum and maximum.

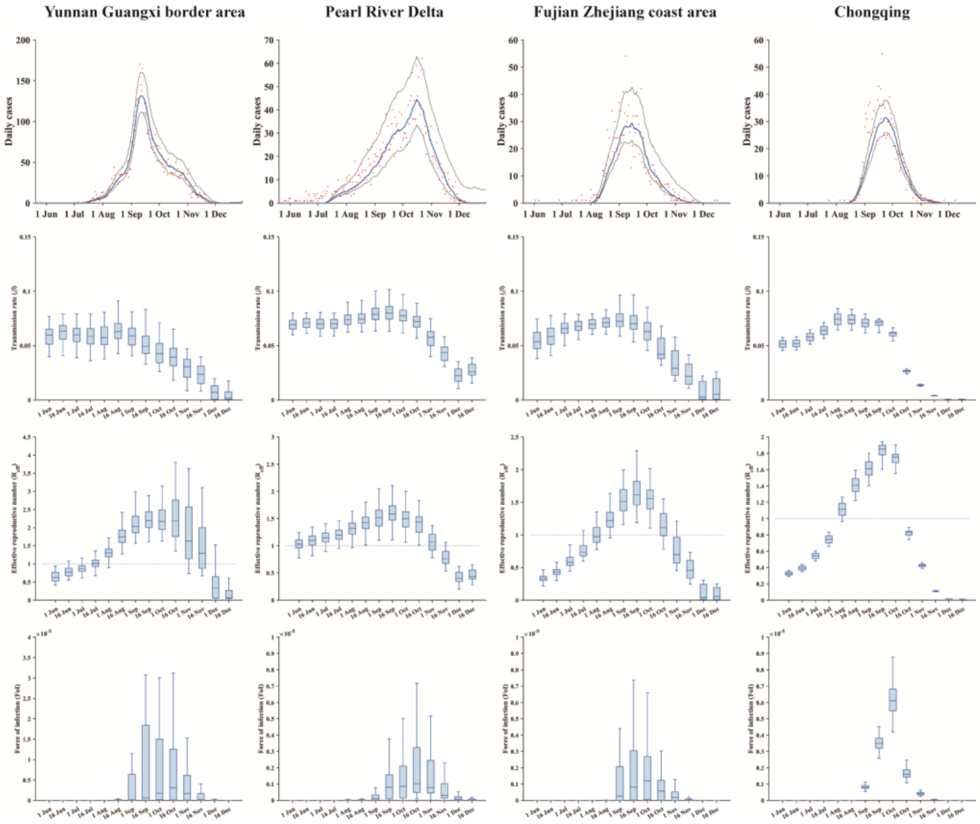

**Supplementary Figure 27. Sensitivity analyses on inference results.** The first line represents the transmission model inference results of the four urban agglomerations increasing the reporting rate $\alpha$. The inference results come from modified transmission model. Fitting the case data (the first line), estimated transmission rate (the second line), effective reproduction number (the third line) and force of infection (the fourth line) are given. Distributions are obtained from $n = 100$ ensemble members. In the first line, the blue solid line represents the median, and the dotted line shows 95% CIs. In the following lines, the centre and box boundaries represent the median, 25th and 75th percentiles, and the beard represents the minimum and maximum.

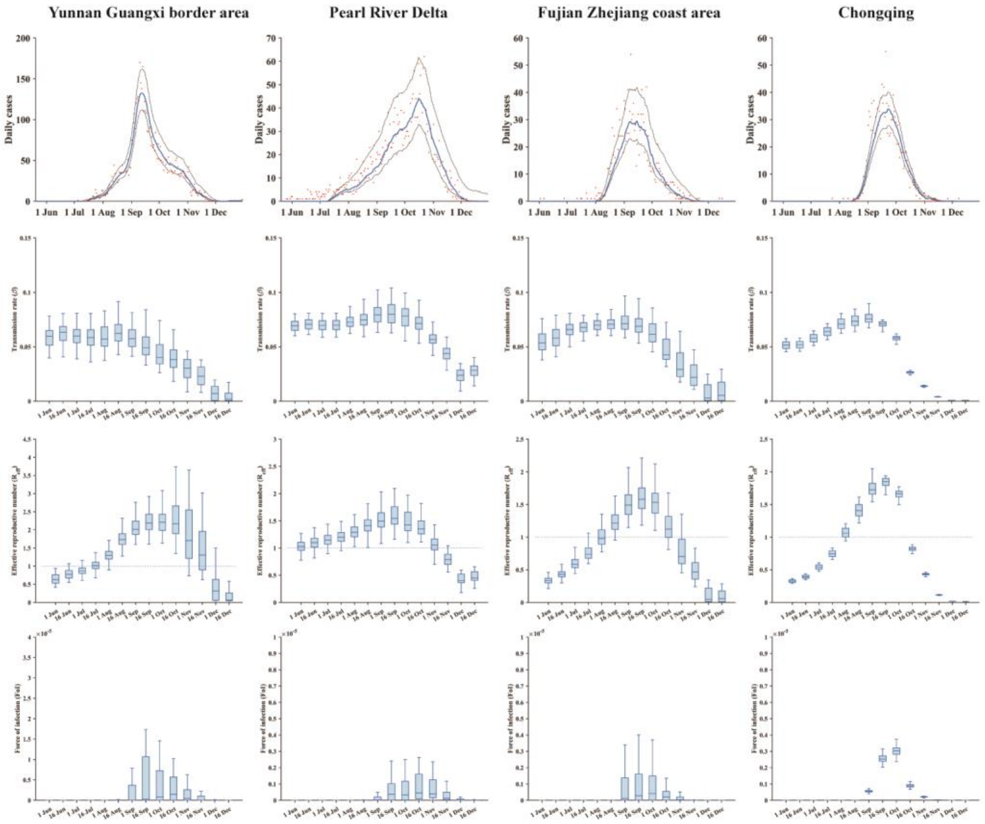

**Supplementary Figure 28. Sensitivity analyses on inference results.** The first line represents the transmission model inference results of the four urban agglomerations increasing the random moving rate $\theta$. The inference results come from modified transmission model. Fitting the case data (the first line), estimated transmission rate (the second line), effective reproduction number (the third line) and force of infection (the fourth line) are given. Distributions are obtained from $n = 100$ ensemble members. In the first line, the blue solid line represents the median, and the dotted line shows 95% CIs. In the following lines, the centre and box boundaries represent the median, 25th and 75th percentiles, and the beard represents the minimum and maximum.

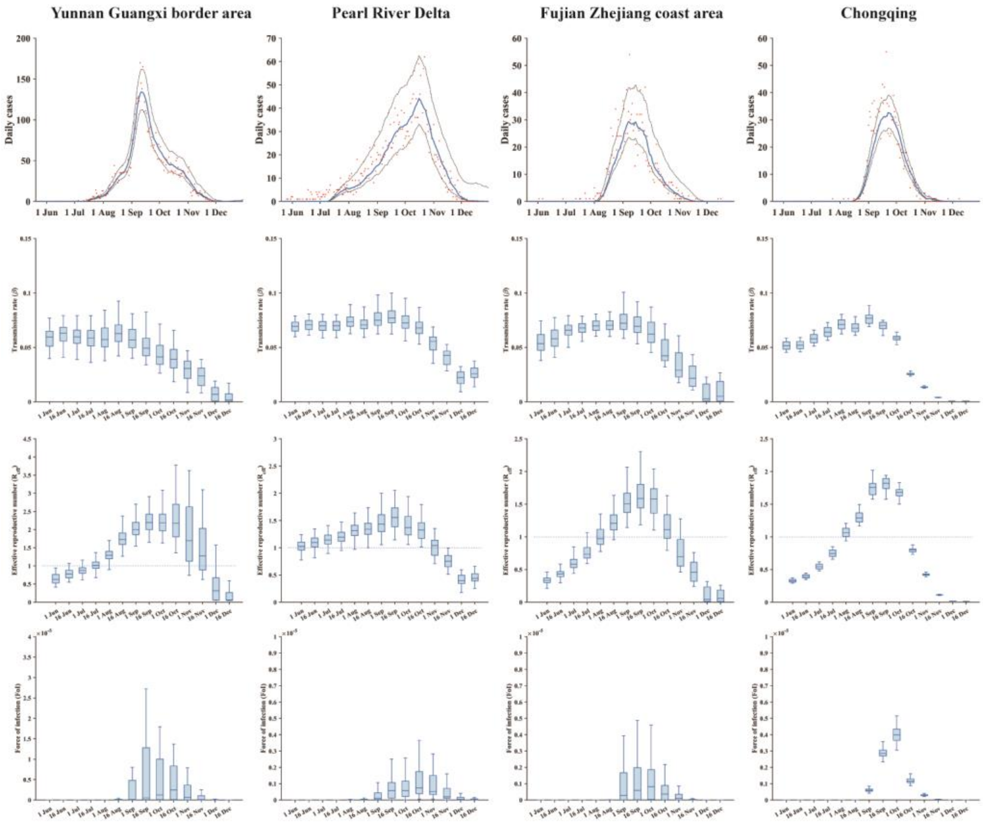

**Supplementary Figure 29. Sensitivity analyses on inference results.** The first line represents the transmission model inference results of the four urban agglomerations changing the initial transmission rates. The inference results come from modified transmission model. Fitting the case data (the first line), estimated transmission rate (the second line), effective reproduction number (the third line) and force of infection (the fourth line) are given. Distributions are obtained from $n = 100$ ensemble members. In the first line, the blue solid line represents the median, and the dotted line shows 95% CIs. In the following lines, the centre and box boundaries represent the median, 25$^{th}$ and 75$^{th}$ percentiles, and the beard represents the minimum and maximum.

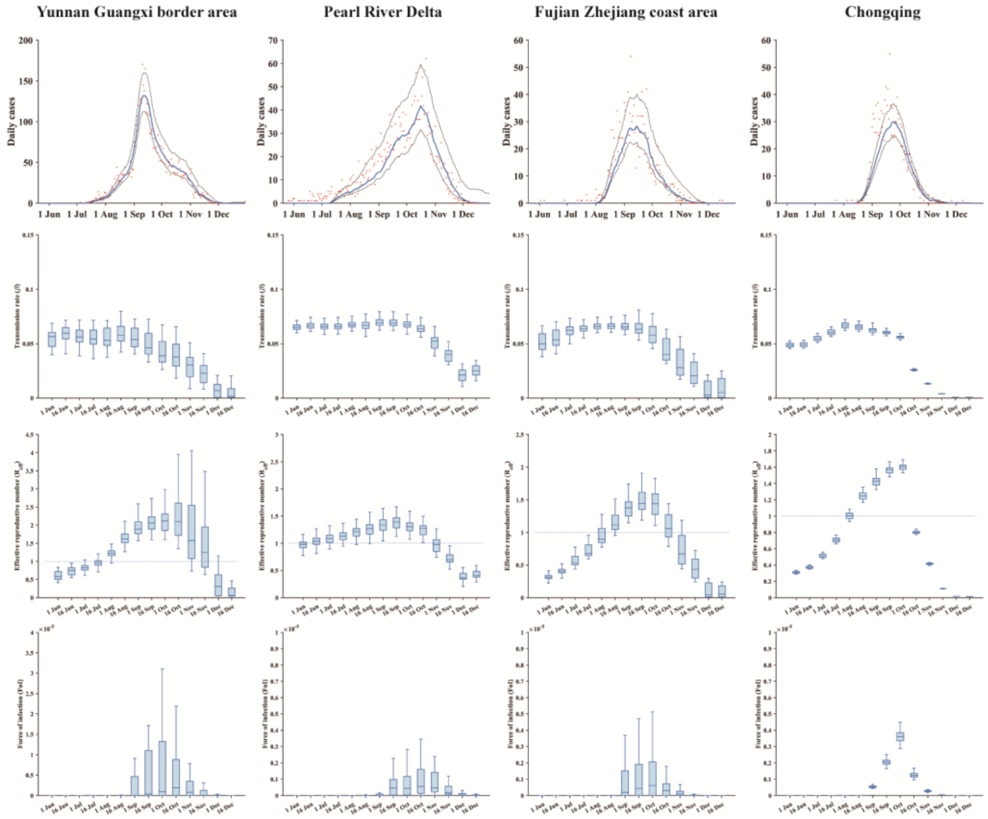

**Supplementary Figure 30. Fitting and inference results of dengue fever infection in China from 2013 to 2018.** (a) The daily fitting results of dengue fever infection in China from 2013 to 2018. The blue solid line represents the median, and the dotted line shows 95% CIs. (b) Estimate the monthly total infection (blue bars) and confirmed cases (orange bars) in China from 2013 to 2018. The blue bars represent the medians and whiskers show 95% CIs. Distributions are obtained from $n = 100$ ensemble members.

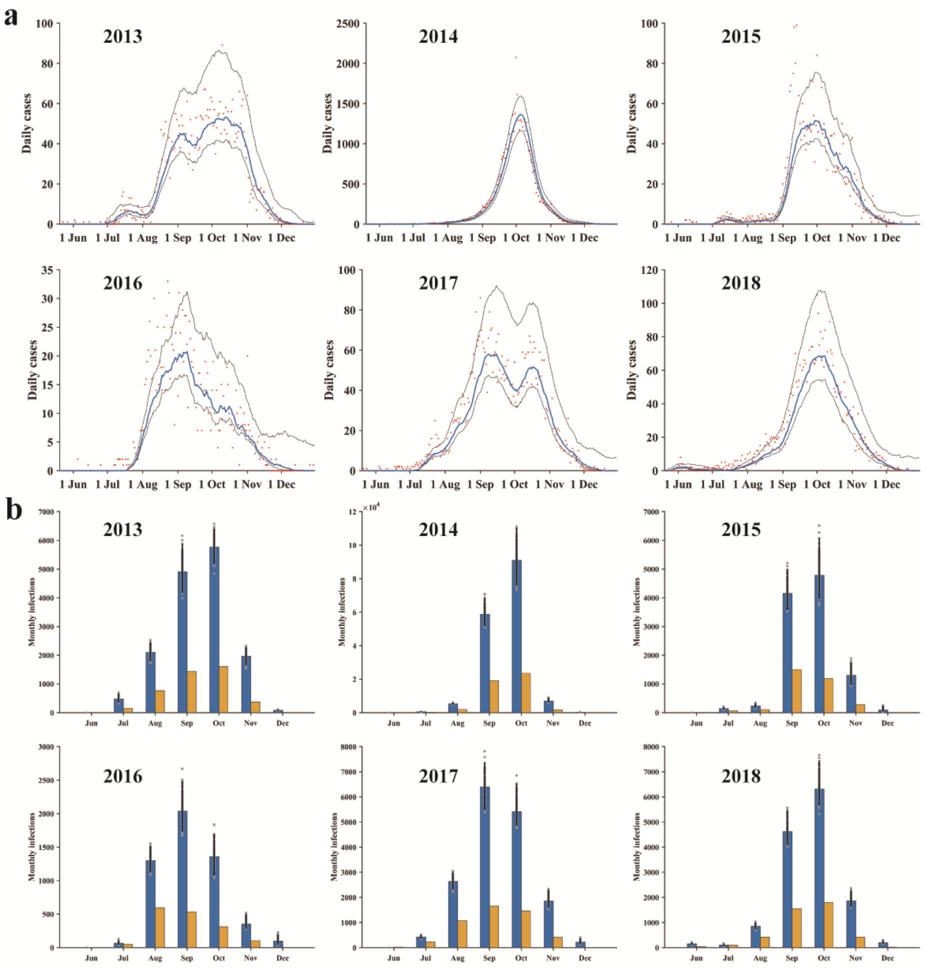

## Tables

**Supplementary Table 1. Background characteristics of 31 provincial administrations in mainland China.**

| Province | Climate | Geographic regions | Latitude (°N) | Longitude (°E) | Population Size | Inflow population | Outflow population |
|---|---|---|---|---|---|---|---|
| Anhui | Sub-tropic region | East | 31.8 | 117.5 | 61027171 | 7797284 | 1550509 |
| Beijing | Warm-temperate region | North | 39.9 | 116.4 | 21893095 | 2345582 | 8418418 |
| Fujian | Sub-tropic region | East | 25.3 | 118.8 | 41540086 | 6104165 | 4889876 |
| Gansu | Mid-temperate region | Northwest | 35.6 | 104.7 | 25019831 | 2722616 | 765648 |
| Guangdong | Sub-tropic region | South | 22.9 | 113.4 | 126012510 | 21170281 | 29622110 |
| Guangxi | Sub-tropic region | South | 22.9 | 108.4 | 50126804 | 6132927 | 1359384 |
| Guizhou | Sub-tropic region | Southwest | 27.4 | 106.8 | 38562148 | 4876051 | 1146546 |
| Hainan | Tropic region | South | 19.6 | 110.1 | 4861346 | 1116895 | 837278 |
| Hebei | Warm-temperate region | North | 38.1 | 115.8 | 74610235 | 6730356 | 3155272 |
| Henan | Warm-temperate region | Central | 34.7 | 113.1 | 98638254 | 10372336 | 1260002 |
| Heilongjiang | Mid-temperate region | Northeast | 46.1 | 126.2 | 31850088 | 5735819 | 829176 |
| Hubei | Sub-tropic region | Central | 30.9 | 113.6 | 54372084 | 7778308 | 2217954 |
| Hunan | Sub-tropic region | Central | 27.4 | 113 | 66444864 | 7287335 | 1577563 |
| Jilin | Mid-temperate region | Northeast | 44.1 | 125.4 | 24073453 | 4877239 | 1001471 |
| Jiangsu | Sub-tropic region | East | 32.9 | 118.6 | 84748016 | 10174114 | 10308610 |
| Jiangxi | Sub-tropic region | East | 28.2 | 115.3 | 45188635 | 4095233 | 1279014 |
| Liaoning | Warm-temperate region | Northeast | 40.7 | 122.6 | 42591407 | 6643290 | 2847308 |
| Inner mongolia | Mid-temperate region | North | 40.8 | 110.8 | 24049155 | 4998738 | 1686420 |
| Ningxia | Mid-temperate region | Northwest | 37.6 | 106 | 7202654 | 1202742 | 675119 |
| Qinghai | Cold region | Northwest | 36.6 | 101.8 | 5923957 | 822258 | 417304 |
| Shandong | Warm-temperate region | East | 36.3 | 118.4 | 101527453 | 9973071 | 4129007 |
| Shanxi | Warm-temperate region | North | 37.8 | 112.8 | 34915616 | 4662300 | 1620518 |

| Province | Climate | Geographic regions | Latitude (°N) | Longitude (°E) | Population Size | Inflow population | Outflow population |
|---|---|---|---|---|---|---|---|
| Shaanxi | Warm-temperate region | Northwest | 34.3 | 108.8 | 39528999 | 5363097 | 1933712 |
| Shanghai | Sub-tropic region | East | 31.3 | 121.5 | 24870895 | 2247193 | 10479652 |
| Sichuan | Sub-tropic region | Southwest | 30.2 | 104 | 83674866 | 13539713 | 2590041 |
| Tianjin | Warm-temperate region | North | 39.2 | 117.2 | 13866009 | 1550648 | 3534816 |
| Tibet | Cold region | Southwest | 31.4 | 89.2 | 3648100 | 370957 | 407121 |
| Xinjiang | Mid-temperate region | Northwest | 43.8 | 87.6 | 24278414 | 2276694 | 3116520 |
| Yunnan | Sub-tropic region | Southwest | 24.8 | 103 | 47209277 | 5512348 | 2230394 |
| Zhejiang | Sub-tropic region | East | 30 | 120.4 | 64567588 | 5776998 | 16186454 |
| Chongqing | Sub-tropic region | Southwest | 29.6 | 106.6 | 32054159 | 4593532 | 2193575 |

**Supplementary Table 2. The main method relevant findings of the studies about dengue fever in China.**

| **Publication** | **Study Region** | **NO. of dengue cases** | **Period of reporting dengue cases** | **Main method** | **Main relevant findings** |
|---|---|---|---|---|---|
| Li et al., 2023[15] | 365 cities in China | 93,101 dengue cases | 1 January 2013 to 31 December 2019 | The spatiotemporal Bayesian hierarchical model | Substantial burden of dengue morbidity is attributable to non-optimal hydrometeorological conditions under climate-change scenarios. |
| Yue et al., 2021[16] | 1211 counties of 28 provinces in China | 22,246 dengue cases | 1 January 2019 to 18 December 2019 | The discrete Poisson model | Dengue fever was very prevalent and widely distributed in mainland China in 2019. |
| Lu et al., 2022[17] | 124 counties of Guangdong province in China | 44,939 (44,862 local and 77 imported) dengue cases | In 2014 | The negative binomial regression model | The dengue virus may be more transmissible in a region with warmer weather conditions. |

| | | | | | |
|---|---|---|---|---|---|
| Zhao et al., 2023[18] | In China | 94,353 (81,652 local and 12,701 imported) dengue cases | 1 January 2006 to 31 December 2020 | The vector auto-regression model, The generalized additive models, the structural equation modeling | Dengue outbreaks in China are triggered by introductions of imported cases and boosted by landscape features and connectivity. |
| Li et al., 2022[19] | Guangzhou city in China | 7,916 dengue cases | From June to October 2015–2019 | The quasi-Poisson generalized linear model, the distributed lag non-linear model | The tropical cyclones may increase the incidence of dengue within a 4-week lag in Guangzhou, China, and the effects were more pronounced in men and the elderly. |
| Liu et al., 2021[20] | A community and a nearby construction site in Chikan District of | 467 dengue cases | In 2018 | The Susceptible-Exposed-Infectious/Asymptomatic-Recovered model | To control the outbreak of dengue fever effectively on both the construction site and in the community, interventions needed to be made both within the community and from the community to the construction site. |

| | | | | | |
|---|---|---|---|---|---|
| | Zhanjiang city in China | | | | |
| Cheng et al., 2021[21] | Guangdong province in China | 45,111 local dengue cases | May to November in 2014 | The bayesian spatial conditional autoregressive model | Spatially varied effects of weather conditions on dengue outbreaks necessitate area-specific dengue prevention and control measures. Extremes of temperature and rainfall have strong and positive associations with dengue outbreaks. |
| Chen et al., 2022[22] | Guangzhou city in China | 46,206 (45,863 local and 343 imported) dengue cases. | 1 January 2005 to 31 December 2016 | The generalized additive models | A forecast model for dengue epidemic was established with good forecast effects and may have a potential application in global dengue endemic areas after modification according to local meteorological conditions. |

| | | | | | |
|---|---|---|---|---|---|
| Li et al., 2021[23] | 9 cities in the Pearl River Delta in China | 47,784 dengue cases | June and October, 2013–2018 | The time stratified case-crossover design combined with conditional Poisson regression model | Tropical cyclones are associated with increased risk of local dengue fever incidence in south China, with the elderly more vulnerable than other population subgroups. |
| Guo et al., 2023[24] | Xiamen city in China | 138 (19 local and 119 imported) dengue cases | In 2019 | The transmission dynamics model | The mosquito resistance index has an important influence on the local transmission of dengue fever caused by imported cases in Xiamen, and the Brayton index can also a |
| Li et al., 2023[25] | 54 cities in 4 coastal provinces in southeast China | 70,006 dengue cases | 1 January 2013 to 31 December 2019 | The Standardized Precipitation Evapotranspiration Index | The extreme hydrological conditions were associated with increased dengue fever incidence within a 6-month lag period, with different dimensions of city development playing various modification roles in this association. |

| | | | | |
|---|---|---|---|---|
| Chen et al., 2022[26] | Guangzhou city in China | From 2011 to 2017 | The ensemble forecast system based on a susceptible-infected-recovered (SIR) type of compartmental model | The ensemble forecast system can be operated in real-time and inform control planning to reduce the burden of dengue fever. |
| Zeng et al., 2023[27] | 21 cities in Guangdong of southeastern China | From 2015 to 2020 | The network model for spatiotemporal transmission prediction of dengue fever using metapopulation networks based on human mobility | The metapopulation network-EAKF system provided accurate predictions for city-level dengue transmission trajectories in retrospective forecasts of 12 cities in Guangdong province, China. |

**Supplementary Table 3. The information of 337 cities in China during 2013-2020.**

| Province | City | Dengue incidence (per 10,000,000) | | NO. of total flowing people |
|---|---|---|---|---|
| | | Local | Import | |
| Beijing | Beijing | 10.9624 | 79.0203 | 10764000 |
| Tianjin | Tianjin | 2.8848 | 11.5390 | 5085464 |
| Hebei | Shijiazhuang | 2.6702 | 16.0212 | 2556980 |
| | Tangshan | 0.0000 | 14.2524 | 1032858 |
| | Qinghuangdao | 6.3758 | 25.5031 | 601067 |
| | Handan | 1.0622 | 7.4357 | 625816 |
| | Xingtai | 4.2188 | 2.8125 | 502340 |
| | Baoding | 7.7962 | 23.3887 | 1201295 |
| | Zhangjiakou | 0.0000 | 0.0000 | 679280 |
| | Chengde | 0.0000 | 5.9622 | 360112 |
| | Cangzhou | 2.7394 | 9.5880 | 693507 |
| | Langfang | 0.0000 | 10.9808 | 1235343 |
| | Hengshui | 0.0000 | 9.4946 | 397030 |
| Shanxi | Taiyuan | 3.7707 | 9.4267 | 2342035 |
| | Datong | 0.0000 | 0.0000 | 586493 |
| | Yangquan | 0.0000 | 0.0000 | 259828 |
| | Changzhi | 0.0000 | 0.0000 | 404960 |
| | Jincheng | 0.0000 | 0.0000 | 338965 |
| | Shuozhou | 12.5514 | 6.2757 | 368588 |
| | Jinzhong | 0.0000 | 0.0000 | 498658 |
| | Yuncheng | 4.1889 | 4.1889 | 417862 |
| | Xinzhou | 0.0000 | 0.0000 | 270934 |
| | Linfen | 0.0000 | 2.5148 | 373569 |

| | | | | |
|---|---|---|---|---|
| | Lvliang | 0.0000 | 2.9425 | 420926 |
| Inner Mongolia | Hohhot | 0.0000 | 0.0000 | 1690404 |
| | Baotou | 0.0000 | 0.0000 | 1063364 |
| | Wuhai | 0.0000 | 0.0000 | 219183 |
| | Chifeng | 2.4777 | 2.4777 | 619388 |
| | Tongliao | 0.0000 | 0.0000 | 411471 |
| | Ordos | 0.0000 | 0.0000 | 915645 |
| | HulunBuir | 0.0000 | 0.0000 | 448644 |
| | BayanNur | 0.0000 | 0.0000 | 311849 |
| | Ulanqab | 0.0000 | 0.0000 | 316517 |
| | HingganLeague | 0.0000 | 7.0575 | 215730 |
| | XilingolLeague | 0.0000 | 0.0000 | 367559 |
| | AlxaLeague | 38.1154 | 38.1154 | 105404 |
| Liaoning | Shenyang | 6.6151 | 33.0757 | 3159825 |
| | Dalian | 4.0264 | 14.7635 | 2594972 |
| | Anshan | 0.0000 | 3.0072 | 439901 |
| | Fushun | 0.0000 | 26.8619 | 242110 |
| | Benxi | 7.5414 | 15.0828 | 211859 |
| | Dandong | 4.5695 | 18.2779 | 267100 |
| | Jinzhou | 7.3969 | 11.0953 | 406807 |
| | Yingkou | 4.2945 | 0.0000 | 470755 |
| | Fuxin | 0.0000 | 6.0706 | 200079 |
| | Liaoyang | 0.0000 | 6.2322 | 313154 |
| | Panjin | 0.0000 | 14.3917 | 362698 |
| | Tieling | 0.0000 | 8.3742 | 266427 |
| | Chaoyang | 0.0000 | 6.9617 | 263944 |
| | Huludao | 0.0000 | 8.2163 | 290967 |

| | | | | |
|---|---|---|---|---|
| Jilin | Changchun | 3.3087 | 2.2058 | 3162392 |
| | Jilin | 2.7596 | 2.7596 | 750033 |
| | Siping | 0.0000 | 5.5105 | 295961 |
| | Liaoyuan | 20.0621 | 10.0311 | 180956 |
| | Tonghua | 0.0000 | 5.5184 | 242080 |
| | Baishan | 0.0000 | 0.0000 | 156593 |
| | Songyuan | 0.0000 | 4.4385 | 458506 |
| | Baicheng | 0.0000 | 6.4459 | 233019 |
| | Yanbian Chaoxianzu (Korean) Autonomous Prefecture | 15.1032 | 15.1032 | 399170 |
| Heilongjiang | Harbin | 1.9980 | 14.9852 | 2660771 |
| | Qiqihar | 0.0000 | 0.0000 | 485999 |
| | Jixi | 0.0000 | 6.6575 | 272493 |
| | Hegang | 0.0000 | 0.0000 | 203974 |
| | Shuangyashan | 0.0000 | 16.5453 | 221738 |
| | Daqing | 7.1902 | 7.1902 | 737906 |
| | Yichun | 11.3781 | 0.0000 | 115712 |
| | Jiamusi | 4.6371 | 4.6371 | 517671 |
| | Qitaihe | 0.0000 | 29.0019 | 189024 |
| | Mudanjiang | 0.0000 | 13.0992 | 456632 |
| | Heihe | 0.0000 | 0.0000 | 186683 |
| | Suihua | 7.9869 | 2.6623 | 440506 |
| | Da Hinggan Ling Prefecture | 0.0000 | 0.0000 | 75886 |
| Shanghai | Shanghai | 10.8561 | 80.0132 | 12726845 |
| Jiangsu | Nanjing | 12.8829 | 31.1336 | 3423521 |
| | Wuxi | 1.3401 | 38.8629 | 2861489 |
| | Xuzhou | 3.3026 | 5.5043 | 905628 |
| | Changzhou | 7.5785 | 85.2576 | 1917470 |

| | | | | |
|---|---|---|---|---|
| | Suzhou | 2.3533 | 36.0834 | 5878776 |
| | Nantong | 0.0000 | 67.2997 | 1471653 |
| | Lianyungang | 2.1742 | 36.9617 | 380971 |
| | Huaian | 2.1948 | 28.5324 | 419408 |
| | Yancheng | 5.9616 | 22.3559 | 551479 |
| | Yangzhou | 4.3862 | 54.8270 | 814841 |
| | Zhenjiang | 0.0000 | 37.3783 | 876991 |
| | Taizhou | 13.2956 | 57.6144 | 611809 |
| | Suqian | 0.0000 | 8.0222 | 368688 |
| Zhejiang | Hangzhou | 1024.6305 | 208.6124 | 5077347 |
| | Ningbo | 93.5744 | 127.6014 | 3987478 |
| | Wenzhou | 226.6815 | 123.2646 | 3115575 |
| | Jiaxing | 40.7342 | 83.3199 | 2055171 |
| | Huzhou | 11.8780 | 53.4509 | 950885 |
| | Shaoxing | 32.2521 | 159.3632 | 1376796 |
| | Jinhua | 29.7843 | 191.4708 | 2760258 |
| | Quzhou | 65.8998 | 109.8329 | 284289 |
| | Zhoushan | 77.7325 | 103.6433 | 324468 |
| | Taizhou | 61.9065 | 209.8782 | 1661959 |
| | Lishui | 7.9764 | 91.7286 | 369226 |
| Anhui | Hefei | 2.1345 | 44.8245 | 3665689 |
| | Wuhu | 2.7439 | 35.6710 | 786920 |
| | Bengbu | 3.0336 | 18.2016 | 530222 |
| | Huainan | 3.2965 | 32.9649 | 418407 |
| | Maanshan | 4.6298 | 37.0382 | 404084 |
| | Huaibei | 5.0755 | 5.0755 | 308437 |
| | Tongling | 0.0000 | 15.2471 | 209350 |

| | | | | |
|---|---|---|---|---|
| | Anqing | 7.2024 | 43.2143 | 433980 |
| | Huangshan | 7.5156 | 15.0312 | 199103 |
| | Chuzhou | 2.5081 | 25.0812 | 565592 |
| | Fuyang | 4.8779 | 13.4142 | 480455 |
| | Suzhou | 0.0000 | 11.2687 | 270812 |
| | Lu'an | 2.2760 | 6.8280 | 485323 |
| | Bozhou | 2.0013 | 6.0038 | 187708 |
| | Chizhou | 0.0000 | 7.4473 | 131894 |
| | Xuancheng | 0.0000 | 23.9994 | 269817 |
| Fujian | Fuzhou | 1839.2844 | 253.2785 | 2603631 |
| | Amoy | 69.7138 | 302.0932 | 3026978 |
| | Putian | 1756.6186 | 252.2803 | 564014 |
| | Sanming | 185.0027 | 92.5014 | 349868 |
| | Quanzhou | 128.6681 | 439.5211 | 2704732 |
| | Zhangzhou | 207.7428 | 69.2476 | 758099 |
| | Nanping | 608.0626 | 119.3743 | 270049 |
| | Longyan | 11.0147 | 73.4312 | 387805 |
| | Ningde | 187.4927 | 235.1603 | 328865 |
| Jiangxi | Nanchang | 113.5091 | 89.5283 | 2209825 |
| | Jingdezhen | 61.7673 | 49.4139 | 239952 |
| | Pingxiang | 16.6223 | 27.7038 | 213885 |
| | Jiujiang | 4.3476 | 45.6494 | 556250 |
| | Xinyu | 41.5801 | 58.2121 | 128611 |
| | Yingtan | 8.6638 | 17.3277 | 133610 |
| | Ganzhou | 82.4971 | 35.6744 | 740175 |
| | Jian | 355.7703 | 22.3755 | 310705 |
| | Yichun | 1625.4961 | 35.9446 | 301135 |

| | | | | |
|---|---|---|---|---|
| | Fuzhou | 22.1308 | 24.8972 | 183425 |
| | Shangrao | 10.7840 | 41.5955 | 356674 |
| Shandong | Jinan | 3.2600 | 20.6467 | 2112829 |
| | Qingdao | 3.9715 | 32.7650 | 2726625 |
| | Zibo | 0.0000 | 19.1321 | 856320 |
| | Zaozhuang | 0.0000 | 5.1873 | 223384 |
| | Dongying | 0.0000 | 13.6767 | 508678 |
| | Yantai | 0.0000 | 9.8562 | 1268549 |
| | Weifang | 1.0653 | 7.4574 | 1268360 |
| | Jining | 94.5214 | 17.9471 | 693937 |
| | Taian | 1.8274 | 38.3757 | 700992 |
| | Weihai | 3.4405 | 51.6076 | 722613 |
| | Rizhao | 0.0000 | 0.0000 | 324130 |
| | Linyi | 0.9076 | 9.9833 | 1089509 |
| | Dezhou | 1.7822 | 5.3465 | 528830 |
| | Liaocheng | 0.0000 | 3.3601 | 344000 |
| | Binzhou | 0.0000 | 5.0909 | 426200 |
| | Heze | 3.4107 | 11.3689 | 307122 |
| Henan | Zhengzhou | 7.1425 | 26.1893 | 5168927 |
| | Kaifeng | 6.2189 | 10.3648 | 346018 |
| | Luoyang | 26.9248 | 43.9299 | 1000547 |
| | Pingdingshan | 2.0052 | 12.0310 | 376003 |
| | Anyang | 3.6512 | 34.6866 | 467697 |
| | Hebi | 0.0000 | 6.3858 | 123782 |
| | Xinxiang | 1.5995 | 62.3807 | 555126 |
| | Jiaozuo | 5.6801 | 17.0402 | 320067 |
| | Puyang | 167.0163 | 76.8805 | 393234 |

| | | | | |
|---|---|---|---|---|
| | Xuchang | 70.7763 | 29.6804 | 293281 |
| | Luohe | 4.2239 | 12.6716 | 209703 |
| | Sanmenxia | 0.0000 | 24.5716 | 166234 |
| | Nanyang | 3.0886 | 13.3840 | 742797 |
| | Shangqiu | 5.1172 | 7.6757 | 342209 |
| | Xinyang | 11.2280 | 19.2480 | 418252 |
| | Zhoukou | 7.7554 | 8.8633 | 306343 |
| | Zhumadian | 18.5491 | 18.5491 | 402118 |
| Hubei | Wuhan | 8.0336 | 56.2352 | 5312313 |
| | Huangshi | 24.3006 | 16.2004 | 302084 |
| | Shiyan | 0.0000 | 9.3487 | 640164 |
| | Yichang | 10.6315 | 37.2102 | 677678 |
| | Xiangyang | 7.6032 | 39.9167 | 616786 |
| | Ezhou | 0.0000 | 120.4425 | 191469 |
| | Jingmen | 7.7014 | 30.8056 | 431309 |
| | Xiaogan | 7.0252 | 53.8595 | 363665 |
| | Jingzhou | 24.8510 | 63.0833 | 516347 |
| | Huanggang | 66.2959 | 37.3977 | 322131 |
| | Xianning | 3.7618 | 37.6178 | 218911 |
| | Suizhou | 9.7660 | 53.7130 | 153095 |
| | Enshi Tujia and Miao Autonomous Prefecture | 109.9494 | 60.7615 | 250310 |
| Hunan | Changsha | 129.3801 | 89.5708 | 3789147 |
| | Zhuzhou | 10.2492 | 66.6199 | 748212 |
| | Xiangtan | 58.6902 | 110.0441 | 456560 |
| | Hengyang | 257.3269 | 34.6112 | 647302 |
| | Shaoyang | 181.3052 | 65.5136 | 320945 |
| | Yueyang | 27.7122 | 45.5272 | 483031 |

| | | | | |
|---|---|---|---|---|
| | Changde | 26.5197 | 68.1934 | 426056 |
| | Zhangjiajie | 6.5918 | 52.7347 | 108335 |
| | Yiyang | 31.1562 | 114.2393 | 231804 |
| | Chenzhou | 66.4219 | 47.1381 | 402732 |
| | Yongzhou | 340.2760 | 49.1510 | 238871 |
| | Huaihua | 32.6969 | 32.6969 | 462498 |
| | Loudi | 33.9692 | 81.0035 | 346895 |
| | Xiangxi Tujia and Miao Autonomous Prefecture | 16.0765 | 20.0956 | 202510 |
| Guangdong | Guangzhou | 22672.2148 | 356.5959 | 10458040 |
| | Shaoguan | 70.0493 | 42.0296 | 454391 |
| | Shenzhen | 491.0143 | 317.2444 | 13330374 |
| | Zhuhai | 2525.0196 | 303.3303 | 1286297 |
| | Shantou | 2215.5455 | 94.5106 | 699606 |
| | Foshan | 5171.1452 | 197.9184 | 5138358 |
| | Jiangmen | 2888.6494 | 83.3665 | 1311288 |
| | Zhanjiang | 2091.3202 | 103.1336 | 839130 |
| | Maoming | 296.4019 | 97.1809 | 365997 |
| | Zhaoqing | 824.0969 | 48.6193 | 636466 |
| | Huizhou | 213.4754 | 72.8133 | 2740849 |
| | Meizhou | 5.1636 | 20.6545 | 415919 |
| | Shanwei | 36.5166 | 25.5616 | 247685 |
| | Heyuan | 236.1079 | 59.9080 | 582178 |
| | Yangjiang | 1367.6743 | 80.6774 | 342406 |
| | Qingyuan | 1133.6517 | 50.3845 | 691203 |
| | Dongguan | 448.0910 | 195.8607 | 7952214 |
| | Zhongshan | 4685.3144 | 355.3596 | 2584447 |
| | Chaozhou | 9321.0252 | 66.1894 | 285664 |

| | | | | |
|---|---|---|---|---|
| | Jieyang | 887.4444 | 69.9199 | 192787 |
| | Yunfu | 453.1437 | 37.7620 | 237092 |
| Guangxi | Nanning | 2071.7069 | 51.4781 | 2649152 |
| | Liuzhou | 9.6202 | 40.8857 | 1089338 |
| | Guilin | 32.4469 | 26.3631 | 825151 |
| | Wuzhou | 648.7114 | 14.1795 | 269501 |
| | Beihai | 253.6117 | 129.5038 | 427555 |
| | Fangchenggang | 1233.1894 | 28.6788 | 236640 |
| | Qinzhou | 60.5650 | 15.1412 | 286928 |
| | Guigang | 391.5425 | 9.2673 | 279057 |
| | Haerbin | 276.0160 | 8.6255 | 351047 |
| | Baise | 16.7996 | 13.9997 | 289935 |
| | Hezhou | 114.5499 | 9.9609 | 144806 |
| | Hechi | 8.7772 | 11.7029 | 253288 |
| | Laibin | 14.4605 | 9.6404 | 142351 |
| | Chongzuo | 62.2399 | 52.6645 | 247562 |
| Hainan | Haikou | 964.0288 | 156.6112 | 1399711 |
| | Sanya | 29.0868 | 38.7824 | 487004 |
| | Sansha | 0.0000 | 0.0000 | 1000 |
| | Danzhou | 1026.9749 | 282.9421 | 66458 |
| Chongqing | Chongqing | 442.6883 | 75.8092 | 6787107 |
| Sichuan | Chengdu | 7.6417 | 85.4915 | 8647616 |
| | Zigong | 40.1726 | 28.1209 | 320417 |
| | Panzhihua | 8.2494 | 32.9978 | 337457 |
| | Luzhou | 56.4155 | 35.2597 | 477176 |
| | Deyang | 8.6802 | 81.0147 | 470598 |
| | Mianyang | 4.1083 | 57.5156 | 972773 |

| | | | | |
|---|---|---|---|---|
| | Guangyuan | 4.3372 | 26.0230 | 279933 |
| | Suining | 7.1068 | 88.8353 | 282244 |
| | Neijiang | 6.3681 | 63.6805 | 312640 |
| | Leshan | 3.1644 | 63.2878 | 412809 |
| | Nanchong | 17.8331 | 51.7159 | 564115 |
| | Meishan | 6.7677 | 91.3638 | 348232 |
| | Yibin | 21.7922 | 28.3298 | 630559 |
| | Guangan | 43.0123 | 46.0846 | 203928 |
| | Dazhou | 5.5706 | 29.7098 | 564973 |
| | Yaan | 20.9117 | 41.8234 | 160976 |
| | Bazhong | 7.3722 | 22.1166 | 203149 |
| | Ziyang | 8.6631 | 64.9736 | 153048 |
| | Aba (Ngawa) Tibetan and Qiang Autonomous Prefecture | 0.0000 | 0.0000 | 102968 |
| | Ganzi (Garzê) Tibetan Autonomous Prefecture | 0.0000 | 0.0000 | 159147 |
| | Liangshan Yi Autonomous Prefecture | 2.0583 | 16.4665 | 524996 |
| Guizhou | Guiyang | 10.0217 | 16.7028 | 2606386 |
| | Liupanshui | 19.7915 | 6.5972 | 427666 |
| | Zunyi | 16.6498 | 22.7043 | 939983 |
| | Anshun | 12.1427 | 0.0000 | 257366 |
| | Bijie | 7.2468 | 4.3481 | 384760 |
| | Tongren | 18.1903 | 24.2537 | 325598 |
| | Qianxinan Buyi and Miao Autonomous Prefecture | 13.2665 | 13.2665 | 305700 |
| | Qiandongnan Miao and Dong Autonomous Prefecture | 0.0000 | 0.0000 | 388578 |
| | Qiannan Buyi and Miao Autonomous Prefecture | 11.4469 | 2.8617 | 386560 |
| Yunnan | Kunming | 101.6538 | 333.3299 | 3671687 |
| | Qujing | 27.7500 | 62.4374 | 515969 |
| | Yuxi | 75.5723 | 48.8997 | 305743 |

| | | | | |
|---|---|---|---|---|
| | Baoshan | 74.0372 | 316.7146 | 154327 |
| | Zhaotong | 23.5636 | 119.7814 | 284938 |
| | Lijiang | 7.9753 | 31.9010 | 199408 |
| | Pu’er | 536.3928 | 166.3233 | 329863 |
| | Lincang | 2661.6581 | 3392.3962 | 188238 |
| | Chuxiong Yi Autonomous Prefecture | 8.2756 | 49.6535 | 238740 |
| | Honghe Hani and Yi Autonomous Prefecture | 281.3491 | 46.8915 | 530953 |
| | Wenshan Zhuang and Miao Autonomous Prefecture | 5.7090 | 14.2726 | 276048 |
| | Dai Autonomous Prefecture of Xishuangbanna/Sipsongpanna | 61249.0942 | 5094.4862 | 375561 |
| | Dali Bai Autonomous Prefecture | 32.9582 | 83.8936 | 295572 |
| | Dehong Dai and Jingpo Autonomous Prefecture | 19670.0030 | 17944.6975 | 261760 |
| | Nujiang Lisu Autonomous Prefecture | 36.1864 | 18.0932 | 54714 |
| | Diqing Tibetan Autonomous Prefecture | 0.0000 | 0.0000 | 59221 |
| Tibet | Lhasa | 0.0000 | 0.0000 | 410693 |
| | Rikaze | 0.0000 | 0.0000 | 85619 |
| | Changdu | 0.0000 | 0.0000 | 65908 |
| | Linzhi | 0.0000 | 0.0000 | 76350 |
| | Shannan | 0.0000 | 0.0000 | 64397 |
| | Naqu | 0.0000 | 0.0000 | 44210 |
| | Ngari Prefecture | 0.0000 | 0.0000 | 30901 |
| Shaanxi | Xi’an | 4.9248 | 27.0863 | 4248263 |
| | Tongchuan | 0.0000 | 14.3200 | 91942 |
| | Baoji | 3.0104 | 15.0518 | 419769 |
| | Xianyang | 2.0067 | 10.0334 | 740049 |
| | Weinan | 4.2655 | 19.1949 | 221361 |
| | Yan’an | 0.0000 | 8.7620 | 337527 |
| | Hanzhong | 3.1138 | 3.1138 | 223156 |

| | | | | |
|---|---|---|---|---|
| | Yulin | 0.0000 | 0.0000 | 816544 |
| | Ankang | 4.0105 | 4.0105 | 126243 |
| | Shangluo | 4.8990 | 9.7980 | 71955 |
| Gansu | Lanzhou | 2.2939 | 9.1755 | 1737675 |
| | Jiayuguan | 63.9666 | 0.0000 | 127329 |
| | Jinchang | 0.0000 | 0.0000 | 84697 |
| | Baiyin | 0.0000 | 6.6133 | 171743 |
| | Tianshui | 0.0000 | 13.4019 | 155290 |
| | Wuwei | 0.0000 | 0.0000 | 93902 |
| | Zhangye | 0.0000 | 8.8416 | 89978 |
| | Pingliang | 5.4095 | 0.0000 | 132070 |
| | Jiuquan | 0.0000 | 0.0000 | 222553 |
| | Qingyang | 0.0000 | 0.0000 | 241209 |
| | Dingxi | 0.0000 | 0.0000 | 114339 |
| | Longnan | 4.1541 | 16.6163 | 97486 |
| | Linxia Hui Autonomous Prefecture | 0.0000 | 14.2197 | 150960 |
| | Gannan Tibetan Autonomous Prefecture | 0.0000 | 0.0000 | 69033 |
| Qinghai | Xining | 4.0519 | 0.0000 | 766358 |
| | Haidong | 0.0000 | 0.0000 | 92301 |
| | Haibei Tibetan Autonomous Prefecture | 0.0000 | 0.0000 | 30530 |
| | Huangnan Tibetan Autonomous Prefecture | 0.0000 | 0.0000 | 30277 |
| | Hainan Tibetan Autonomous Prefecture | 0.0000 | 0.0000 | 51569 |
| | Guoluo (Golog) Tibetan Autonomous Prefecture | 0.0000 | 0.0000 | 29207 |
| | Yushu Tibetan Autonomous Prefecture | 0.0000 | 0.0000 | 32736 |
| | Haixi Mongolian and Tibetan Autonomous Prefecture | 0.0000 | 0.0000 | 206584 |
| Ningxia | Yinchuan | 3.4976 | 17.4882 | 1210130 |
| | Shizuishan | 0.0000 | 0.0000 | 171268 |

| | | | | |
|---|---|---|---|---|
| | Wuzhong | 7.2322 | 0.0000 | 243275 |
| | Guyuan | 8.7555 | 0.0000 | 122856 |
| | Zhongwei | 0.0000 | 0.0000 | 130332 |
| Xinjiang | Urumqi | 0.0000 | 2.4665 | 2140827 |
| | Karamay | 0.0000 | 0.0000 | 190754 |
| | Tulufan | 0.0000 | 0.0000 | 115855 |
| | Hami | 0.0000 | 0.0000 | 199976 |
| | Changji Hui Autonomous Prefecture | 0.0000 | 0.0000 | 477313 |
| | Bortala Mongolian Autonomous Prefecture | 0.0000 | 0.0000 | 105934 |
| | Bayingol Mongolian Autonomous Prefecture | 0.0000 | 6.6259 | 444888 |
| | Aksu Prefecture | 0.0000 | 0.0000 | 456769 |
| | Kizilsu Kirgiz Autonomous Prefecture | 0.0000 | 16.0714 | 53345 |
| | Kashgar Prefecture | 0.0000 | 0.0000 | 329258 |
| | Hotan Prefecture | 4.0963 | 0.0000 | 186809 |
| | Ili Kazakh Autonomous Prefecture | 0.0000 | 0.0000 | 441260 |
| | Tarbagatay Prefecture | 0.0000 | 0.0000 | 148078 |
| | Altay Prefecture | 0.0000 | 0.0000 | 102148 |

**Supplementary Table 4. Transmission model and parameters estimation results.** Estimated total infections include reported infections and unreported infections for a full year in each city. The average of daily results include transmission rate, effective reproduction number and force of infection in September and October. The set median and 95% CIs are displayed outside and inside the brackets, respectively.

| Province | City | Estimated total infection | Transmission rate | Effective reproduction number | Force of infection |
|---|---|---|---|---|---|
| Beijing | Beijing | 3 (1, 7) | 0.0253 (0.0226, 0.0282) | 0.0431 (0.0383, 0.0482) | 1.1x10^-15 (9.9x10^-16, 5.1x10^-13) |
| Tianjin | Tianjin | 2 (0, 4) | 0.0308 (0.0272, 0.0345) | 0.0384 (0.0338, 0.0433) | 1.7x10^-15 (1.5x10^-15, 4.9x10^-13) |
| Hebei | Shijiazhuang | 1 (0, 4) | 0.0302 (0.0268, 0.034) | 0.0563 (0.0497, 0.0638) | 4.9x10^-15 (4.3x10^-15, 2.6x10^-12) |
| | Tangshan | 1 (0, 3) | 0.0271 (0.0241, 0.0307) | 0.0474 (0.0417, 0.0541) | 1.9x10^-15 (1.7x10^-15, 3.5x10^-14) |
| | Qinghuangdao | 1 (0, 3) | 0.0246 (0.0215, 0.0275) | 0.0418 (0.036, 0.0473) | 6.1x10^-17 (5.3x10^-17, 2.9x10^-14) |
| | Handan | 2 (0, 4) | 0.0294 (0.0258, 0.0332) | 0.0562 (0.0489, 0.064) | 4.8x10^-15 (4.2x10^-15, 1.0x10^-12) |
| | Xingtai | 1 (0, 4) | 0.0286 (0.0253, 0.032) | 0.0524 (0.0459, 0.0589) | 2.3x10^-14 (2.0x10^-14, |

| | | | | | |
|---|---|---|---|---|---|
| | | | | | 1.8x10^-11) |
| | Baoding | 1 (0, 5) | 0.0251 (0.0221, 0.0282) | 0.0432 (0.0378, 0.0489) | 4.0x10^-16 (3.5x10^-16, 1.3x10^-13) |
| | Zhangjiakou | 0 (0, 2) | 0.0107 (0.0094, 0.012) | 0.018 (0.0157, 0.0204) | 0 (0, 0) |
| | Chengde | 0 (0, 1) | 0.0131 (0.0116, 0.0148) | 0.0226 (0.0197, 0.0256) | 5.1x10^-15 (4.5x10^-15, 7.8x10^-13) |
| | Cangzhou | 1 (0, 4) | 0.0282 (0.025, 0.0316) | 0.0482 (0.0424, 0.0546) | 5.2x10^-15 (4.5x10^-15, 2.0x10^-12) |
| | Langfang | 1 (0, 2) | 0.0262 (0.0231, 0.0295) | 0.0364 (0.0319, 0.0415) | 9.8x10^-15 (8.7x10^-15, 7.0x10^-12) |
| | Hengshui | 1 (0, 3) | 0.0291 (0.0259, 0.0328) | 0.0515 (0.0453, 0.0586) | 4.6x10^-14 (4.1x10^-14, 3.2x10^-11) |
| Shanxi | Taiyuan | 0 (0, 2) | 0.0128 (0.0113, 0.0144) | 0.0091 (0.0078, 0.0104) | 0 (0, 0) |
| | Datong | 0 (0, 1) | 0.0094 (0.0083, 0.0104) | 0.0067 (0.0058, 0.0077) | 0 (0, 0) |
| | Yangquan | 0 (0, 1) | 0.0155 (0.0137, 0.0175) | 0.0106 (0.009, 0.0124) | 0 (0, 0) |
| | Changzhi | 0 (0, 1) | 0.0118 (0.0105, 0.0131) | 0.0086 (0.0075, 0.0098) | 0 (0, 0) |
| | Jincheng | 0 (0, 1) | 0.0143 (0.0127, | 0.0104 (0.009, | 0 (0, 0) |

| | | | | | |
|---|---|---|---|---|---|
| | | | 0.0162) | 0.012) | |
| | Shuozhou | 0 (0, 1) | 0.0096 (0.0085, 0.0107) | 0.0065 (0.0056, 0.0075) | 0 (0, 0) |
| | Jinzhong | 0 (0, 3) | 0.012 (0.0106, 0.0135) | 0.0076 (0.0065, 0.0087) | 0 (0, 0) |
| | Yuncheng | 1 (0, 3) | 0.0229 (0.0203, 0.0258) | 0.0172 (0.0149, 0.0197) | 0 (0, 0) |
| | Xinzhou | 0 (0, 2) | 0.0081 (0.0072, 0.0091) | 0.0056 (0.0048, 0.0065) | 0 (0, 0) |
| | Linfen | 0 (0, 3) | 0.017 (0.015, 0.0191) | 0.0124 (0.0108, 0.0142) | 0 (0, 0) |
| | Lvliang | 0 (0, 2) | 0.0118 (0.0103, 0.0132) | 0.0084 (0.0072, 0.0097) | 0 (0, 0) |
| Inner Mongolia | Hohhot | 0 (0, 1) | 0.0095 (0.0084, 0.0107) | 0.0303 (0.0266, 0.0342) | 2.3x10^-15 (2.0x10^-15, 2.9x10^-14) |
| | Baotou | 0 (0, 1) | 0.0082 (0.0072, 0.0092) | 0.0263 (0.023, 0.0298) | 8.1x10^-16 (7.2x10^-16, 9.2x10^-13) |
| | Wuhai | 0 (0, 1) | 0.0168 (0.015, 0.0187) | 0.0509 (0.0445, 0.0575) | 0 (0, 0) |
| | Chifeng | 0 (0, 1) | 0.0105 (0.0093, 0.0119) | 0.035 (0.0306, 0.0396) | 3.4x10^-15 (3.0x10^-15, 5.9x10^-10) |
| | Tongliao | 0 (0, 1) | 0.0126 (0.0111, 0.0141) | 0.0403 (0.0355, 0.0457) | 1.1x10^-15 (1.0x10^-15, 1.3x10^-12) |

| | | | | | |
|---|---|---|---|---|---|
| | Ordos | 0 (0, 2) | 0.0094 (0.0082, 0.0104) | 0.0269 (0.0234, 0.0302) | 2.2x10^-15 (1.9x10^-15, 7.6x10^-13) |
| | HulunBuir | 0 (0, 1) | 0.0029 (0.0026, 0.0033) | 0.0099 (0.0087, 0.0113) | 0 (0, 0) |
| | BayanNur | 0 (0, 1) | 0.0131 (0.0116, 0.0148) | 0.0421 (0.037, 0.0481) | 5.1x10^-15 (4.5x10^-15, 5.7x10^-15) |
| | Ulanqab | 0 (0, 1) | 0.0048 (0.0042, 0.0053) | 0.0141 (0.0124, 0.0158) | 0 (0, 0) |
| | Hinggan League | 0 (0, 1) | 0.008 (0.0071, 0.0089) | 0.0258 (0.0227, 0.029) | 0 (0, 0) |
| | Xilingol League | 0 (0, 1) | 0.0076 (0.0067, 0.0084) | 0.0239 (0.021, 0.0269) | 0 (0, 0) |
| | Alxa League | 0 (0, 1) | 0.017 (0.0151, 0.0189) | 0.0421 (0.0365, 0.0479) | 2.8x10^-14 (2.5x10^-14, 3.1x10^-11) |
| Liaoning | Shenyang | 0 (0, 3) | 0.0158 (0.014, 0.0178) | 0.0268 (0.0236, 0.0304) | 0 (0, 0) |
| | Dalian | 0 (0, 1) | 0.0231 (0.0205, 0.0257) | 0.0422 (0.0372, 0.0472) | 3.4x10^-15 (3.0x10^-15, 7.1x10^-11) |
| | Anshan | 0 (0, 2) | 0.0174 (0.0154, 0.0197) | 0.0288 (0.0253, 0.0328) | 0 (0, 0) |
| | Fushun | 0 (0, 1) | 0.0092 (0.0082, 0.0102) | 0.0148 (0.0131, 0.0167) | 0 (0, 0) |
| | Benxi | 0 (0, 0) | 0.0121 (0.0106, | 0.0201 (0.0174, | 0 (0, 0) |

| | | | | | |
|---|---|---|---|---|---|
| | | | 0.0135) | 0.0228) | |
| | Dandong | 0 (0, 1) | 0.0171 (0.0152, 0.019) | 0.0294 (0.0259, 0.0331) | 0 (0, 0) |
| | Jinzhou | 0 (0, 1) | 0.0185 (0.0163, 0.0208) | 0.0303 (0.0264, 0.0345) | 8.0x10^-15 (7.0x10^-15, 4.0x10^-14) |
| | Yingkou | 0 (0, 1) | 0.0188 (0.0166, 0.0211) | 0.0295 (0.0257, 0.0335) | 1.8x10^-15 (1.5x10^-15, 2.0x10^-15) |
| | Fuxin | 0 (0, 1) | 0.0146 (0.0129, 0.0164) | 0.0245 (0.0215, 0.0279) | 0 (0, 0) |
| | Liaoyang | 0 (0, 1) | 0.0183 (0.0162, 0.0206) | 0.0287 (0.0252, 0.0328) | 1.3x10^-15 (1.1x10^-15, 1.4x10^-15) |
| | Panjin | 0 (0, 2) | 0.0189 (0.0167, 0.021) | 0.0286 (0.025, 0.0323) | 0 (0, 0) |
| | Tieling | 0 (0, 1) | 0.0132 (0.0117, 0.0148) | 0.0216 (0.0189, 0.0245) | 2.6x10^-15 (2.3x10^-15, 3.0x10^-12) |
| | Chaoyang | 0 (0, 1) | 0.015 (0.0133, 0.0169) | 0.0266 (0.0233, 0.0303) | 0 (0, 0) |
| | Huludao | 1 (0, 3) | 0.0184 (0.0163, 0.0207) | 0.0298 (0.0261, 0.0338) | 0 (0, 0) |
| Jilin | Changchun | 0 (0, 2) | 0.0103 (0.0091, 0.0117) | 0.0334 (0.0293, 0.0378) | 0 (0, 0) |
| | Jilin | 0 (0, 1) | 0.0088 (0.0078, 0.0099) | 0.0279 (0.0245, 0.0315) | 1.6x10^-16 (1.4x10^-16, |

| | | | | | |
|---|---|---|---|---|---|
| | | | | | 1.8x10^-16) |
| | Siping | 0 (0, 2) | 0.0125 (0.0111, 0.014) | 0.0326 (0.0286, 0.037) | 2.4x10^-15 (2.2x10^-15, 2.6x10^-13) |
| | Liaoyuan | 0 (0, 1) | 0.008 (0.0071, 0.0091) | 0.0253 (0.0222, 0.0291) | 1.6x10^-15 (1.5x10^-15, 1.9x10^-15) |
| | Tonghua | 0 (0, 1) | 0.0092 (0.0081, 0.0103) | 0.0291 (0.0254, 0.0331) | 0 (0, 0) |
| | Baishan | 0 (0, 1) | 0.0035 (0.0031, 0.0038) | 0.011 (0.0097, 0.0123) | 0 (0, 0) |
| | Songyuan | 0 (0, 1) | 0.0121 (0.0107, 0.0136) | 0.0354 (0.0311, 0.0401) | 1.1x10^-15 (1.0x10^-15, 1.3x10^-15) |
| | Baicheng | 0 (0, 1) | 0.0117 (0.0104, 0.013) | 0.0377 (0.0331, 0.0422) | 0 (0, 0) |
| | Yanbian Chaoxianzu (Korean) Autonomous Prefecture | 0 (0, 1) | 0.0074 (0.0066, 0.0082) | 0.0244 (0.0215, 0.0274) | 0 (0, 0) |
| Heilongjiang | Harbin | 0 (0, 3) | 0.0074 (0.0066, 0.0084) | 0.0242 (0.0214, 0.0275) | 4.2x10^-15 (3.7x10^-15, 3.9x10^-12) |
| | Qiqihar | 0 (0, 1) | 0.0068 (0.0061, 0.0076) | 0.0225 (0.0198, 0.0251) | 1.4x10^-15 (1.2x10^-15, 1.0x10^-12) |
| | Jixi | 0 (0, 1) | 0.0075 (0.0067, 0.0084) | 0.0248 (0.0218, 0.0279) | 0 (0, 0) |
| | Hegang | 0 (0, 0) | 0.0057 (0.005, | 0.0184 (0.0162, | 0 (0, 0) |

| | | | | | |
|---|---|---|---|---|---|
| | | | 0.0063) | 0.0208) | |
| | Shuangyashan | 0 (0, 1) | 0.0076 (0.0067, 0.0084) | 0.0241 (0.0211, 0.027) | 0 (0, 0) |
| | Daqing | 0 (0, 1) | 0.0105 (0.0092, 0.0118) | 0.0332 (0.0291, 0.0377) | 0 (0, 0) |
| | Yichun | 0 (0, 0) | 0.0039 (0.0035, 0.0044) | 0.0128 (0.0112, 0.0144) | 0 (0, 0) |
| | Jiamusi | 0 (0, 1) | 0.0068 (0.0061, 0.0077) | 0.0218 (0.0191, 0.0247) | 0 (0, 0) |
| | Qitaihe | 0 (0, 1) | 0.0078 (0.0069, 0.0087) | 0.0247 (0.0217, 0.0279) | 0 (0, 0) |
| | Mudanjiang | 0 (0, 1) | 0.0067 (0.006, 0.0075) | 0.0219 (0.0193, 0.0246) | 0 (0, 0) |
| | Heihe | 0 (0, 1) | 0.0043 (0.0038, 0.0048) | 0.0141 (0.0124, 0.0158) | 6.2x10^-17 (5.5x10^-17, 6.9x10^-17) |
| | Suihua | 0 (0, 1) | 0.0067 (0.0059, 0.0076) | 0.0204 (0.0179, 0.0232) | 0 (0, 0) |
| | Da Hinggan Ling Prefecture | 0 (0, 0) | 0.0013 (0.0011, 0.0014) | 0.0043 (0.0038, 0.0049) | 0 (0, 0) |
| Shanghai | Shanghai | 5 (2, 9) | 0.0467 (0.0417, 0.0534) | 0.5682 (0.5058, 0.6492) | 1.0x10^-13 (8.9x10^-14, 2.1x10^-9) |
| Jiangsu | Nanjing | 3 (1, 8) | 0.0424 (0.0379, 0.0476) | 0.6482 (0.5801, 0.7292) | 2.5x10^-13 (2.3x10^-13, 5.3x10^-9) |
| | Wuxi | 2 (0, 6) | 0.0452 (0.0399, | 0.6294 (0.5547, | 2.5x10^-13 |

| | | | | |
|---|---|---|---|---|
| | | 0.0506) | 0.7047) | (2.2x10^-13, 6.5x10^-9) |
| Xuzhou | 1 (0, 3) | 0.0346 (0.0306, 0.0391) | 0.5556 (0.4916, 0.6285) | 9.9x10^-14 (8.8x10^-14, 4.0x10^-9) |
| Changzhou | 2 (1, 6) | 0.0443 (0.04, 0.0496) | 0.6353 (0.5738, 0.7122) | 1.8x10^-13 (1.6x10^-13, 7.4x10^-9) |
| Suzhou | 5 (1, 10) | 0.0479 (0.0421, 0.0543) | 0.6437 (0.5655, 0.7304) | 1.3x10^-13 (1.2x10^-13, 2.7x10^-9) |
| Nantong | 2 (0, 4) | 0.042 (0.037, 0.047) | 0.6493 (0.5712, 0.7283) | 2.4x10^-13 (2.1x10^-13, 6.7x10^-9) |
| Lianyungang | 1 (0, 4) | 0.0374 (0.0331, 0.0422) | 0.588 (0.5189, 0.6636) | 1.1x10^-13 (1.0x10^-13, 7.1x10^-9) |
| Huaian | 1 (0, 5) | 0.0365 (0.0322, 0.041) | 0.5587 (0.4917, 0.6291) | 1.3x10^-13 (1.1x10^-13, 8.6x10^-9) |
| Yancheng | 1 (0, 5) | 0.0373 (0.0329, 0.042) | 0.5891 (0.5198, 0.6653) | 1.1x10^-13 (1.0x10^-13, 6.1x10^-9) |
| Yangzhou | 1 (0, 3) | 0.0397 (0.0351, 0.0444) | 0.5962 (0.5272, 0.6684) | 1.0x10^-10 (9.2x10^-11, 1.2x10^-8) |
| Zhenjiang | 0 (0, 7) | 0.0435 (0.0386, | 0.6147 (0.5445, | 2.1x10^-13 |

| | | | | | |
|---|---|---|---|---|---|
| | | | 0.0491) | 0.6941) | (1.9x10^-13, 1.6x10^-8) |
| | Taizhou | 0 (0, 4) | 0.0417 (0.0368, 0.0469) | 0.6232 (0.5481, 0.7016) | 2.8x10^-13 (2.5x10^-13, 1.1x10^-8) |
| | Suqian | 0 (0, 3) | 0.0363 (0.032, 0.041) | 0.5744 (0.5065, 0.6501) | 3.3x10^-14 (2.9x10^-14, 5.8x10^-9) |
| Zhejiang | Hangzhou | 34 (24, 52) | 0.0533 (0.0478, 0.06) | 1.2886 (1.1556, 1.452) | 1.1x10^-8 (4.0x10^-9, 3.9x10^-8) |
| | Ningbo | 8 (4, 16) | 0.0547 (0.0493, 0.0604) | 1.4166 (1.2767, 1.5668) | 5.1x10^-10 (2.8x10^-10, 2.3x10^-8) |
| | Wenzhou | 732 (571, 1004) | 0.0545 (0.0494, 0.0605) | 1.4646 (1.327, 1.6244) | 1.2x10^-6 (7.6x10^-7, 2.0x10^-6) |
| | Jiaxing | 9 (4, 14) | 0.0459 (0.0416, 0.0514) | 0.9719 (0.8791, 1.088) | 1.8x10^-10 (8.0x10^-11, 1.0x10^-8) |
| | Huzhou | 5 (2, 9) | 0.0452 (0.0402, 0.0506) | 1.0008 (0.8894, 1.1234) | 1.3x10^-10 (1.1x10^-10, 1.6x10^-8) |
| | Shaoxing | 7 (3, 14) | 0.0472 (0.0424, 0.0531) | 1.0538 (0.9456, 1.1856) | 4.3x10^-11 (3.9x10^-11, 1.0x10^-8) |
| | Jinhua | 9 (4, 19) | 0.0513 (0.0457, | 1.3062 (1.1623, | 2.4x10^-9 |

| | | | | | |
|---|---|---|---|---|---|
| | | | 0.0577) | 1.4714) | (2.3x10^-10, 3.2x10^-8) |
| | Quzhou | 6 (2, 12) | 0.0534 (0.0474, 0.0596) | 1.265 (1.1202, 1.4158) | 1.3x10^-10 (7.9x10^-11, 4.4x10^-8) |
| | Zhoushan | 0 (0, 5) | 0.049 (0.0435, 0.055) | 1.1937 (1.0559, 1.344) | 8.8x10^-13 (7.8x10^-13, 7.4x10^-8) |
| | Taizhou | 21 (13, 38) | 0.05 (0.0439, 0.0557) | 1.2673 (1.1119, 1.4155) | 3.2x10^-9 (1.4x10^-10, 3.6x10^-8) |
| | Lishui | 9 (2, 17) | 0.0542 (0.0481, 0.0606) | 1.3579 (1.2034, 1.5236) | 1.8x10^-9 (1.1x10^-9, 5.2x10^-8) |
| Anhui | Hefei | 4 (1, 9) | 0.0404 (0.0361, 0.045) | 0.9677 (0.8664, 1.0778) | 2.7x10^-11 (4.3x10^-13, 9.3x10^-9) |
| | Wuhu | 1 (0, 6) | 0.0443 (0.0389, 0.0501) | 1.055 (0.9244, 1.1958) | 3.3x10^-13 (2.9x10^-13, 2.4x10^-8) |
| | Bengbu | 1 (0, 4) | 0.0365 (0.0323, 0.0409) | 0.8716 (0.7688, 0.979) | 1.0x10^-13 (8.9x10^-14, 2.1x10^-8) |
| | Huainan | 0 (0, 5) | 0.0398 (0.0351, 0.0449) | 0.949 (0.8366, 1.0724) | 1.0x10^-13 (9.4x10^-14, 2.4x10^-8) |
| | Maanshan | 1 (0, 6) | 0.0442 (0.039, | 0.9777 (0.8607, | 6.4x10^-13 |

| | | | | |
|---|---|---|---|---|
| | | 0.0496) | 1.1002) | (5.6x10^-13, 3.0x10^-8) |
| Huaibei | 0 (0, 2) | 0.0369 (0.0325, 0.0415) | 0.9032 (0.7948, 1.0175) | 4.3x10^-14 (3.7x10^-14, 1.7x10^-8) |
| Tongling | 1 (0, 6) | 0.0461 (0.041, 0.052) | 1.0823 (0.9594, 1.2254) | 6.2x10^-13 (5.5x10^-13, 7.3x10^-8) |
| Anqing | 3 (0, 10) | 0.045 (0.0401, 0.0509) | 1.1028 (0.9824, 1.251) | 5.1x10^-13 (4.6x10^-13, 2.3x10^-8) |
| Huangshan | 1 (0, 5) | 0.0398 (0.0354, 0.045) | 0.9374 (0.8323, 1.0647) | 1.0x10^-12 (9.0x10^-13, 4.6x10^-8) |
| Chuzhou | 1 (0, 7) | 0.038 (0.0335, 0.0425) | 0.8407 (0.7387, 0.9406) | 6.2x10^-13 (5.4x10^-13, 2.0x10^-8) |
| Fuyang | 1 (0, 4) | 0.0387 (0.0343, 0.0437) | 0.9694 (0.8581, 1.0976) | 1.6x10^-13 (1.4x10^-13, 8.6x10^-9) |
| Suzhou | 1 (0, 3) | 0.0345 (0.0306, 0.0389) | 0.8354 (0.7408, 0.9439) | 1.7x10^-13 (1.5x10^-13, 8.9x10^-9) |
| Lu'an | 2 (0, 7) | 0.0386 (0.034, 0.0435) | 0.9056 (0.7956, 1.022) | 4.4x10^-13 (3.9x10^-13, 2.0x10^-8) |
| Bozhou | 1 (0, 4) | 0.04 (0.0357, | 0.9884 (0.8806, | 2.9x10^-13 |

| | | | | | |
|---|---|---|---|---|---|
| | | | 0.0448) | 1.1088) | (2.6x10^-13, 1.6x10^-8) |
| | Chizhou | 2 (0, 7) | 0.0443 (0.039, 0.0495) | 1.0103 (0.8871, 1.1297) | 8.6x10^-10 (1.9x10^-12, 5.9x10^-8) |
| | Xuancheng | 1 (0, 4) | 0.0426 (0.0377, 0.048) | 0.982 (0.8675, 1.1103) | 2.5x10^-13 (2.2x10^-13, 2.4x10^-8) |
| Fujian | Fuzhou | 1859 (1504, 2386) | 0.0658 (0.0606, 0.0723) | 1.5634 (1.4384, 1.7184) | 3.1x10^-6 (2.1x10^-6, 4.7x10^-6) |
| | Amoy | 92 (64, 141) | 0.0668 (0.0607, 0.076) | 1.4608 (1.3254, 1.6657) | 1.2x10^-7 (5.0x10^-8, 3.1x10^-7) |
| | Putian | 1297 (1017, 1685) | 0.0683 (0.0615, 0.0749) | 1.5144 (1.3595, 1.6634) | 4.4x10^-6 (2.7x10^-6, 6.9x10^-6) |
| | Sanming | 58 (44, 81) | 0.0601 (0.0539, 0.0674) | 1.3484 (1.2065, 1.5163) | 1.1x10^-7 (3.4x10^-8, 3.1x10^-7) |
| | Quanzhou | 332 (254, 436) | 0.0537 (0.0501, 0.061) | 1.2133 (1.1293, 1.3795) | 4.5x10^-7 (2.6x10^-7, 7.7x10^-7) |
| | Zhangzhou | 341 (266, 485) | 0.0645 (0.0592, 0.073) | 1.4019 (1.2833, 1.5872) | 6.4x10^-7 (3.5x10^-7, 1.1x10^-6) |
| | Nanping | 42 (30, 59) | 0.0578 (0.0529, | 1.3206 (1.2039, | 6.4x10^-8 |

| | | | | | |
|---|---|---|---|---|---|
| | | | 0.0677) | 1.5502) | (1.3x10^-8, 2.3x10^-7) |
| | Longyan | 7 (3, 15) | 0.0592 (0.0527, 0.0665) | 1.3868 (1.2306, 1.5639) | 6.8x10^-10 (1.9x10^-12, 6.1x10^-8) |
| | Ningde | 193 (146, 279) | 0.0504 (0.0455, 0.0565) | 1.1199 (1.0087, 1.2567) | 7.0x10^-7 (3.1x10^-7, 1.3x10^-6) |
| Jiangxi | Nanchang | 172 (130, 224) | 0.0573 (0.0515, 0.0659) | 0.3703 (0.3321, 0.4279) | 3.6x10^-7 (1.9x10^-7, 6.4x10^-7) |
| | Jingdezhen | 3 (1, 6) | 0.0561 (0.0506, 0.0625) | 0.3546 (0.3178, 0.3973) | 6.0x10^-10 (5.4x10^-10, 1.3x10^-8) |
| | Pingxiang | 3 (0, 8) | 0.0527 (0.0468, 0.0591) | 0.3419 (0.3025, 0.3846) | 8.7x10^-13 (7.7x10^-13, 1.8x10^-8) |
| | Jiujiang | 7 (2, 12) | 0.0502 (0.0447, 0.0561) | 0.3194 (0.2835, 0.3578) | 3.9x10^-13 (3.5x10^-13, 8.6x10^-9) |
| | Xinyu | 3 (0, 7) | 0.0563 (0.0498, 0.0635) | 0.3591 (0.3167, 0.4073) | 1.1x10^-12 (9.8x10^-13, 3.2x10^-8) |
| | Yingtan | 2 (0, 7) | 0.0566 (0.05, 0.0645) | 0.3519 (0.3091, 0.4023) | 1.6x10^-12 (1.4x10^-12, 4.2x10^-8) |
| | Ganzhou | 194 (147, | 0.0572 (0.0525, | 0.3719 (0.3407, | 2.8x10^-7 |

| | | | | | |
|---|---|---|---|---|---|
| | | 286) | 0.0641) | 0.4187) | (1.6x10^-7, 5.0x10^-7) |
| | Jian | 361 (287, 452) | 0.0655 (0.0592, 0.0724) | 0.4237 (0.3812, 0.47) | 8.7x10^-7 (6.2x10^-7, 1.2x10^-6) |
| | Yichun | 523 (447, 639) | 0.0699 (0.0618, 0.0745) | 0.4406 (0.3882, 0.4703) | 7.8x10^-7 (5.3x10^-7, 1.0x10^-6) |
| | Fuzhou | 10 (5, 15) | 0.055 (0.0496, 0.0622) | 0.3427 (0.3075, 0.3897) | 4.0x10^-11 (1.0x10^-11, 1.0x10^-8) |
| | Shangrao | 8 (2, 14) | 0.0543 (0.0493, 0.0619) | 0.3483 (0.3153, 0.3991) | 4.2x10^-13 (3.8x10^-13, 6.0x10^-9) |
| Shandong | Jinan | 1 (0, 4) | 0.0309 (0.0273, 0.0347) | 0.0404 (0.0354, 0.0456) | 1.5x10^-15 (1.3x10^-15, 7.4x10^-13) |
| | Qingdao | 1 (0, 4) | 0.0332 (0.0292, 0.0373) | 0.0439 (0.0384, 0.0497) | 0 (0, 0) |
| | Zibo | 0 (0, 2) | 0.0293 (0.0261, 0.033) | 0.0377 (0.0332, 0.0429) | 0 (0, 0) |
| | Zaozhuang | 0 (0, 2) | 0.0366 (0.0323, 0.0412) | 0.0498 (0.0435, 0.0565) | 0 (0, 0) |
| | Dongying | 0 (0, 1) | 0.0319 (0.0283, 0.0358) | 0.0396 (0.0346, 0.0451) | 2.8x10^-14 (2.5x10^-14, 3.1x10^-9) |
| | Yantai | 0 (0, 2) | 0.0301 (0.0267, | 0.0396 (0.0348, | 0 (0, 0) |

| | | | | |
|---|---|---|---|---|
| | | 0.0339) | 0.0449) | |
| Weifang | 1 (0, 3) | 0.0294 (0.0259, 0.0332) | 0.0377 (0.0331, 0.0429) | 0 (0, 0) |
| Jining | 1 (0, 4) | 0.0339 (0.0298, 0.0381) | 0.0458 (0.04, 0.0518) | 6.5x10^-15 (5.7x10^-15, 1.1x10^-12) |
| Taian | 0 (0, 2) | 0.0241 (0.0213, 0.0271) | 0.0313 (0.0274, 0.0354) | 0 (0, 0) |
| Weihai | 0 (0, 1) | 0.0321 (0.0284, 0.0361) | 0.0432 (0.0377, 0.0492) | 8.7x10^-15 (7.7x10^-15, 2.9x10^-12) |
| Rizhao | 0 (0, 1) | 0.0328 (0.029, 0.0369) | 0.0419 (0.0367, 0.0478) | 8.5x10^-16 (7.5x10^-16, 9.5x10^-16) |
| Linyi | 1 (0, 4) | 0.0332 (0.0292, 0.0372) | 0.0443 (0.0387, 0.05) | 3.8x10^-15 (3.3x10^-15, 1.4x10^-12) |
| Dezhou | 1 (0, 2) | 0.0283 (0.0251, 0.032) | 0.0359 (0.0316, 0.0409) | 6.2x10^-15 (5.5x10^-15, 2.8x10^-12) |
| Liaocheng | 1 (0, 4) | 0.0274 (0.0243, 0.031) | 0.0363 (0.0319, 0.0415) | 8.5x10^-15 (7.5x10^-15, 1.0x10^-12) |
| Binzhou | 0 (0, 2) | 0.0306 (0.0271, 0.0345) | 0.0378 (0.0332, 0.0432) | 0 (0, 0) |
| Heze | 2 (0, 6) | 0.0349 (0.0308, 0.0392) | 0.0477 (0.0419, 0.054) | 3.7x10^-14 (3.2x10^-14, |

| | | | | | |
|---|---|---|---|---|---|
| | | | | | 3.0x10^-11) |
| Henan | Zhengzhou | 4 (1, 8) | 0.0319 (0.0281, 0.0363) | 0.6705 (0.5892, 0.7636) | 2.3x10^-11 (2.0x10^-11, 5.3x10^-9) |
| | Kaifeng | 2 (0, 7) | 0.0345 (0.0304, 0.0387) | 0.6962 (0.6122, 0.7826) | 3.2x10^-13 (2.8x10^-13, 8.5x10^-9) |
| | Luoyang | 2 (1, 5) | 0.0281 (0.0251, 0.0314) | 0.595 (0.5296, 0.6641) | 3.4x10^-10 (3.0x10^-10, 5.2x10^-9) |
| | Pingdingshan | 2 (0, 10) | 0.0343 (0.0301, 0.0386) | 0.728 (0.6369, 0.8207) | 2.9x10^-10 (5.9x10^-13, 1.7x10^-8) |
| | Anyang | 2 (0, 4) | 0.0303 (0.0264, 0.0344) | 0.6429 (0.56, 0.7314) | 1.3x10^-13 (1.1x10^-13, 6.2x10^-9) |
| | Hebi | 0 (0, 3) | 0.0307 (0.027, 0.0349) | 0.6355 (0.5574, 0.7275) | 2.0x10^-13 (1.8x10^-13, 2.1x10^-8) |
| | Xinxiang | 2 (0, 6) | 0.0329 (0.0292, 0.037) | 0.6789 (0.6026, 0.765) | 3.0x10^-13 (2.6x10^-13, 8.2x10^-9) |
| | Jiaozuo | 0 (0, 5) | 0.0336 (0.0297, 0.0378) | 0.6943 (0.6116, 0.7847) | 1.5x10^-13 (1.4x10^-13, 1.5x10^-8) |
| | Puyang | 143 (105, 195) | 0.0294 (0.0261, 0.033) | 0.6171 (0.5478, 0.6934) | 4.5x10^-8 (1.0x10^-8, |

| | | | | | |
|---|---|---|---|---|---|
| | | | | | 1.4x10^-7) |
| | Xuchang | 2 (0, 6) | 0.0329 (0.029, 0.0369) | 0.6719 (0.591, 0.7546) | 4.3x10^-13 (3.8x10^-13, 1.0x10^-8) |
| | Luohe | 1 (0, 5) | 0.0331 (0.0293, 0.0371) | 0.6982 (0.6151, 0.7832) | 2.9x10^-13 (2.6x10^-13, 2.8x10^-8) |
| | Sanmenxia | 1 (0, 3) | 0.0203 (0.018, 0.0229) | 0.3957 (0.3497, 0.4477) | 5.3x10^-13 (4.7x10^-13, 1.6x10^-8) |
| | Nanyang | 5 (2, 10) | 0.036 (0.0318, 0.0409) | 0.7838 (0.692, 0.8919) | 3.1x10^-13 (2.7x10^-13, 6.0x10^-9) |
| | Shangqiu | 1 (0, 4) | 0.0324 (0.0285, 0.0368) | 0.7007 (0.6155, 0.7968) | 1.2x10^-11 (1.0x10^-11, 6.9x10^-9) |
| | Xinyang | 1 (0, 6) | 0.0411 (0.0366, 0.0468) | 0.8985 (0.7992, 1.0271) | 9.0x10^-11 (8.0x10^-11, 1.0x10^-8) |
| | Zhoukou | 1 (0, 5) | 0.0372 (0.0331, 0.0421) | 0.8127 (0.7218, 0.9226) | 3.9x10^-11 (3.5x10^-11, 7.2x10^-9) |
| | Zhumadian | 2 (0, 5) | 0.0366 (0.0327, 0.0409) | 0.8026 (0.7156, 0.8983) | 2.2x10^-11 (2.0x10^-11, 6.8x10^-9) |
| Hubei | Wuhan | 7 (2, 13) | 0.048 (0.0427, 0.0537) | 0.8106 (0.7217, 0.9072) | 4.5x10^-11 (1.6x10^-11, |

| | | | | |
|---|---|---|---|---|
| | | | | 7.8x10^-9) |
| Huangshi | 1 (0, 6) | 0.0514 (0.0456, 0.0576) | 0.8523 (0.7557, 0.9568) | 8.1x10^-11 (7.2x10^-11, 3.1x10^-8) |
| Shiyan | 1 (0, 4) | 0.0318 (0.0279, 0.0355) | 0.541 (0.475, 0.6057) | 6.4x10^-13 (5.6x10^-13, 1.6x10^-8) |
| Yichang | 6 (1, 12) | 0.0441 (0.0394, 0.0496) | 0.7295 (0.6514, 0.8221) | 1.2x10^-12 (1.1x10^-12, 1.6x10^-8) |
| Xiangyang | 6 (2, 10) | 0.0388 (0.0345, 0.0439) | 0.647 (0.5742, 0.7328) | 4.7x10^-13 (4.2x10^-13, 9.9x10^-9) |
| Ezhou | 1 (0, 3) | 0.0517 (0.0457, 0.0582) | 0.7312 (0.6447, 0.8245) | 3.7x10^-13 (3.2x10^-13, 3.0x10^-8) |
| Jingmen | 4 (1, 9) | 0.0463 (0.0413, 0.0524) | 0.7517 (0.6691, 0.8521) | 1.2x10^-12 (1.1x10^-12, 2.4x10^-8) |
| Xiaogan | 2 (0, 5) | 0.0471 (0.0414, 0.0524) | 0.7393 (0.6491, 0.8236) | 4.2x10^-13 (3.7x10^-13, 1.4x10^-8) |
| Jingzhou | 5 (2, 11) | 0.0481 (0.0427, 0.0538) | 0.8096 (0.7187, 0.9075) | 5.1x10^-11 (4.5x10^-11, 1.2x10^-8) |
| Huanggang | 7 (3, 13) | 0.049 (0.0446, 0.0541) | 0.7961 (0.7237, 0.8789) | 9.6x10^-10 (8.1x10^-10, |

| | | | | | |
|---|---|---|---|---|---|
| | | | | | 1.1x10^-8) |
| | Xianning | 2 (0, 7) | 0.0484 (0.0429, 0.0541) | 0.7813 (0.6903, 0.8745) | 6.9x10^-13 (6.1x10^-13, 2.1x10^-8) |
| | Suizhou | 1 (0, 6) | 0.0412 (0.0365, 0.0461) | 0.6733 (0.5945, 0.7549) | 4.6x10^-13 (4.0x10^-13, 2.1x10^-8) |
| | Enshi Tujia and Miao Autonomous Prefecture | 11 (6, 19) | 0.0337 (0.0302, 0.0381) | 0.5516 (0.4921, 0.6228) | 1.0x10^-10 (2.5x10^-11, 1.3x10^-8) |
| Hunan | Changsha | 246 (191, 358) | 0.0526 (0.0474, 0.0596) | 0.2613 (0.2346, 0.2973) | 3.1x10^-7 (1.7x10^-7, 5.7x10^-7) |
| | Zhuzhou | 9 (5, 16) | 0.0525 (0.0463, 0.059) | 0.2534 (0.2228, 0.2859) | 2.5x10^-11 (5.1x10^-12, 8.9x10^-9) |
| | Xiangtan | 8 (3, 14) | 0.0506 (0.0442, 0.0576) | 0.2302 (0.2002, 0.263) | 3.3x10^-13 (2.9x10^-13, 6.8x10^-9) |
| | Hengyang | 187 (156, 265) | 0.0529 (0.0485, 0.0592) | 0.2697 (0.246, 0.3034) | 2.7x10^-7 (1.8x10^-7, 5.4x10^-7) |
| | Shaoyang | 206 (168, 255) | 0.0462 (0.0419, 0.0533) | 0.2408 (0.2173, 0.2791) | 3.3x10^-7 (2.0x10^-7, 5.6x10^-7) |
| | Yueyang | 8 (3, 13) | 0.0502 (0.0449, 0.0558) | 0.2499 (0.2222, 0.2784) | 1.5x10^-13 (1.3x10^-13, |

| | | | | | |
|---|---|---|---|---|---|
| | | | | | 3.7x10^-9) |
| | Changde | 10 (5, 17) | 0.0496 (0.0433, 0.0557) | 0.2546 (0.2217, 0.2871) | 3.6x10^-13 (3.1x10^-13, 3.7x10^-9) |
| | Zhangjiajie | 2 (0, 5) | 0.0457 (0.0406, 0.0511) | 0.2364 (0.2094, 0.2659) | 4.9x10^-13 (4.3x10^-13, 1.4x10^-8) |
| | Yiyang | 4 (1, 8) | 0.0475 (0.0422, 0.0533) | 0.2407 (0.2129, 0.271) | 3.5x10^-13 (3.1x10^-13, 6.8x10^-9) |
| | Chenzhou | 8 (3, 14) | 0.0483 (0.0444, 0.0545) | 0.2478 (0.227, 0.2811) | 9.4x10^-11 (6.3x10^-11, 6.9x10^-9) |
| | Yongzhou | 20 (15, 28) | 0.0544 (0.0492, 0.0612) | 0.2802 (0.2527, 0.3169) | 2.1x10^-10 (9.2x10^-11, 4.3x10^-9) |
| | Huaihua | 12 (6, 18) | 0.0432 (0.0394, 0.0486) | 0.2239 (0.2035, 0.2531) | 3.4x10^-11 (8.6x10^-12, 3.3x10^-9) |
| | Loudi | 7 (3, 12) | 0.0469 (0.043, 0.0529) | 0.241 (0.2199, 0.2736) | 1.0x10^-10 (7.9x10^-11, 4.2x10^-9) |
| | Xiangxi Tujia and Miao Autonomous Prefecture | 10 (5, 17) | 0.0437 (0.0387, 0.0495) | 0.219 (0.1927, 0.2486) | 5.6x10^-13 (4.9x10^-13, 7.9x10^-9) |
| Guangdong | Guangzhou | 4755 (3901, 5918) | 0.0805 (0.0742, 0.0869) | 1.5803 (1.4568, 1.7082) | 2.3x10^-6 (1.7x10^-6, |

| | | | | |
|---|---|---|---|---|
| | | | | 3.2x10^-6) |
| Shaoguan | 15 (8, 24) | 0.0593 (0.0528, 0.0669) | 1.2685 (1.1266, 1.4321) | 5.7x10^-10 (1.8x10^-12, 5.5x10^-8) |
| Shenzhen | 878 (696, 1180) | 0.0735 (0.0659, 0.0822) | 1.5146 (1.3564, 1.6958) | 5.5x10^-7 (3.5x10^-7, 8.7x10^-7) |
| Zhuhai | 81 (63, 140) | 0.0777 (0.0698, 0.0881) | 1.5572 (1.3968, 1.7688) | 1.2x10^-7 (3.2x10^-8, 4.5x10^-7) |
| Shantou | 3325 (2716, 4365) | 0.0638 (0.0598, 0.0715) | 1.4805 (1.3851, 1.6595) | 3.4x10^-6 (2.0x10^-6, 6.0x10^-6) |
| Foshan | 1232 (1037, 1494) | 0.0671 (0.0623, 0.0764) | 1.2326 (1.1428, 1.404) | 7.0x10^-7 (4.7x10^-7, 1.0x10^-6) |
| Jiangmen | 852 (670, 1053) | 0.078 (0.0707, 0.0864) | 1.5966 (1.4454, 1.7695) | 1.6x10^-6 (9.7x10^-7, 2.5x10^-6) |
| Zhanjiang | 1822 (1460, 2313) | 0.0705 (0.0648, 0.0777) | 1.6304 (1.497, 1.7995) | 3.4x10^-6 (2.4x10^-6, 5.0x10^-6) |
| Maoming | 98 (73, 160) | 0.0841 (0.0758, 0.0927) | 1.9194 (1.727, 2.1159) | 9.3x10^-8 (2.9x10^-8, 2.6x10^-7) |
| Zhaoqing | 60 (42, 96) | 0.0625 (0.0572, 0.0725) | 1.2958 (1.184, 1.5066) | 2.5x10^-8 (5.1x10^-9, |

| | | | | |
|---|---|---|---|---|
| | | | | 1.3x10^-7) |
| Huizhou | 215 (162, 274) | 0.0661 (0.0591, 0.0739) | 1.1991 (1.0706, 1.3426) | 1.1x10^-7 (4.8x10^-8, 2.9x10^-7) |
| Meizhou | 17 (10, 28) | 0.0633 (0.0566, 0.0717) | 1.4251 (1.2721, 1.617) | 3.0x10^-9 (1.8x10^-10, 6.5x10^-8) |
| Shanwei | 18 (10, 27) | 0.0669 (0.0593, 0.0743) | 1.4714 (1.3027, 1.636) | 1.0x10^-9 (1.6x10^-12, 6.9x10^-8) |
| Heyuan | 17 (8, 26) | 0.0604 (0.0553, 0.0702) | 1.2664 (1.1574, 1.4762) | 4.9x10^-10 (1.0x10^-10, 5.3x10^-8) |
| Yangjiang | 26 (13, 38) | 0.0724 (0.0656, 0.0824) | 1.5704 (1.4219, 1.7892) | 8.0x10^-9 (2.1x10^-10, 1.0x10^-7) |
| Qingyuan | 232 (192, 292) | 0.0669 (0.0604, 0.074) | 1.361 (1.228, 1.5082) | 3.2x10^-7 (1.7x10^-7, 5.6x10^-7) |
| Dongguan | 488 (377, 579) | 0.0683 (0.0621, 0.0773) | 1.1563 (1.0512, 1.3107) | 1.8x10^-7 (9.3x10^-8, 3.3x10^-7) |
| Zhongshan | 778 (605, 1006) | 0.0791 (0.0717, 0.0886) | 1.5136 (1.3704, 1.6978) | 1.4x10^-6 (8.8x10^-7, 2.1x10^-6) |
| Chaozhou | 1212 (994, 1466) | 0.0642 (0.0591, 0.071) | 1.4248 (1.3096, 1.579) | 4.9x10^-6 (3.3x10^-6, |

| | | | | | |
|---|---|---|---|---|---|
| | | | | | 7.2x10^-6) |
| | Jieyang | 964 (777, 1248) | 0.0644 (0.0595, 0.0729) | 1.4447 (1.3339, 1.6378) | 1.5x10^-6 (9.2x10^-7, 2.6x10^-6) |
| | Yunfu | 68 (49, 103) | 0.0762 (0.0677, 0.0844) | 1.5929 (1.4126, 1.7667) | 1.2x10^-7 (4.0x10^-8, 4.1x10^-7) |
| Guangxi | Nanning | 3852 (3155, 4974) | 0.0665 (0.0616, 0.0731) | 2.2138 (2.0504, 2.4342) | 6.8x10^-6 (4.8x10^-6, 9.6x10^-6) |
| | Liuzhou | 32 (16, 77) | 0.0582 (0.052, 0.0646) | 1.9029 (1.6974, 2.1172) | 2.8x10^-8 (1.1x10^-9, 2.3x10^-7) |
| | Guilin | 26 (15, 49) | 0.061 (0.0542, 0.0684) | 1.9964 (1.7686, 2.2403) | 1.1x10^-8 (4.3x10^-10, 1.1x10^-7) |
| | Wuzhou | 309 (219, 413) | 0.0619 (0.0555, 0.0695) | 2.0037 (1.7931, 2.2529) | 1.0x10^-6 (5.7x10^-7, 1.7x10^-6) |
| | Beihai | 145 (98, 224) | 0.0686 (0.0615, 0.0781) | 2.2601 (2.023, 2.582) | 1.5x10^-7 (6.5x10^-8, 6.7x10^-7) |
| | Fangchenggang | 471 (344, 671) | 0.0626 (0.0582, 0.0698) | 1.998 (1.8548, 2.235) | 6.6x10^-6 (3.4x10^-6, 1.2x10^-5) |
| | Qinzhou | 72 (43, 144) | 0.0642 (0.0576, 0.0717) | 2.0925 (1.8726, 2.3398) | 5.6x10^-8 (4.5x10^-9, |

| | | | | | |
|---|---|---|---|---|---|
| | | | | | 4.8x10^-7) |
| | Guigang | 630 (483, 834) | 0.0639 (0.0584, 0.0709) | 2.1291 (1.9405, 2.3622) | 2.2x10^-6 (1.4x10^-6, 3.6x10^-6) |
| | Haerbin | 626 (471, 851) | 0.0664 (0.0599, 0.0751) | 2.2356 (2.0165, 2.5326) | 1.5x10^-6 (9.3x10^-7, 2.6x10^-6) |
| | Baise | 42 (24, 82) | 0.0509 (0.046, 0.0572) | 1.6587 (1.4964, 1.8639) | 1.7x10^-8 (2.0x10^-10, 2.1x10^-7) |
| | Hezhou | 9 (4, 23) | 0.0623 (0.0552, 0.0697) | 2.0294 (1.792, 2.2755) | 3.1x10^-9 (5.9x10^-10, 1.5x10^-7) |
| | Hechi | 22 (8, 51) | 0.054 (0.0479, 0.0602) | 1.7714 (1.5692, 1.9797) | 9.7x10^-9 (1.6x10^-12, 1.2x10^-7) |
| | Laibin | 27 (16, 45) | 0.056 (0.05, 0.0626) | 1.7163 (1.5282, 1.926) | 1.1x10^-8 (1.3x10^-10, 1.6x10^-7) |
| | Chongzuo | 62 (40, 106) | 0.0581 (0.0526, 0.066) | 1.8751 (1.6947, 2.1354) | 1.6x10^-7 (2.7x10^-8, 5.4x10^-7) |
| Hainan | Haikou | 695 (546, 888) | 0.0824 (0.0762, 0.0912) | 1.4122 (1.302, 1.5659) | 3.6x10^-6 (2.2x10^-6, 5.5x10^-6) |
| | Sanya | 5 (1, 14) | 0.0597 (0.0531, 0.0667) | 0.9834 (0.8699, 1.102) | 4.3x10^-12 (3.8x10^-12, |

| | | | | | |
|---|---|---|---|---|---|
| | | | | | 9.3x10^-8) |
| | Sansha | 0 (0, 0) | 0.0707 (0.0628, 0.0786) | 1.2444 (0.9765, 1.5277) | 0 (0, 0) |
| | Danzhou | 170 (116, 232) | 0.0708 (0.0639, 0.0799) | 1.1751 (1.055, 1.3339) | 2.2x10^-6 (1.1x10^-6, 4.0x10^-6) |
| Chongqing | Chongqing | 4151 (3434, 4871) | 0.0494 (0.0444, 0.0529) | 1.3322 (1.1976, 1.4272) | 2.0x10^-6 (1.4x10^-6, 2.6x10^-6) |
| Sichuan | Chengdu | 28 (20, 42) | 0.0337 (0.0294, 0.038) | 0.3385 (0.2952, 0.3823) | 2.0x10^-11 (1.3x10^-13, 2.5x10^-9) |
| | Zigong | 7 (2, 11) | 0.04 (0.0365, 0.046) | 0.3862 (0.3513, 0.4449) | 5.0x10^-13 (4.5x10^-13, 1.0x10^-8) |
| | Panzhihua | 3 (0, 9) | 0.0325 (0.029, 0.0366) | 0.3258 (0.2898, 0.3683) | 8.7x10^-13 (7.7x10^-13, 2.0x10^-8) |
| | Luzhou | 31 (24, 40) | 0.0416 (0.0373, 0.0464) | 0.4084 (0.3653, 0.4564) | 3.5x10^-10 (1.1x10^-10, 1.0x10^-8) |
| | Deyang | 2 (0, 5) | 0.0333 (0.0294, 0.0372) | 0.2816 (0.2487, 0.3159) | 2.0x10^-11 (1.9x10^-11, 4.4x10^-9) |
| | Mianyang | 3 (0, 6) | 0.0315 (0.028, 0.0357) | 0.309 (0.2737, 0.3503) | 4.5x10^-11 (3.9x10^-11, 5.2x10^-9) |

| | | | | |
|---|---|---|---|---|
| Guangyuan | 5 (1, 8) | 0.0262 (0.0237, 0.0296) | 0.2612 (0.2358, 0.2965) | 3.2x10^-11 (9.2x10^-12, 1.0x10^-8) |
| Suining | 9 (3, 15) | 0.0347 (0.0308, 0.0395) | 0.3315 (0.2938, 0.3778) | 6.7x10^-13 (5.9x10^-13, 7.6x10^-9) |
| Neijiang | 8 (3, 14) | 0.0402 (0.0355, 0.0453) | 0.3862 (0.3405, 0.4358) | 5.7x10^-13 (5.0x10^-13, 9.4x10^-9) |
| Leshan | 3 (0, 8) | 0.0287 (0.0254, 0.0324) | 0.2845 (0.2514, 0.3216) | 4.2x10^-13 (3.7x10^-13, 7.9x10^-9) |
| Nanchong | 11 (6, 19) | 0.0344 (0.0308, 0.0389) | 0.3491 (0.3124, 0.3963) | 3.9x10^-13 (3.5x10^-13, 5.1x10^-9) |
| Meishan | 3 (0, 9) | 0.0367 (0.0326, 0.041) | 0.3115 (0.2763, 0.3492) | 4.5x10^-13 (4.0x10^-13, 6.3x10^-9) |
| Yibin | 11 (5, 18) | 0.0396 (0.0355, 0.0451) | 0.4025 (0.3595, 0.4592) | 2.9x10^-11 (2.6x10^-11, 7.9x10^-9) |
| Guangan | 25 (15, 34) | 0.0381 (0.0342, 0.0431) | 0.3687 (0.3308, 0.419) | 7.9x10^-11 (1.5x10^-11, 1.2x10^-8) |
| Dazhou | 14 (7, 21) | 0.0349 (0.0312, 0.0399) | 0.3623 (0.3236, 0.4148) | 2.1x10^-11 (5.7x10^-12, 6.9x10^-9) |

| | | | | | |
|---|---|---|---|---|---|
| | Yaan | 3 (0, 8) | 0.0285 (0.0251, 0.032) | 0.2532 (0.2232, 0.2852) | 5.0x10^-13 (4.4x10^-13, 1.1x10^-8) |
| | Bazhong | 1 (0, 5) | 0.0299 (0.0264, 0.0337) | 0.3153 (0.2788, 0.3562) | 2.8x10^-13 (2.4x10^-13, 6.9x10^-9) |
| | Ziyang | 10 (5, 18) | 0.0353 (0.0314, 0.0395) | 0.3212 (0.2857, 0.3605) | 1.4x10^-11 (3.7x10^-12, 9.7x10^-9) |
| | Aba (Ngawa) Tibetan and Qiang Autonomous Prefecture | 1 (0, 3) | 0.0045 (0.004, 0.0051) | 0.046 (0.0406, 0.0521) | 6.2x10^-15 (5.5x10^-15, 7.9x10^-11) |
| | Ganzi (Garzê) Tibetan Autonomous Prefecture | 1 (0, 3) | 0.0041 (0.0036, 0.0046) | 0.0454 (0.0399, 0.0512) | 7.7x10^-15 (6.8x10^-15, 7.7x10^-10) |
| | Liangshan Yi Autonomous Prefecture | 4 (1, 9) | 0.0238 (0.021, 0.0269) | 0.2511 (0.221, 0.2839) | 1.3x10^-13 (1.2x10^-13, 2.8x10^-9) |
| Guizhou | Guiyang | 16 (7, 26) | 0.0279 (0.025, 0.0316) | 0.2253 (0.2014, 0.2564) | 3.3x10^-13 (3.0x10^-13, 3.7x10^-9) |
| | Liupanshui | 10 (4, 20) | 0.0218 (0.0196, 0.0247) | 0.1848 (0.1654, 0.2093) | 5.8x10^-13 (5.2x10^-13, 5.3x10^-9) |
| | Zunyi | 20 (12, 29) | 0.0345 (0.0306, 0.0387) | 0.2837 (0.2517, 0.3192) | 4.7x10^-13 (4.2x10^-13, 4.4x10^-9) |

| | | | | | |
|---|---|---|---|---|---|
| | Anshun | 10 (4, 18) | 0.0295 (0.0261, 0.0329) | 0.2278 (0.2004, 0.2549) | 5.5x10^-13 (4.9x10^-13, 7.5x10^-9) |
| | Bijie | 9 (4, 16) | 0.0225 (0.0199, 0.0254) | 0.1918 (0.1697, 0.2171) | 8.4x10^-12 (2.4x10^-12, 2.0x10^-9) |
| | Tongren | 12 (6, 19) | 0.0409 (0.0372, 0.0465) | 0.3416 (0.3092, 0.3892) | 9.1x10^-11 (8.2x10^-11, 8.5x10^-9) |
| | Qianxinan Buyi and Miao Autonomous Prefecture | 8 (3, 15) | 0.0322 (0.0284, 0.0363) | 0.272 (0.2391, 0.3083) | 4.7x10^-13 (4.1x10^-13, 6.5x10^-9) |
| | Qiandongnan Miao and Dong Autonomous Prefecture | 7 (2, 12) | 0.0396 (0.0349, 0.0448) | 0.3357 (0.2952, 0.3811) | 4.6x10^-13 (4.1x10^-13, 7.1x10^-9) |
| | Qiannan Buyi and Miao Autonomous Prefecture | 13 (6, 23) | 0.038 (0.0333, 0.043) | 0.2901 (0.2537, 0.3298) | 2.0x10^-11 (4.7x10^-12, 7.8x10^-9) |
| Yunnan | Kunming | 362 (283, 475) | 0.0247 (0.0227, 0.0279) | 1.3641 (1.2603, 1.5348) | 2.2x10^-7 (9.8x10^-8, 4.9x10^-7) |
| | Qujing | 49 (31, 75) | 0.0196 (0.0177, 0.0225) | 1.0615 (0.9532, 1.2123) | 1.2x10^-8 (5.5x10^-10, 7.1x10^-8) |
| | Yuxi | 197 (127, 317) | 0.033 (0.0305, 0.0367) | 1.678 (1.5532, 1.8632) | 2.5x10^-7 (2.2x10^-8, 1.0x10^-6) |

| | | | | |
|---|---|---|---|---|
| Baoshan | 163 (101, 314) | 0.0313 (0.0285, 0.0348) | 1.7942 (1.6355, 1.988) | 1.5x10^-7 (2.3x10^-9, 1.2x10^-6) |
| Zhaotong | 25 (15, 46) | 0.029 (0.0255, 0.0328) | 1.6097 (1.4195, 1.8263) | 1.4x10^-8 (2.6x10^-9, 9.5x10^-8) |
| Lijiang | 10 (4, 17) | 0.0211 (0.0186, 0.0239) | 1.1574 (1.0215, 1.3146) | 4.3x10^-9 (3.2x10^-11, 8.0x10^-8) |
| Pu’er | 901 (661, 1203) | 0.0401 (0.0377, 0.0453) | 2.3125 (2.1769, 2.6038) | 3.3x10^-6 (1.6x10^-6, 6.7x10^-6) |
| Lincang | 1002 (763, 1399) | 0.0415 (0.0381, 0.046) | 2.4552 (2.2579, 2.7164) | 7.0x10^-6 (3.8x10^-6, 1.2x10^-5) |
| Chuxiong Yi Autonomous Prefecture | 35 (22, 75) | 0.0306 (0.0275, 0.0349) | 1.6337 (1.4687, 1.864) | 1.5x10^-8 (4.5x10^-11, 2.2x10^-7) |
| Honghe Hani and Yi Autonomous Prefecture | 611 (472, 850) | 0.0421 (0.0387, 0.0482) | 2.4061 (2.2163, 2.7503) | 1.7x10^-6 (9.9x10^-7, 3.2x10^-6) |
| Wenshan Zhuang and Miao Autonomous Prefecture | 34 (19, 63) | 0.0348 (0.0316, 0.0395) | 2.0064 (1.8242, 2.274) | 1.8x10^-8 (2.7x10^-11, 2.2x10^-7) |
| Dai Autonomous Prefecture of Xishuangbanna/Sipsongpanna | 13753 (11240, 17205) | 0.0502 (0.0469, 0.0558) | 2.9377 (2.7562, 3.2702) | 1.5x10^-4 (1.0x10^-4, 2.2x10^-4) |

| | | | | | |
|---|---|---|---|---|---|
| | Dali Bai Autonomous Prefecture | 90 (63, 161) | 0.0285 (0.0256, 0.0324) | 1.5616 (1.4029, 1.778) | 6.8x10^-8 (3.9x10^-9, 3.6x10^-7) |
| | Dehong Dai and Jingpo Autonomous Prefecture | 4860 (3882, 6264) | 0.0479 (0.0448, 0.0533) | 2.8391 (2.6613, 3.1603) | 5.8x10^-5 (3.9x10^-5, 8.9x10^-5) |
| | Nujiang Lisu Autonomous Prefecture | 2 (0, 10) | 0.0299 (0.0265, 0.0335) | 1.7044 (1.5061, 1.9158) | 4.1x10^-12 (3.6x10^-12, 3.0x10^-7) |
| | Diqing Tibetan Autonomous Prefecture | 1 (0, 5) | 0.0024 (0.0021, 0.0027) | 0.1142 (0.1001, 0.1303) | 3.7x10^-13 (3.3x10^-13, 7.4x10^-9) |
| Tibet | Lhasa | 0 (0, 1) | 0.0024 (0.0021, 0.0027) | 0.0078 (0.0068, 0.009) | 0 (0, 0) |
| | Rikaze | 0 (0, 1) | 0.0007 (0.0006, 0.0008) | 0.0023 (0.002, 0.0026) | 0 (0, 0) |
| | Changdu | 0 (0, 1) | 0.002 (0.0018, 0.0023) | 0.0068 (0.0059, 0.0078) | 0 (0, 0) |
| | Linzhi | 0 (0, 1) | 0.008 (0.007, 0.0089) | 0.0237 (0.0203, 0.0272) | 1.5x10^-14 (1.3x10^-14, 1.6x10^-14) |
| | Shannan | 0 (0, 1) | 0.0014 (0.0012, 0.0015) | 0.0043 (0.0037, 0.0049) | 0 (0, 0) |
| | Naqu | 0 (0, 1) | 0.0006 (0.0006, 0.0007) | 0.002 (0.0018, 0.0023) | 0 (0, 0) |
| | Ngari Prefecture | 0 (0, 0) | 0.0017 (0.0015, 0.0019) | 0.0057 (0.0049, 0.0066) | 0 (0, 0) |

| | | | | | |
|---|---|---|---|---|---|
| Shaanxi | Xi’an | 5 (1, 10) | 0.0217 (0.0191, 0.0243) | 0.0115 (0.01, 0.0129) | 1.6x10^-14 (1.4x10^-14, 7.9x10^-12) |
| | Tongchuan | 0 (0, 1) | 0.0127 (0.0112, 0.0143) | 0.0065 (0.0055, 0.0075) | 0 (0, 0) |
| | Baoji | 0 (0, 2) | 0.0147 (0.013, 0.0166) | 0.0081 (0.0071, 0.0093) | 0 (0, 0) |
| | Xianyang | 1 (0, 5) | 0.0165 (0.0146, 0.0186) | 0.0077 (0.0068, 0.0088) | 0 (0, 0) |
| | Weinan | 2 (0, 6) | 0.0189 (0.0167, 0.0212) | 0.0098 (0.0086, 0.0112) | 0 (0, 0) |
| | Yan’an | 0 (0, 2) | 0.0096 (0.0084, 0.0108) | 0.0053 (0.0046, 0.0061) | 0 (0, 0) |
| | Hanzhong | 2 (0, 6) | 0.0186 (0.0165, 0.0208) | 0.0099 (0.0086, 0.0112) | 0 (0, 0) |
| | Yulin | 1 (0, 3) | 0.0132 (0.0117, 0.0148) | 0.007 (0.0061, 0.008) | 0 (0, 0) |
| | Ankang | 3 (0, 7) | 0.0225 (0.0199, 0.0252) | 0.0116 (0.0101, 0.0132) | 0 (0, 0) |
| | Shangluo | 1 (0, 4) | 0.0164 (0.0144, 0.0184) | 0.0086 (0.0074, 0.0099) | 0 (0, 0) |
| Gansu | Lanzhou | 4 (0, 8) | 0.0059 (0.0052, 0.0068) | 0.0196 (0.0172, 0.0225) | 4.8x10^-15 (4.2x10^-15, 1.9x10^-12) |
| | Jiayuguan | 0 (0, 1) | 0.0096 (0.0085, 0.0109) | 0.0295 (0.0256, 0.034) | 9.3x10^-15 (8.3x10^-15, 1.0x10^-14) |

| | | | | |
|---|---|---|---|---|
| Jinchang | 0 (0, 2) | 0.0034 (0.003, 0.0038) | 0.0109 (0.0095, 0.0126) | 1.4x10^-14 (1.2x10^-14, 3.1x10^-12) |
| Baiyin | 0 (0, 3) | 0.0093 (0.0082, 0.0104) | 0.0293 (0.0256, 0.033) | 3.5x10^-14 (3.0x10^-14, 2.1x10^-11) |
| Tianshui | 3 (1, 8) | 0.0102 (0.009, 0.0114) | 0.0341 (0.03, 0.0387) | 1.9x10^-15 (1.7x10^-15, 8.8x10^-13) |
| Wuwei | 2 (0, 6) | 0.0032 (0.0028, 0.0036) | 0.0102 (0.009, 0.0116) | 0 (0, 0) |
| Zhangye | 1 (0, 6) | 0.0074 (0.0065, 0.0083) | 0.0236 (0.0206, 0.0268) | 1.5x10^-14 (1.3x10^-14, 1.9x10^-9) |
| Pingliang | 0 (0, 2) | 0.0069 (0.0061, 0.0078) | 0.0225 (0.0198, 0.0256) | 8.6x10^-15 (7.6x10^-15, 7.6x10^-12) |
| Jiuquan | 3 (0, 7) | 0.0122 (0.0108, 0.0137) | 0.0371 (0.0324, 0.0421) | 7.0x10^-14 (6.2x10^-14, 2.8x10^-11) |
| Qingyang | 0 (0, 1) | 0.0079 (0.007, 0.0089) | 0.0272 (0.0239, 0.031) | 0 (0, 0) |
| Dingxi | 3 (0, 8) | 0.0028 (0.0025, 0.0031) | 0.0092 (0.0081, 0.0104) | 3.7x10^-15 (3.3x10^-15, 3.8x10^-12) |
| Longnan | 4 (1, 9) | 0.013 (0.0116, 0.0147) | 0.0439 (0.0387, 0.05) | 1.3x10^-13 (1.2x10^-13, |

| | | | | | |
|---|---|---|---|---|---|
| | | | | | 9.0x10^-11) |
| | Linxia Hui Autonomous Prefecture | 0 (0, 1) | 0.0033 (0.003, 0.0037) | 0.0113 (0.01, 0.0127) | 0 (0, 0) |
| | Gannan Tibetan Autonomous Prefecture | 0 (0, 1) | 0.0015 (0.0014, 0.0017) | 0.0051 (0.0045, 0.0058) | 0 (0, 0) |
| Qinghai | Xining | 0 (0, 2) | 0.0014 (0.0012, 0.0016) | 0.0046 (0.0041, 0.0053) | 0 (0, 0) |
| | Haidong | 0 (0, 2) | 0.0033 (0.0029, 0.0037) | 0.01 (0.0088, 0.0114) | 0 (0, 0) |
| | Haibei Tibetan Autonomous Prefecture | 0 (0, 1) | 0.0006 (0.0006, 0.0007) | 0.002 (0.0017, 0.0023) | 0 (0, 0) |
| | Huangnan Tibetan Autonomous Prefecture | 0 (0, 0) | 0.0009 (0.0008, 0.001) | 0.0031 (0.0027, 0.0035) | 0 (0, 0) |
| | Hainan Tibetan Autonomous Prefecture | 0 (0, 1) | 0.0014 (0.0012, 0.0015) | 0.004 (0.0035, 0.0046) | 0 (0, 0) |
| | Guoluo (Golog) Tibetan Autonomous Prefecture | 0 (0, 1) | 0.0006 (0.0006, 0.0007) | 0.002 (0.0017, 0.0023) | 0 (0, 0) |
| | Yushu Tibetan Autonomous Prefecture | 0 (0, 0) | 0.0006 (0.0006, 0.0007) | 0.0021 (0.0018, 0.0024) | 0 (0, 0) |
| | Haixi Mongolian and Tibetan Autonomous Prefecture | 0 (0, 2) | 0.0022 (0.0019, 0.0024) | 0.0063 (0.0055, 0.0071) | 0 (0, 0) |
| Ningxia | Yinchuan | 0 (0, 2) | 0.0081 (0.0071, 0.0091) | 0.0262 (0.0229, 0.0297) | 1.1x10^-16 (9.9x10^-17, 1.2x10^-16) |
| | Shizuishan | 0 (0, 1) | 0.0138 (0.0122, 0.0153) | 0.0393 (0.0344, 0.0442) | 0 (0, 0) |
| | Wuzhong | 0 (0, 1) | 0.0114 (0.01, | 0.033 (0.0286, | 1.9x10^-15 |

| | | | | | |
|---|---|---|---|---|---|
| | | | 0.0127) | 0.0373) | (1.7x10^-15, 2.2x10^-15) |
| | Guyuan | 0 (0, 2) | 0.0016 (0.0014, 0.0018) | 0.0053 (0.0046, 0.006) | 0 (0, 0) |
| | Zhongwei | 0 (0, 2) | 0.0102 (0.009, 0.0114) | 0.0304 (0.0266, 0.0345) | 1.7x10^-14 (1.5x10^-14, 1.8x10^-11) |
| Xinjiang | Urumqi | 1 (0, 4) | 0.0057 (0.005, 0.0064) | 0.0187 (0.0164, 0.0211) | 2.4x10^-15 (2.1x10^-15, 2.5x10^-12) |
| | Karamay | 0 (0, 1) | 0.0228 (0.0201, 0.0255) | 0.0648 (0.0561, 0.0737) | 2.2x10^-14 (1.9x10^-14, 1.0x10^-11) |
| | Tulufan | 1 (0, 3) | 0.0377 (0.0333, 0.0425) | 0.1077 (0.0938, 0.123) | 1.9x10^-13 (1.7x10^-13, 1.1x10^-8) |
| | Hami | 1 (0, 5) | 0.0187 (0.0166, 0.0211) | 0.0563 (0.0494, 0.0646) | 1.2x10^-13 (1.1x10^-13, 6.7x10^-11) |
| | Changji Hui Autonomous Prefecture | 0 (0, 2) | 0.0117 (0.0104, 0.0133) | 0.0316 (0.0277, 0.0361) | 1.0x10^-14 (9.0x10^-15, 7.0x10^-12) |
| | Bortala Mongolian Autonomous Prefecture | 0 (0, 3) | 0.0123 (0.011, 0.0139) | 0.0382 (0.0333, 0.0438) | 0 (0, 0) |
| | Bayingol Mongolian Autonomous Prefecture | 0 (0, 1) | 0.0149 (0.0132, 0.0168) | 0.0469 (0.041, 0.0534) | 1.2x10^-14 (1.1x10^-14, 1.9x10^-12) |

| | | | | |
|---|---|---|---|---|
| Aksu Prefecture | 0 (0, 1) | 0.0163 (0.0145, 0.0181) | 0.0543 (0.0479, 0.0608) | 0 (0, 0) |
| Kizilsu Kirgiz Autonomous Prefecture | 0 (0, 0) | 0.0049 (0.0043, 0.0054) | 0.0159 (0.0139, 0.0179) | 0 (0, 0) |
| Kashgar Prefecture | 0 (0, 1) | 0.0202 (0.0178, 0.0226) | 0.0684 (0.0599, 0.0769) | 8.8x10^-16 (7.8x10^-16, 9.9x10^-16) |
| Hotan Prefecture | 0 (0, 0) | 0.0222 (0.0196, 0.025) | 0.0751 (0.0659, 0.0853) | 1.7x10^-15 (1.5x10^-15, 1.9x10^-15) |
| Ili Kazakh Autonomous Prefecture | 0 (0, 3) | 0.0116 (0.0101, 0.0129) | 0.0386 (0.0334, 0.0434) | 1.2x10^-14 (1.1x10^-14, 2.1x10^-9) |
| Tarbagatay Prefecture | 0 (0, 3) | 0.0111 (0.0098, 0.0125) | 0.0342 (0.0299, 0.0388) | 6.1x10^-15 (5.3x10^-15, 8.4x10^-10) |
| Altay Prefecture | 0 (0, 1) | 0.0081 (0.0072, 0.0091) | 0.0266 (0.0232, 0.0305) | 0 (0, 0) |

**Reference:**


1 Ministry of Health of the People's Republic of China. Diagnostic Criteria for Dengue Fever (WS 216-2008). 2008.

2 WHO. Dengue guidelines, for diagnosis, treatment, prevention and control, 2009. https://www.who.int/publications/i/item/9789241547871

3 National health commission of the People's Republic of China. Diagnosis for dengue fever (WS 216-2018). 2018.

4 Naumova EN, Jagai JS, Matyas B, DeMARIA A, MacNEILL IB, Griffiths JK. Seasonality in six enterically transmitted diseases and ambient temperature. *Epidemiol Infect* 2007; 135: 281–92.

5 Yu H, Alonso WJ, Feng L, *et al.* Characterization of Regional Influenza Seasonality Patterns in China and Implications for Vaccination Strategies: Spatio-Temporal Modeling of Surveillance Data. *PLoS Med* 2013; 10: e1001552.

6 Xing W, Liao Q, Viboud C, *et al.* Hand, foot, and mouth disease in China, 2008–12: an epidemiological study. *The Lancet Infectious Diseases* 2014; 14: 308–18.

7 Zeng Q, Yu X, Ni H, *et al.* Dengue transmission dynamics prediction by combining metapopulation networks and Kalman filter algorithm. *PLoS Negl Trop Dis* 2023; 17: e0011418.

8 Pei S, Yamana TK, Kandula S, Galanti M, Shaman J. Burden and characteristics of COVID-19 in the United States during 2020. *Nature* 2021; 598: 338–41.

9 Scott TW, Amerasinghe PH, Morrison AC, *et al.* Longitudinal Studies of *Aedes aegypti* (Diptera: Culicidae) in Thailand and Puerto Rico: Blood Feeding Frequency. *J Med Entomol* 2000; 37: 89–101.

10 Lambrechts L, Paaijmans KP, Fansiri T, *et al.* Impact of daily temperature fluctuations on dengue virus transmission by *Aedes aegypti*. *Proc Natl Acad Sci USA* 2011; 108: 7460–5.

11 Chen Y, Liu T, Yu X, *et al.* An ensemble forecast system for tracking dynamics of dengue outbreaks and its validation in China. *PLoS Comput Biol* 2022; 18: e1010218.

12 Anderson JL. An Ensemble Adjustment Kalman Filter for Data Assimilation. *Mon Wea Rev* 2001; 129: 2884–903.

13 Shaman J, Karspeck A. Forecasting seasonal outbreaks of influenza. *Proc Natl Acad Sci USA* 2012; 109: 20425–30.

14 Pei S, Kandula S, Yang W, Shaman J. Forecasting the spatial transmission of influenza in

the United States. *Proc Natl Acad Sci USA* 2018; 115: 2752–7.
