# Supplementary material for: Epidemiological characteristics and transmission dynamics of dengue fever in China
Source: Nat Commun. 2024 Sep 14;15:8060. doi: 10.1038/s41467-024-52460-w (PMC11401889; doi:10.1038/s41467-024-52460-w)
Supplement: Supplementary file 3 — Description of Additional Supplementary Files [file 41467_2024_52460_MOESM3_ESM.pdf]

Supplementary Movie 1.
Daily population movement in cities across the nation in 2019.

Supplementary Movie 2.
Daily estimates of dengue infections (including reported and unreported cases) in cities across the nation in 2019.

Supplementary Movie 3.
Daily estimates of dengue transmission rate in cities across the nation in 2019.

Supplementary Movie 4.
Daily estimates of dengue effective reproduction number in cities across the nation in 2019.

Supplementary Movie 5.
Daily estimates of dengue force of infection in cities across the nation in 2019.

Supplementary Movie 6.
Daily estimates of dengue infections (including reported and unreported cases) due to population movements in cities across the nation in 2019.
