# Supplementary material for: Epidemiological characteristics and transmission dynamics of dengue fever in China
Source: Nat Commun. 2024 Sep 14;15:8060. doi: 10.1038/s41467-024-52460-w (PMC11401889; doi:10.1038/s41467-024-52460-w)
Supplement: Supplementary file 10 — Reporting Summary [file 41467_2024_52460_MOESM10_ESM.pdf]

nature portfolio

Corresponding author(s): Pi Guo

Last updated by author(s): Jul 4, 2024

Reporting Summary

Nature Portfolio wishes to improve the reproducibility of the work that we publish. This form provides structure for consistency and transparency in reporting. For further information on Nature Portfolio policies, see our Editorial Policies and the Editorial Policy Checklist.

Statistics

For all statistical analyses, confirm that the following items are present in the figure legend, table legend, main text, or Methods section.

n/a	Confirmed	
☐	☒	The exact sample size (*n*) for each experimental group/condition, given as a discrete number and unit of measurement
☐	☒	A statement on whether measurements were taken from distinct samples or whether the same sample was measured repeatedly
☐	☒	The statistical test(s) used AND whether they are one- or two-sided<br>*Only common tests should be described solely by name; describe more complex techniques in the Methods section.*
☐	☒	A description of all covariates tested
☐	☒	A description of any assumptions or corrections, such as tests of normality and adjustment for multiple comparisons
☐	☒	A full description of the statistical parameters including central tendency (e.g. means) or other basic estimates (e.g. regression coefficient) AND variation (e.g. standard deviation) or associated estimates of uncertainty (e.g. confidence intervals)
☐	☒	For null hypothesis testing, the test statistic (e.g. *F*, *t*, *r*) with confidence intervals, effect sizes, degrees of freedom and *P* value noted<br>*Give P values as exact values whenever suitable.*
☐	☒	For Bayesian analysis, information on the choice of priors and Markov chain Monte Carlo settings
☒	☐	For hierarchical and complex designs, identification of the appropriate level for tests and full reporting of outcomes
☒	☐	Estimates of effect sizes (e.g. Cohen's *d*, Pearson's *r*), indicating how they were calculated

Our web collection on statistics for biologists contains articles on many of the points above.

Software and code

Policy information about availability of computer code

Data collection	We collected the data into two major formats including Microsoft Excel and csv format.
Data analysis	Data are analyzed using R version 3.4.3 and MATLAB R2022b. The EAKF algorithm is implemented in MATLAB R2022b. Custom codes developed for this study are available at https://github.com/tinkzzz/DENGUE_CHINA.git.

For manuscripts utilizing custom algorithms or software that are central to the research but not yet described in published literature, software must be made available to editors and reviewers. We strongly encourage code deposition in a community repository (e.g. GitHub). See the Nature Portfolio guidelines for submitting code & software for further information.

Data

Policy information about availability of data

All manuscripts must include a data availability statement. This statement should provide the following information, where applicable:

- Accession codes, unique identifiers, or web links for publicly available datasets
- A description of any restrictions on data availability
- For clinical datasets or third party data, please ensure that the statement adheres to our policy

Dengue surveillance data are available at the National Notifiable Disease Surveillance System of the Chinese Center for Disease Control and Prevention (China CDC). Mosquito vector density data was obtained from the Chinese Centre for Disease Control and Prevention, which cannot be made publicly available. Researchers can refer to the corresponding authors for information on accessing the data. All other data utilized in this study were accessed from open sources. Meteorological data

was obtained from the China Meteorological Data Sharing System (http://data.cma.cn/). Demographic and mobility data was collected from the China's seventh census in 2020, which can be publicly obtained from the National Bureau of Statistics of China (http://www.stats.gov.cn/). The population migration data at daily city-level was obtained from the platform of Gaode Migration Big Data (https://trp.autonavi.com/migrate/page.do).

Research involving human participants, their data, or biological material

Policy information about studies with human participants or human data. See also policy information about sex, gender (identity/presentation), and sexual orientation and race, ethnicity and racism.

Reporting on sex and gender	Sex and/or gender were not considered in study design. The reasons for lack of sex- and gender-based analysis are that this study aims to focus on the dengue epidemic or outbreak characteristics in different regions of China based on statistical modeling of infectious diseases and to estimate the corresponding transmission parameters. We aim to assess the burden of the disease in different temporal and spatial dimensions, without of the differences by gender.
Reporting on race, ethnicity, or other socially relevant groupings	This present study did not report on race, ethnicity, or other socially relevant groupings.
Population characteristics	There were a total of 95,339 dengue fever cases across 337 cities in mainland China during 2013-2020. We extracted data on dengue fever including information of dengue fever cases includes basic demographic information (age, present residential address, etc.), disease diagnosis (probable cases, clinically diagnosed cases, and laboratory-confirmed cases), date of disease onset, date of diagnosis, and date of death (if applicable). The daily dengue fever cases were aggregated to each city.
Recruitment	This is a nationwide study based on disease surveillance data on daily incidence of dengue fever during the period from 2013 to 2020. All dengue fever cases were diagnosed according to the diagnostic criteria for Dengue Fever (WS 216-2008 and WS 216-2018) based on criteria issued by the National Health Commission of China, which is responsible for establishing and regularly updating diagnostic criteria for various infectious diseases. All the dengue fever cases were mandated to be reported by the law.
Ethics oversight	Study approval was obtained from the Ethics Committee of the National Institute Communicable Disease Control and Prevention Chinese Center for Disease Control and Prevention (Ethical Approval NO. ICDC-2016008).

Note that full information on the approval of the study protocol must also be provided in the manuscript.

Field-specific reporting

Please select the one below that is the best fit for your research. If you are not sure, read the appropriate sections before making your selection.

☐ Life sciences ☐ Behavioural & social sciences ☒ Ecological, evolutionary & environmental sciences

For a reference copy of the document with all sections, see nature.com/documents/nr-reporting-summary-flat.pdf

Ecological, evolutionary & environmental sciences study design

All studies must disclose on these points even when the disclosure is negative.

Study description	This study aimed to quantify the epidemiological characteristics underpinning the seasonal epidemics and outbreaks of dengue fever in space and time in China. We performed this large-scale study to characterize the epidemiology of dengue fever based on national surveillances. A city-resolved metapopulation network model was used to simulate the transmission of dengue infection among the 337 cities in China. Our findings provide the most comprehensive quantification to date of the time-evolving epidemiological features and burden associated with successive epidemics waves of dengue fever in China over the period of 2013-2020.
Research sample	Data collection is from five different data sources. First, a total of 95,339 case records of dengue fever from 337 cities during 2013-20 were collected. The dengue fever case records span a period of 8 years, from 1st January, 2013 to 31st December, 2020. Surveillance data on dengue fever were collected by the National Notifiable Diseases Surveillance System (NNDSS). Second, data on mosquito density based on 89 survey sites for mosquito vector were obtained from the Chinese Center for Disease Control and Prevention (China CDC). Third, daily meteorological data including ambient temperature, daily maximum temperature and daily minimum temperature during the study period were obtained from the China Meteorological Data Sharing System (http://data.cma.cn/). This dataset comprises information collected from 2,441 meteorological stations in mainland China. Fourth, demographic and population mobility data was publicly obtained from the National Bureau of Statistics of China (http://www.stats.gov.cn/). Fifth, daily city-level population migration data were obtained from the Gaode Migration Big Data platform (https://trp.autonavi.com/migrate/page.do).
Sampling strategy	This is a nationwide disease surveillance dataset in China from 2013 to 2020.
Data collection	Data collection is from five different data sources.
Timing and spatial scale	Dengue fever data were collected from 2013 to 2020 in 337 cities in mainland China. Daily meteorological data during the same study period were obtained from the China Meteorological Data Sharing System. Daily city-level population migration data were publicly available from the Gaode Migration Big Data platform.

Data exclusions	No data were excluded from these analyses.
Reproducibility	Codes are publicly available at https://github.com/tinkzzz/DENGUE_CHINA.git.
Randomization	Not applicable. We used population level surveillance data and did not define different groups.
Blinding	Not applicable. We used population level surveillance data and blinding is not needed.

Did the study involve field work? ☐ Yes ☒ No

Reporting for specific materials, systems and methods

We require information from authors about some types of materials, experimental systems and methods used in many studies. Here, indicate whether each material, system or method listed is relevant to your study. If you are not sure if a list item applies to your research, read the appropriate section before selecting a response.

Materials & experimental systems

n/a	Involved in the study
☒	☐ Antibodies
☒	☐ Eukaryotic cell lines
☒	☐ Palaeontology and archaeology
☒	☐ Animals and other organisms
☒	☐ Clinical data
☒	☐ Dual use research of concern
☒	☐ Plants

Methods

n/a	Involved in the study
☒	☐ ChIP-seq
☒	☐ Flow cytometry
☒	☐ MRI-based neuroimaging

Plants

Seed stocks	N/A
Novel plant genotypes	N/A
Authentication	N/A
